# Comprehensive Comparison of Molecular Fragmentation Schemes for Proteins

**DOI:** 10.1021/acs.jctc.5c01949

**Published:** 2026-03-21

**Authors:** Katharina Rüther, Ken Bunge, Lasse M. Hilmer, Janine Hellmers, Carolin König

**Affiliations:** Institute of Physical Chemistry and Electrochemistry, 26555 Leibniz University Hannover, Callinstr. 3A, 30167 Hannover, Germany

## Abstract

Conventional quantum chemical (QC) methods exhibit a steep computational scaling with respect to the number of atoms in the investigated system. Hence, working with larger systems like peptides or even proteins becomes computationally unfeasible with traditional QC methods. One way to overcome this challenge is through molecular fragmentation methods. Many different flavours of molecular fragmentation schemes based on different partitionings have been suggested in the literature, but have hardly been compared numerically. Our group has recently reported a common formalism for molecular fragmentation schemes, which enables a consistent benchmark of different approaches. Here, we assess the performance of the molecular fractionation with hydrogen caps (MFHC), the pair–pair approximation to the generalized many-body expansion (pp–GMBE), the molecules-in-molecules (MIM) approach, and the kernel energy method (KEM) within this general framework. Our benchmark includes single- and multilevel schemes as well as an electrostatic embedding of the fragments in point charges of the whole system. The energies and computational demand of a chosen set of proteins are evaluated with the different methods within the framework. This enables a rare numerical comparison between the different schemes. Of the compared methods, our implementation of pp–GMBE yields the best agreement with supermolecular QC reference calculations, while MFHC with additional pair couplings offers a good cost–accuracy ratio.

## Graphical abstract

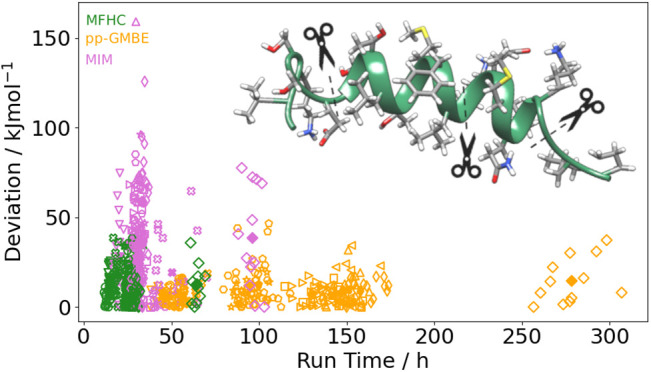

## Introduction

Computational chemistry is nowadays indispensable in chemical research. To achieve chemical accuracy, quantum-chemical methods are typically required. Traditional quantum-chemical methods, however, suffer from a steep computational scaling, preventing their application to large molecular systems such as proteins. Within the recent years, different approaches to beat this computational scaling have been proposed. Many of them exploit the often cited “near-sightedness of matter”^1^ leading to “divide-and-conquer” strategies. Here, the full molecular system is divided into small entities treated with accurate, but computationally costly quantum chemical methods, whereas the longer-distance interactions are treated on a more approximate level. Among the many different flavours of “divide-and-conquer” methodologies (for an overview see ref 2), energy-based molecular fragmentation schemes^3–8^ are the conceptually likely most simple approaches: A molecular system is cut into small entities, often called fragments. These fragments may overlap. The total energy is then obtained as a linear combination of those individual energies. Besides reducing the computational scaling, molecular fragmentation approaches also enable straightforward embarrassingly parallel implementations. The coefficients of this expansion may be obtained by many-body theory or via an inclusion–exclusion principle, though both strategies can yield identical energy expressions.^8–14^

Since this general setting leaves a lot of freedom to choose the fragments and their overlap, many different flavours of energy-based molecular fragmentation schemes are reported in literature.^3,6,7,15^ Even for the specific application of proteins, a large number of different fragmentation approaches are reported, including molecular fractionation with conjugate caps (MFCC),^16–19^ pair–pair generalized many-body expansion (pp–GMBE),^20^ molecular fractionation with hydrogen caps (MFHC),^8,21^ as well as specialized forms of the molecules-in-molecules (MIM)^22–24^ and kernel energy method (KEM)^25^ schemes. Additionally, other molecular fragmentation schemes like the fragments-in-fragments (FIF) approach^26^ and the fragmentation molecular orbital (FMO) method^27^ have been applied for the calculation of proteins. FIF defines a fragmented multilevel scheme similar to its parent method, the molecular tailoring approach (MTA).^26,28–30^ In both MTA and FIF, protein fragmentation is, to the best of our knowledge, performed manually.^26,29^

When fragmenting proteins, dangling bonds occur. Most of the above-mentioned methods apply capping atoms to handle these boundary regions. In contrast to that, the FMO method uses localization approaches to localize the molecular orbitals in the fragments and does not require capping atoms.

Since simple fragmentation approaches typically suffer from slow convergence with coupling level,^5,7,8,12^ they are often combined with multilevel or embedding ideas.^8,20,23,31–35^ In multilevel schemes, the fragmentation expansion is partitioned into different parts calculated on different levels of theory,^8,23,27,36^ while in embedding approaches, the fragments surrounding is accounted for by embedding potentials.^20,31–35,37–39^ The latter may require a correction for double counting of interactions. Both approaches accelerate the convergence, with only a slight increase of the computational cost for a certain expansion. They further add even more choices for parametrizing fragmentation schemes.

Despite the many different molecular fragmentation schemes reported in literature, cross-comparisons between these schemes are rare, presumably due to rather specialized implementation.^8,9,11,40–43^ Our recently established so-called FCR framework for molecular fragmentation schemes is so versatile that it can straight-forwardly implement most above-mentioned schemes (and many more) using capping atoms. This gives us the opportunity to benchmark these models in a consistent manner for a wide range of peptide and protein structures, as reported on in this work. Another platform with a similar goal is the recently released FRAGME∩T code by Herbert and co-workers, which is based on the GMBE algorithm.^15^

In this work, we focus on molecular fragmentation schemes that have been particularly developed for peptides and proteins, provide an automatable fragmentation, and apply capping atoms for dangling bonds. We further choose fragmentation schemes that offer mechanisms to systematically improve the computational setup, either by enlarging the initial fragments^22–24^ or by introducing a many-body expansion on top of the original overlapping fragmentation.^8,20,44^ More precisely, we compare the MFHC,^8^ the pp–GMBE^20^ as well as the protein-specific version of the MIM^22–24,37^ and the KEM^25^ approaches. We evaluate their performance (accuracy vs computational cost) on a wide range of peptides and proteins. We refrain from including the popular MFCC schemes as we have shown the need for higher-order couplings in order to achieve desired accuracy, which leads to rather complex fragmentation expansions and longer run times.^8^ In ref 8, we found that the improvement obtained by the MFCC schemes compared to MFHC does not warrant the increased run times. An alternative coupling scheme^44^ is disregarded as it implements additional approximations based on the many overlapping body expansion (MOBE)^13^ that prevent convergence toward the exact limit^42^ and is thereby not supported by the FCR scheme in the present form.

Despite the fact that in their original formulation MFHC^8^ and MIM^22–24^ were combined with multilevel schemes whereas pp-GMBE includes electrostatic embedding^20^ and KEM^25^ does not include any of these options, we assess multiscale and electrostatic embedding for all schemes. We note that electrostatic embedding in conjunction with MIM has been reported earlier for molecular clusters.^37^ With the resulting molecular fragmentation schemes for proteins, we assess relative and absolute energies of in total 27 peptides and proteins of different size and secondary structure in in total 237 conformations. This study presents to the best of our knowledge the first and most comprehensive cross-comparison of energy-based molecular fragmentation schemes for peptides and proteins.

In this article, we start with an overview over the FCR-based fragmentation introduced in ref 8, as well as the four fragmentation schemes MFHC,^8^ pp–GMBE,^20^ MIM,^22–24,37^ and KEM.^25^ After stating the computational details, we proceed with the results section. There, we analyze the performance of different fragmentation methods for the example protein system 1WN8 with regard to relative energies and run times in depth. Subsequently, we compare relative and absolute energies as well as run times for an additional 26 proteins. We close with a summary of the main conclusions and the outlook of this work.

## Fragmentation Models and Implementation

### FCR-based Fragmentation Framework

Our computations are based on the general framework for molecular fragmentation schemes introduced in ref 8. In this fragmentation formulation, the total energy is expressed as

1 $$E=\sum_{f\in \mathrm{FCR}}p_{f}^{\mathrm{FCR}}E_{f}$$

where **f** is a *fragment combination*, that is a set of fragments. FCR stands for fragment combination range that collects all fragment combinations. *p*_**f**_^FCR^ is the coefficient for the fragment combination **f** in the given FCR and *E*_**f**_ its energy. For more information on how *p*_**f**_^FCR^ is obtained, we refer to refs 8 and 21.

Different fragmentation schemes can be reproduced in this framework. They differ by different choices of initial fragmentation and FCR. Below, we briefly review the different molecular fragmentation schemes for proteins included in the present benchmark. We further sketch how they can be formulated within the FCR-based fragmentation framework.

In the description of the FCR, we use the following terms

**Initial disjoint fragments** are the first chosen fragments. Every atom in the system is assigned to exactly one initial disjoint fragment.

**Initial overlapping fragment combinations** are sets of *initial disjoint fragments*. Each atom can be assigned to more than one initial overlapping fragment combination. Initial overlapping fragment combinations are typically chosen as chemically intuitive covalently bound entities. Choosing overlapping fragment combinations allows to include interactions between initial disjoint fragments such as covalent bonds in the energy expression.

**Final overlapping fragment combinations** are either initial overlapping fragment combinations or combinations of those. By combining initial overlapping fragments also noncovalent and long-range interactions can be considered. Dangling bonds on the final overlapping fragment combinations are capped with hydrogen bonds.

To uniquely define fragment combinations, the FCR framework applies its own nomenclature (c.f. Figure 1).^8^ The general notation follows IF-[CS1]­[CS2]···, which defines initial fragments and coupling schemes alike. IF defines the chosen, possibly overlapping, initial fragment combinations. Those could for example be individual molecules (MOL) or molecule pairs (PAIR). The following [CS*X*]-terms define coupling schemes that apply for different levels of electronic structure theory. If only one of these terms is defined, the calculation utilizes a single level. For multilevel approaches, the [CS*X*]-terms are given in a series starting from the approach using the highest level of theory. The terms define coupling schemes that include hierarchical and neighbor schemes. For the hierarchical scheme, the notation Hier*Y* is used, with *Y* as the order of the expansion. In the neighbor scheme, Nei*Y*_*d*_ is used, where *Y* is again the order and *d* is the closest atom–atom distance cutoff (in Å) for the combination of neighboring fragments. The notation MOL-[Nei2_4.0_]­[Hier2] therefore describes a multilevel approach, where the initial fragments are individual molecules. The higher level of theory is used to calculate FCs combined in the neighbor scheme of second order up to an atom–atom distance cutoff of 4.0 Å, a hierarchical scheme of second order is defined for the lower level. Instead of using a different coupling scheme for the lower level of theory, it is also possible to employ a supersystem calculation, denoted by *Super*. If electrostatic embedding is used, this can be shown by adding an *ee* to the bracket, for example MOL-[Nei2_4.0_ee].

**Figure 1:**
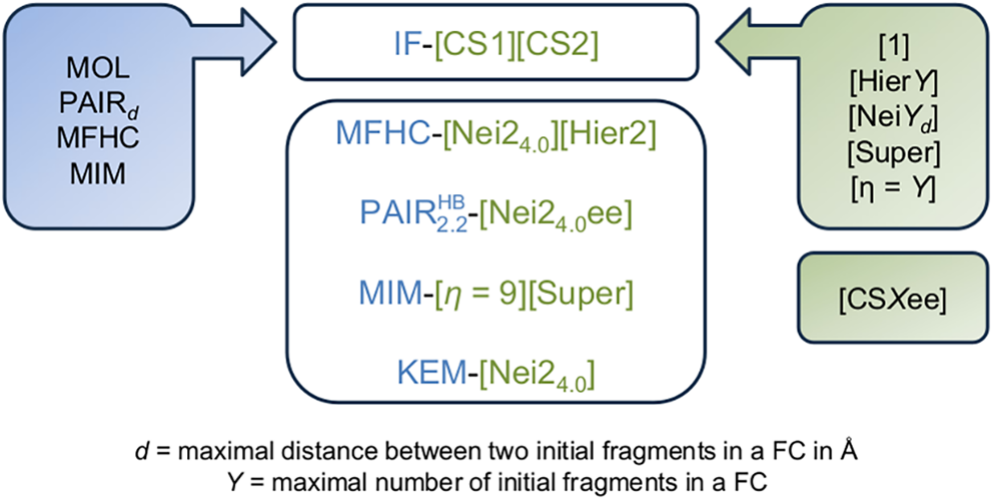
Nomenclature in the FCR framework.

### Molecular Fractionation with Hydrogen Caps (MFHC)

In the MFHC scheme,^8^ the initial disjoint fragments are formed by cutting the bonds connecting the peptide bonds (HN–CO) to the C_α_ carbon atom (c.f. Figure 2). An exception is made for terminating amino acids, here the bonds to the terminating carboxy or amino group are not cut. The MFHC scheme applies special rules for proline and the disulfide bridges cysteine builds. For proline, both N–C-bonds connecting the nitrogen atom with the respective ring are cut, which is marked in yellow in Figure 2. For disulfide bridges, the S–S-bond as well as the two connecting CH_2_ groups form an initial fragment, which is highlighted in green in Figure 2.

**Figure 2:**
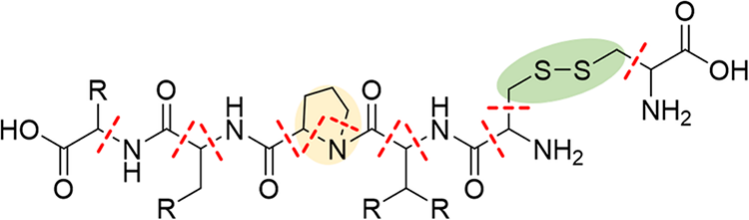
Construction of *initial disjoint fragments* in the MFHC molecular fragmentation scheme, depicted with red lines. Special fragmentation cases for proline and disulfide bridges are highlighted with yellow and green, respectively.

The initial overlapping fragment combinations are then obtained by merging the initial disjoint fragments with a C_α_ carbon atom with all other initial disjoint fragments that are covalently bound to the C_α_ carbon atom (c.f. Figure 3).

**Figure 3:**
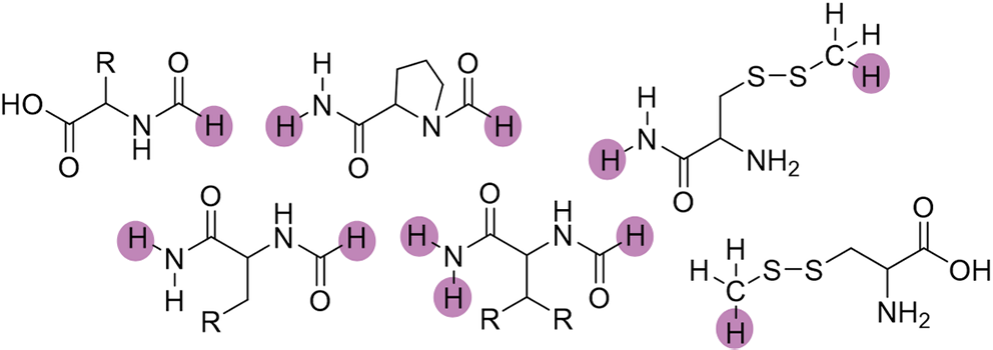
Resulting *initial overlapping fragments* (MFHC-fragments) as combinations of *initial disjoint fragments* in Figure 2 for the MFHC method. All dangling bonds are capped with hydrogen atoms, marked in pink.

The initial overlapping fragments in Figure 3 can subsequently be combined in various ways, for example in the hierarchical or the neighbor scheme. As an example, Figure 4 shows a combination of only direct neighboring initial overlapping fragment combinations to final overlapping fragment combinations, which is also illustrated in ref 21. In general, the combined initial overlapping fragment combinations do not have to be direct neighbors, but a cutoff value can be chosen to control their distance. For example three neighboring initial overlapping fragments may be combined with an atom–atom distance cutoff of 2.2 Å. That would be called MFHC-[Nei3_2.2_] in the FCR nomenclature. To avoid sterical clashes in the capped fragment combinations, fragments may be merged to larger fragments as explained in refs 8 and 21.

**Figure 4:**
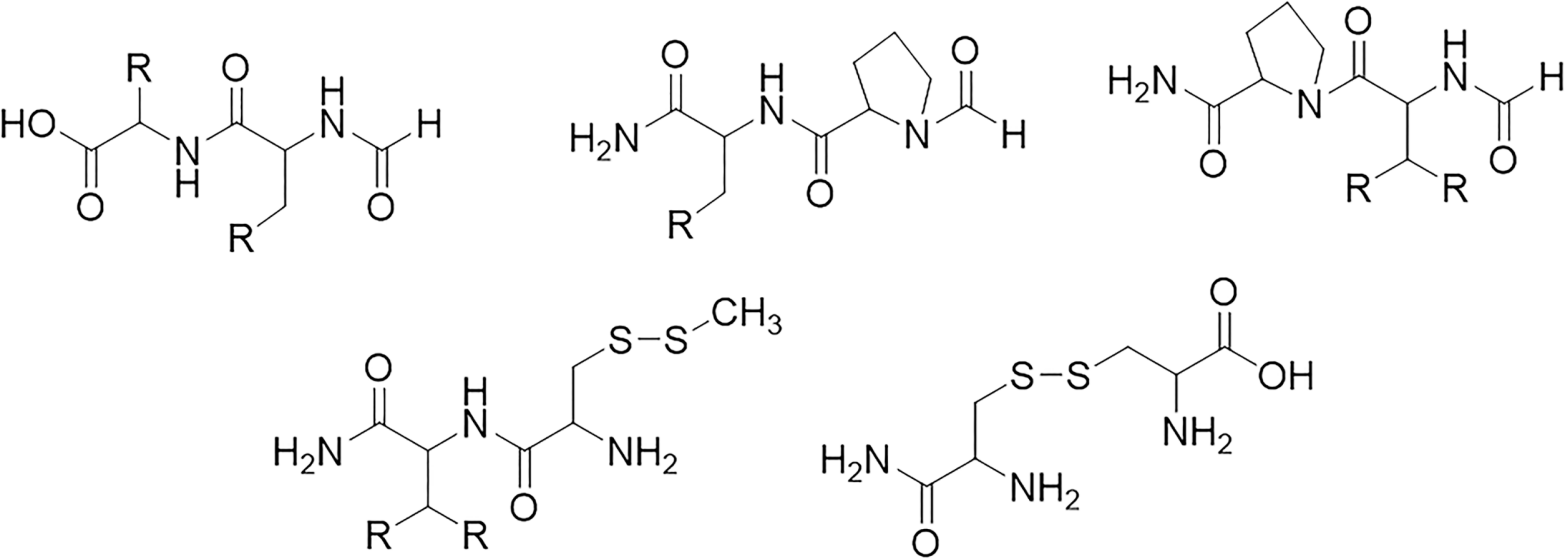
Examples for *final overlapping fragments* as a combination of uncapped *initial overlapping fragments* in Figure 3 for the MFHC method. After combining all dangling bonds are capped with hydrogen atoms.

### Pair–pair Approximation to the Generalized Many-Body Expansion (pp–GMBE)

The pp–GMBE^20^ approach by Herbert and co-workers is based on the GMBE^40^ general formulation of fragmentation methods. Despite GMBE’s foundation in the inclusion–exclusion principle it may be expressed in terms of an FCR.^8^

In pp–GMBE, the initial disjoint fragments are formed by cutting all bonds between C_α_ and C_carbonyl_. This choice keeps the peptide bonds intact. For more cost efficiency, the initial fragments are further restricted to a maximum of 15 atoms, ensuring that the final overlapping fragments have a maximum of 60 atoms. To achieve this, side chains containing more than ten atoms are further partitioned (c.f. Figure 5).

**Figure 5:**
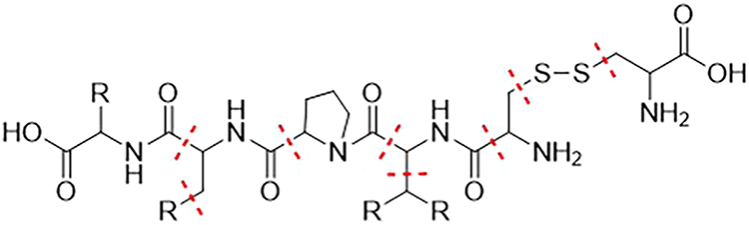
Construction of *initial disjoint fragments* in the pp–GMBE molecular fragmentation scheme, depicted with red lines, based on ref 20

Subsequently, two initial disjoint fragments are combined into an initial overlapping fragment as shown in Figure 6. In line with the FCR nomenclature introduced in ref 8 and Figure 1, we refer to these combinations as PAIR_2.2_^HB^, where 2.2 Å are a threshold for the smallest atom–atom distance. The superscript HB dictates that pairs of initial fragments connected by hydrogen bonds are included in the fragmentation expansion.

**Figure 6:**
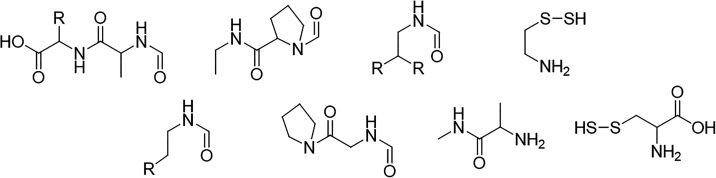
Resulting *initial overlapping fragments* as pairs of the *initial disjoint fragments* in Figure 5 for the pp–GMBE method. All dangling bonds are capped with hydrogen atoms, based on ref 20

Pp–GMBE imposes two requirements for hydrogen bonds: First, the bond length between the hydrogen atom and the electronegative atom forming the hydrogen bond has to be less than twice the sum of both van der Waals radii. Second, the bond angle φ­(X-H···X) has to be larger than 130°. Similarly, our current implementation, mimicking pp–GMBE, considers H–X interactions a hydrogen bond if the H–X distance lies in between 1.7 and 2.2 Å and the φ­(X···H···X) angle >130°. The final overlapping fragments are then formed by combining two initial overlapping fragments up to a atom–atom distance cutoff of 4.0 Å. The combined fragmentation scheme is in the FCR nomenclature denoted as PAIR_2.2_^HB^-[Nei2_4.0_].

It should be noted that the pp–GMBE literature uses a different nomenclature to the FCR approach for the fragment classification. In this work, the FCR nomenclature^8^ (c.f. Figure 1) is followed. The mapping of these two nomenclatures is listed in Table 1. Additionally, the pp–GMBE literature by default includes an electrostatic embedding of the fragment combinations. In this work, single-level and multilevel approaches are supplementarily included for comparison with the other fragmentation schemes.

**Table 1:** Correspondence between the Original MIM, KEM and pp–GMBE Nomenclatures and the FCR Nomenclature

pp–GMBE	FCR	Abbreviation (FCR)
group	initial disjoint fragments	
significant pairs	(initial) overlapping fragments	PAIR_2.2_^HB^-[1]
significant quartets	final overlapping fragments	PAIR_2.2_^HB^-[Nei2_4.0_]

MIM	FCR	
initial fragments	initial disjoint fragments	MIM-[1]
primary subsystems	(initial) overlapping fragments	
derivative subsystems	final overlapping fragments	MIM-[η = *Y*]

KEM	FCR	
kernels	initial disjoint fragments	KEM-[1]
double kernels	final overlapping fragments	KEM-[Nei2_4.0_]

### Molecules-in-Molecules (MIM)

The MIM^22^ approach by Raghavachari and co-workers is a multilevel fragment approach that uses multiple levels of theory to estimate the total energy of a supersystem from fragments of different sizes. The method is similar in spirit to the ONIOM approach,^45^ but considers active fragments throughout the whole investigated molecule.

In the early versions of the MIM scheme,^22,23,46,47^ the initial fragments are formed by cutting all single bonds between heavy atoms (all atoms except hydrogen) in the protein. Peptide bonds are not considered single bonds in this scheme, because of their partial double bond character. In newer versions,^24^ only C–C-bonds to C_α_ are cleaved, as shown in Figure 7. That means the C_α_–C_carbonyl_ bonds are being cut, as well as the side chains starting from the C_α_-atom.

**Figure 7:**
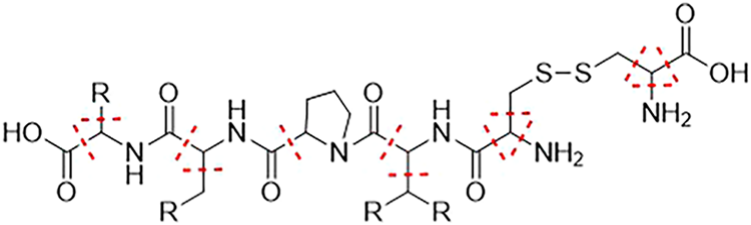
Construction of *initial disjoint fragments* in the later MIM molecular fragmentation scheme, depicted with red lines, based on ref 24.

We here reproduce the more recent MIM model in the FCR framework. For each initial disjoint fragment, the MIM approach defines an initial overlapping fragment by either using a distance- or a number-based cutoff. The distance-based cutoff assigns all fragments to the initial overlapping fragment, that are less than the specified atom–atom distance *d* away from the initial disjoint fragment, that is looked at. Although both the distance- and the number-based approach are implemented in the FCR framework, only the number-based cutoff is used in this work. To form an initial overlapping fragment using a number-based cutoff η, Raghavachari and co-workers start from one initial disjoint fragment and then add η – 1 of the nearest other initial fragments, based on the smallest atom–atom distance. For highly symmetric molecules, a second rule comes in for the number-based cutoff. If an additional fragment has the same atom–atom distance from the initial fragment as the farthest included fragment, that fragment will also be included. Further, each fragment is required to be covalently bound to at least one other fragment in the initial overlapping fragment, which is in our case ensured by a distance threshold of 1.7 Å.

After forming these overlapping fragments, the MIM scheme checks, if there are atoms, which are covalently bound to more than one atom in the initial overlapping fragment. If there are, those are added to the initial overlapping fragment and the process is repeated until no more atoms can be added. This procedure ensures that no atom is substituted by more than one cap aka that the hydrogen caps do not clash. In the next step, initial overlapping fragments, that are subsets to another initial overlapping fragment, are removed. Thereby, final overlapping fragments are formed. All dangling bonds are capped with hydrogen atoms.

The MIM approach again uses a different nomenclature to the FCR approach. In this work, the FCR nomenclature^8^ is followed (c.f. Figure 1). The mapping of the two nomenclatures is listed in Table 1. Because the initial overlapping fragments are not always formed identically, as it is the case for the MFHC and the pp–GMBE, the nomenclature for calculations in the FCR framework has to be adjusted to be used for the MIM scheme. The calculations are denoted as MIM-[η = *Y*], with *Y* being the chosen number-based cutoff, for example MIM-[η = 6] is a combination of six initial disjoint fragments.

### Kernel Energy Method (KEM)

The KEM approach by Huang et al.^25^ was intended for applying quantum crystallography to large molecules. The method defines its initial fragments to be one amino acid in size. Therefore, we, in our implementation of the KEM scheme, form the initial disjoint fragments by cutting all peptide bonds (c.f. Figure 8). Subsequently, two neighboring initial fragments with a maximal distance of 4.0 Å are combined to form the final fragments of the KEM scheme, denoted as KEM-[Nei2_4.0_].

**Figure 8:**
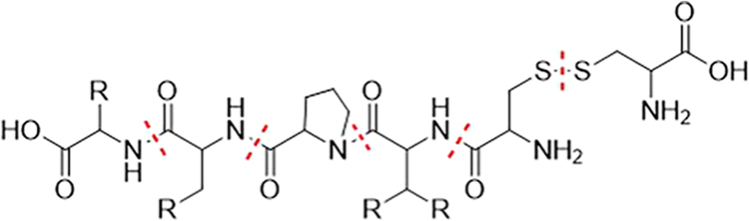
Construction of *initial disjoint fragments* in the KEM molecular fragmentation scheme, depicted with red lines, based on ref 25.

The KEM approach again uses a different nomenclature to the FCR approach. In this work, the FCR nomenclature^8^ is followed (c.f. Figure 1). The mapping of the two nomenclatures is listed in Table 1.

### Capping Scheme

Independent of the used approach, a fragmentation scheme has to deal with the dangling bonds after cutting the fragments. Most use hydrogen atoms for capping, while some use larger end groups, for example the molecular fractionation with conjugate caps (MFCC)^48^ and its generalized variant (GMFCC),^49^ which uses specialized caps for the covalent and hydrogen-bonding interactions. In the four fragmentation schemes used in this work, hydrogen atoms are used as caps. The hydrogen atoms are added along the broken bonds with fixed bond lengths, which were derived from the Universal Force Field (“UFF”)^50,51^ (see Table 2).

**Table 2:** Used Bond Lengths for Placing Hydrogen Caps

capped atom	distance to cap
C	1.112 Å
N	1.060 Å
O	1.030 Å

Note that also the MFCC scheme can be formulated with only hydrogen capping atoms using the FCR framework, as shown in ref 21.

### Multilevel Schemes

The FCR approach opens up the possibility of not only using single-level (SL) approaches, but also different electronic-structure setups for different fragments.^8^ In those multilevel (ML) approaches, the total energy *E* ^ML^ of the FCR is expressed as the sum of low-level (LL) and high-level (HL) contributions in accordance with

2 $$E^{\mathrm{ML}}=\sum_{f\in \mathrm{FCR}\left(\mathrm{HL}\right)}p_{f}^{\mathrm{FCR}\left(\mathrm{HL}\right)}E_{f}^{\mathrm{HL}}+\sum_{f\in \mathrm{FCR}\left(\mathrm{LL}\right)}p_{f}^{\mathrm{FCR}\left(\mathrm{LL}\right)}E_{f}^{\mathrm{LL}}$$

3 $$p_{f}^{\mathrm{FCR}\left(\mathrm{HL}\right)}+p_{f}^{\mathrm{FCR}\left(\mathrm{LL}\right)}=p_{f}^{\mathrm{FCR}\left(\mathrm{LL}\right)\cap \mathrm{FCR}\left(\mathrm{HL}\right)}$$

HL marks the fragments considered with a higher level of theory, LL the fragments whose energies are calculated with a more cost efficient method. The expression can straight-forwardly be expanded to more levels. For more information on how the coefficients *p*_**f**_ are obtained, see ref 8.

In the nomenclature of calculations making use of multilevel approaches, a second bracket is utilized (c.f. Figure 1). This could for example lead to MFHC-[Nei2_4.0_]­[Hier2] or PAIR_2.2_^HB^-[Nei2_4.0_]­[Super], where the first bracket represents calculations using the higher level of theory and the second bracket represents calculations using the lower level of theory. The [Hier2] denotes a hierarchical combination of fragments using second order, while [Super] denotes a supermolecular calculation. For more details, see ref 8.

### Electrostatic Embedding

Another possibility to efficiently account for long-range interactions in the FCR framework is the use of electrostatic embedding (EE).^20,37^ In this work, the electrostatic embedding is implemented similarly to the electrostatic embedding in the pp–GMBE approach.^20^ This means every fragment calculation includes point charges of the complete remaining system. In contrast to the pp–GMBE scheme, we here, however, apply Mulliken charges rather than charges obtained from the natural population analysis (NPA) as rationalized in the Computational Details below. The charge embedding can be viewed as a specific variant of a multilevel method.^20^ In contrast to the above-mentioned multilevel approach, charge embedding affects the individual fragment combination calculations.

The charges for the embedding are derived from smaller FCRs. In the scope of this work, these FCRs are MFHC-[1] for the MFHC scheme, PAIR_2.2_^HB^-[1] for the pp–GMBE scheme, MIM-[η = 3] for the MIM scheme, and KEM-[1] for the KEM scheme, applying the same electronic-structure method as for the fragment calculations themselves. This avoids a reliance on supermolecular calculations that are impractical for large systems and high levels of electronic-structure theory. For each atom, the charges are calculated and summed according to the coefficients of the respective FCs and then saved as point charges. To minimize the error in the total charge that might occur from the hydrogen caps not perfectly canceling, these charges are redistributed to the atoms that the caps replace. This approach is consistent with the EE implementation in the original pp–GMBE scheme and results in only minor changes in the atomic charges compared, while maintaining the accurate total charge. The same small FCR is then set up again with the modification that each calculation for a fragment combination incorporates the point charges of all atoms not explicitly included in that fragment combination as embedding charges. The charges from these calculations are then used as new embedding charges. This process is carried out iteratively. We apply two iterations, which we found to be a good compromise between accuracy of the charges and computational feasibility. The final point charges can subsequently be employed as embedding charges for any desired FCR. Finally, the double counting of the Coulombic interaction of the embedding charges (included in each fragment calculation) needs to be accounted for with a Coulomb correction. The final result is obtained as

4 $$E\approx \sum_{f\in \mathrm{FCR}}p_{f}^{\mathrm{FCR}}E_{f}^{\mathrm{ee}}-\left[\left(\sum_{f\in \mathrm{FCR}}p_{f}-1\right)\right]\cdot E_{\mathrm{Coul}}$$

where *E* represents the total energy of the system, *E*_**f**_^ee^ is the energy of the FCR, when the double counting is not accounted for, and *E*_Coul_ is the energy founded in the Coulomb interaction (Coul) arising from the mutual interaction of all charges.

In the nomenclature of calculations making use of electrostatic embedding, an ee is added into the bracket, this for example results in PAIR-[Nei2_4.0_ee] (c.f. Figure 1).

## Computational Details

All fragment calculations were carried out using a Python3 based library named FragPy developed for the FCR framework by the König group.^8^ The initial fragmentation was done with FragIt,^52^ where certain bonds can be addressed in the SMARTS language. After assembling the FCR from that fragments, the energies were calculated with ORCA (version 5.0.4)^53,54^ and PBEh-3c^55^ and HF-3c,^56^ respectively, and default convergence parameter. These methods include a geometrical counterpoise (gCP) correction^57^ to counteract the basis set superposition errors occurring for fragmentation approaches.^58–61^ To investigate the influence of the electronic structure method, the hybrid functional B3LYP^62–64^ with the basis set def2-TZVP^65,66^ was used. For the calculations using B3LYP, the dispersion correction D3 of the Grimme group was used with Becke–Johnson damping.^67,68^ Additionally, a gCP correction was employed.^57^ Mulliken charges are employed as embedding charges, which are always calculated using the high level of electronic-structure calculation. While Mulliken charges work surprisingly well in many fragmentation applications,^5,31,32,69–71^ we note that they may cause problems with respect to basis set expansion, as reported in ref 72. For this reason pp-GMBE^20^ and others^73,74^ apply charges from NPA. Due to the small basis sets in most applied electronic structure methods and their availability in many electronic structure programs, Mulliken charges were taken as a pragmatic choice in the present study.

The structures of most investigated proteins were taken from the protein data bank (PDB). They were then optimized using the program GROMACS^75–78^ with the AMBER99SB force-field.^79^ Exceptions are 1CTF, 1UBQ, 1FKF, 3I40, and 3SEM, whose optimized structures were taken from a publication of Vornweg et al.^44^ The systems were chosen according to a wide range of sizes and structures to test the FCR frameworks functionality for arbitrary protein systems. The rather small protein 1WN8 was used to benchmark the different fragmentation methods in a timely manner. Eleven further proteins were also investigated in depth to validate the findings for relative energies on larger proteins. Finally, 15 other proteins with up to over 2200 atoms were chosen as test systems to showcase the applicability of the framework for absolute energies and larger system sizes.

All reported timings were obtained using Intel­(R) Xeon­(R) Silver 4316 CPU (2.30 GHz). The reported run times refer to the sum of the walltimes of the individual fragment calculations. The computing time for assembling the FCR is not included in the run times.

## Results and Discussion

In this work, we employ the FCR framework to compare fragmentation methods for a selection of protein systems. On the one hand, multilevel and embedding schemes are compared within our implementation of one of the four schemes MFHC, pp–GMBE, MIM, and KEM. On the other hand, our implementations of the MFHC, pp–GMBE, MIM, and KEM schemes are compared among one another.

### In-Depth-Analysis for 1WN8 Conformers

We chose the protein 1WN8 to investigate the performance of some fragmentation methods in comparison to supermolecular reference calculations. For all 20 different conformers of 1WN8 reported in the PDB, we assessed the fragmentation schemes listed in Table 3. We present our results as relative energies with respect to the lowest-energy conformer in the respective fragmentation scheme or supermolecular calculation.

**Table 3:** Different Fragmentation Schemes for 1WN8 Conformers Compared in This Section^a^

	MFHC	pp–GMBE	MIM	KEM
SL	MFHC-[Nei2_4.0_]	PAIR_2.2_^HB^-[Nei2_4.0_]	MIM-[η = 6]	KEM-[Nei2_4.0_]
	MFHC-[Nei3_2.2_]		MIM-[η = 9]	
ML	MFHC-[Nei2_4.0_][Super]	PAIR_2.2_^HB^-[Nei2_4.0_][Super]	MIM-[η = 6][Super]	KEM-[Nei2_4.0_][Super]
	MFHC-[Nei3_2.2_][Super]		MIM-[η = 9][Super]	
	MFHC-[Nei2_4.0_][Hier2]	PAIR_2.2_^HB^-[Nei2_4.0_][Hier2]		KEM-[Nei2_4.0_][Hier2]
	MFHC-[Nei3_2.2_][Hier2]			
EE	MFHC-[Nei2_4.0_ee]	PAIR_2.2_^HB^-[Nei2_4.0_ee]	MIM-[η = 6ee]	KEM-[Nei2_4.0_ee]
	MFHC-[Nei3_2.2_ee]		MIM-[η = 9ee]	

a Fragmentation methods sharing the same line are similar in their FC sizes and can therefore be compared regarding their accuracy. We discriminate here between single-level (SL), multilevel (ML) and electrostatically embedded (EE) calculations.

#### MFHC

Figure 9 correlates the relative energies of 1WN8 obtained with different MFHC-based fragmentation setups to the supermolecular reference calculation. Table 4 lists the associated calculated mean absolute deviations (MADs) and root-mean-square deviations (RMSDs).

**Figure 9:**
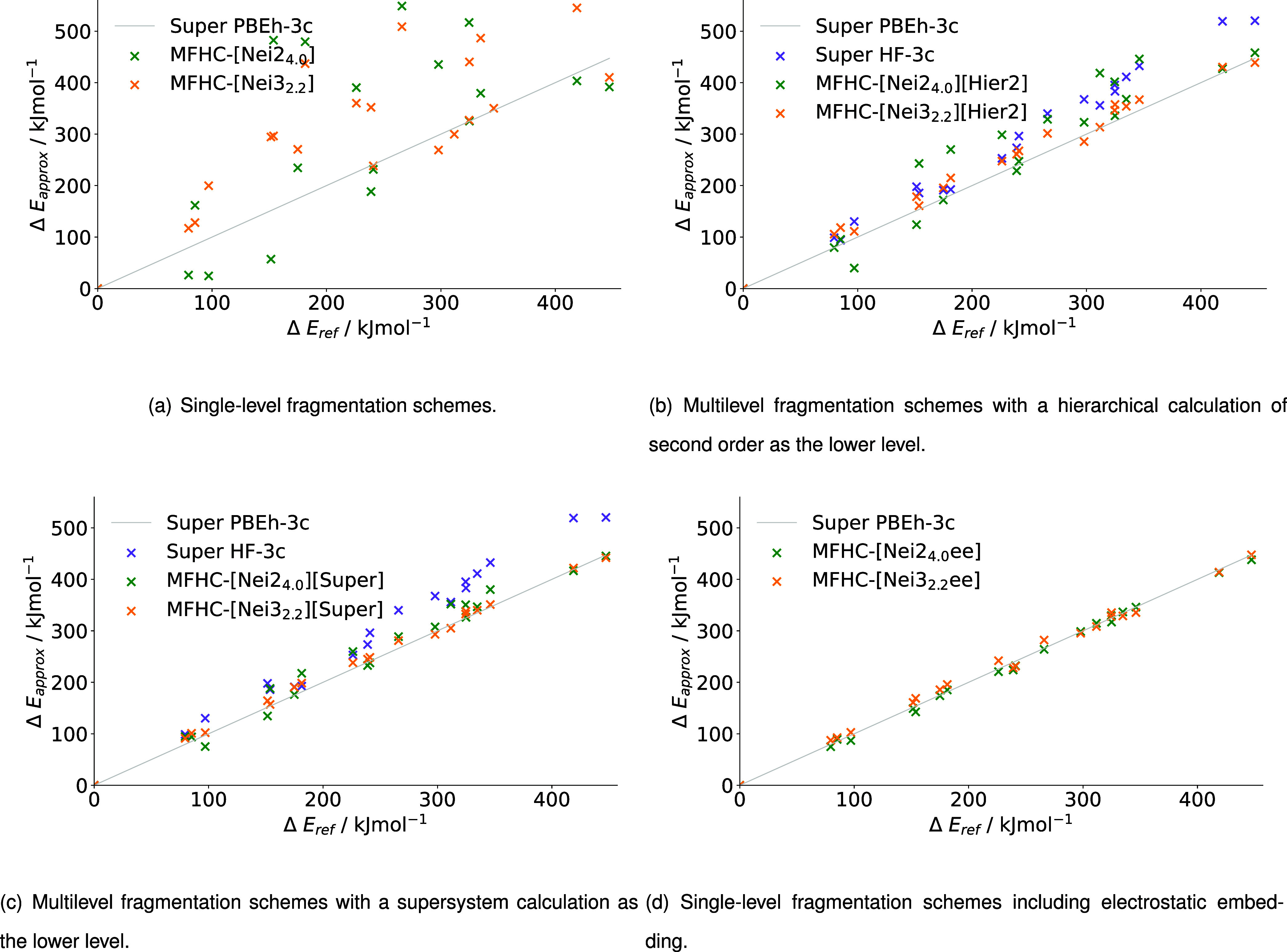
Relative energies of MFHC (a) single-level fragmentation schemes, (b) multilevel fragmentation schemes with a hierarchical calculation of second order as the lower level, (c) multilevel fragmentation schemes with a supersystem calculation as the lower level and (d) single-level fragmentation schemes including electrostatic embedding for the relative energies of 20 conformers of 1WN8 calculated with PBEh-3c as higher level and HF-3c as lower level, where applicable.

**Table 4:** MADs and RMSDs of the Relative Energies of 1WN8 Conformers Obtained through MFHC-Based Fragmentation Methods

	method	MAD/kJ mol^–1^	RMSD/kJ mol^–1^
**SL**	MFHC-[Nei2_4.0_]	153.34	212.09
	MFHC-[Nei3_2.2_]	118.51	148.95
**ML**	MFHC-[Nei2_4.0_][Hier2]	36.40	50.21
	MFHC-[Nei3_2.2_][Hier2]	26.42	30.06
	MFHC-[Nei2_4.0_][Super]	17.10	22.72
	MFHC-[Nei3_2.2_][Super]	12.31	14.43
**EE**	MFHC-[Nei2_4.0_ee]	6.76	8.33
	MFHC-[Nei3_2.2_ee]	8.13	9.63

For single-level methods, we find a rather poor correlation of the fragmentation results to the supermolecular reference (c.f. Figure 9 (a)). The energies are mostly overestimated in comparison to the supermolecular reference, with only the conformers at 240.96 kJ mol^–1^ and 418.89 kJ mol^–1^ lying close to the reference for MFHC-[Nei2_4.0_] and the conformers at 240.96 kJ mol^–1^, 324.65 kJ mol^–1^, and 346.21 kJ mol^–1^ lying close to the reference for MFHC-[Nei3_2.2_]. For MFHC-[Nei3_2.2_] we obtain an MAD of 118.51 kJ mol^–1^ and for MFHC-[Nei2_4.0_] we obtain an MAD of 153.34 kJ mol^–1^, reflecting that these results are clearly not in desired agreement to the supersystem reference.

By applying a second level of theory (ML) or electrostatic embedding (EE), the results can be improved significantly (c.f. Figure 9 (b)–(d)), going from an MAD of 153.34 kJ mol^–1^ for the most inaccurate method MFHC-[Nei2_4.0_] to MADs well below 40 kJ mol^–1^ for all ML and EE methods. The multilevel fragmentation schemes with a hierarchical calculation of second order as the lower level in Figure 9 (b) still show rather large deviations from the supersystem results, without completely reproducing the general trend. For the MFHC-[Nei2_4.0_]­[Hier2] scheme in particular, the relative energies deviate from the reference by up to 100 kJ mol^–1^. Nevertheless, the relative energy graphs acquired with hierarchical lower levels are in better agreement with the reference than the single-level approaches. While the MADs and RMSDs are halved going from the hierarchical to the supermolecular lower level, the trends of the graphs show very similar outliers, with the difference that they are significantly less pronounced with the supermolecular lower level. Examples for these outliers include the conformers at 97.03, 153.71, 226.11, and 311.70 kJ mol^–1^ for the MFHC-[Nei2_4.0_] ML schemes and at 265.96 and 324.92 kJ mol^–1^ for the MFHC-[Nei3_2.2_] ML schemes. The results for the MFHC-[Nei3_2.2_]­[Super] method also nicely show the reduced deviation of outliers compared to the MFHC-[Nei3_2.2_]­[Hier2] scheme. The results only deviate from the shown upward trend because of the relative energy of the conformer at 151.48 kJ mol^–1^. To achieve even lower deviations of the relative energies with the MFHC scheme, electrostatic embedding can be utilized. With MADs and RMSDs of less than 10 kJ mol^–1^, both MFHC-[Nei2_4.0_ee] and MFHC-[Nei3_2.2_ee] lead to relative energies that mostly overlap with the energies obtained with the supermolecular reference. It is especially astonishing that the fragmentation method using smaller overlapping fragments (MFHC-[Nei2_4.0_ee]) shows a higher accuracy than MFHC-[Nei3_2.2_ee]. This can presumably be attributed to fortunate error cancellation for MFHC-[Nei2_4.0_ee].

In summary, a multilevel approach seems to be necessary to achieve accurate relative energies in the MFHC scheme. While a significant increase in accuracy can already be achieved by incorporating a hierarchical or supermolecular lower level of theory into the calculations, the electrostatically embedded calculations obtain the most accurate relative energies for the protein 1WN8 when compared to a supermolecular reference.

#### pp–GMBE

Figure 10 represents the relative energies obtained with different fragmentation setups for our implementation of the pp–GMBE scheme. As already shown in Table 3, only methods forming quartets of initial disjoint fragments are compared in our implementation to comply with the rules established for pp–GMBE by Liu and Herbert.^20^ Table 5 lists the associated calculated MADs and RMSDs.

**Figure 10:**
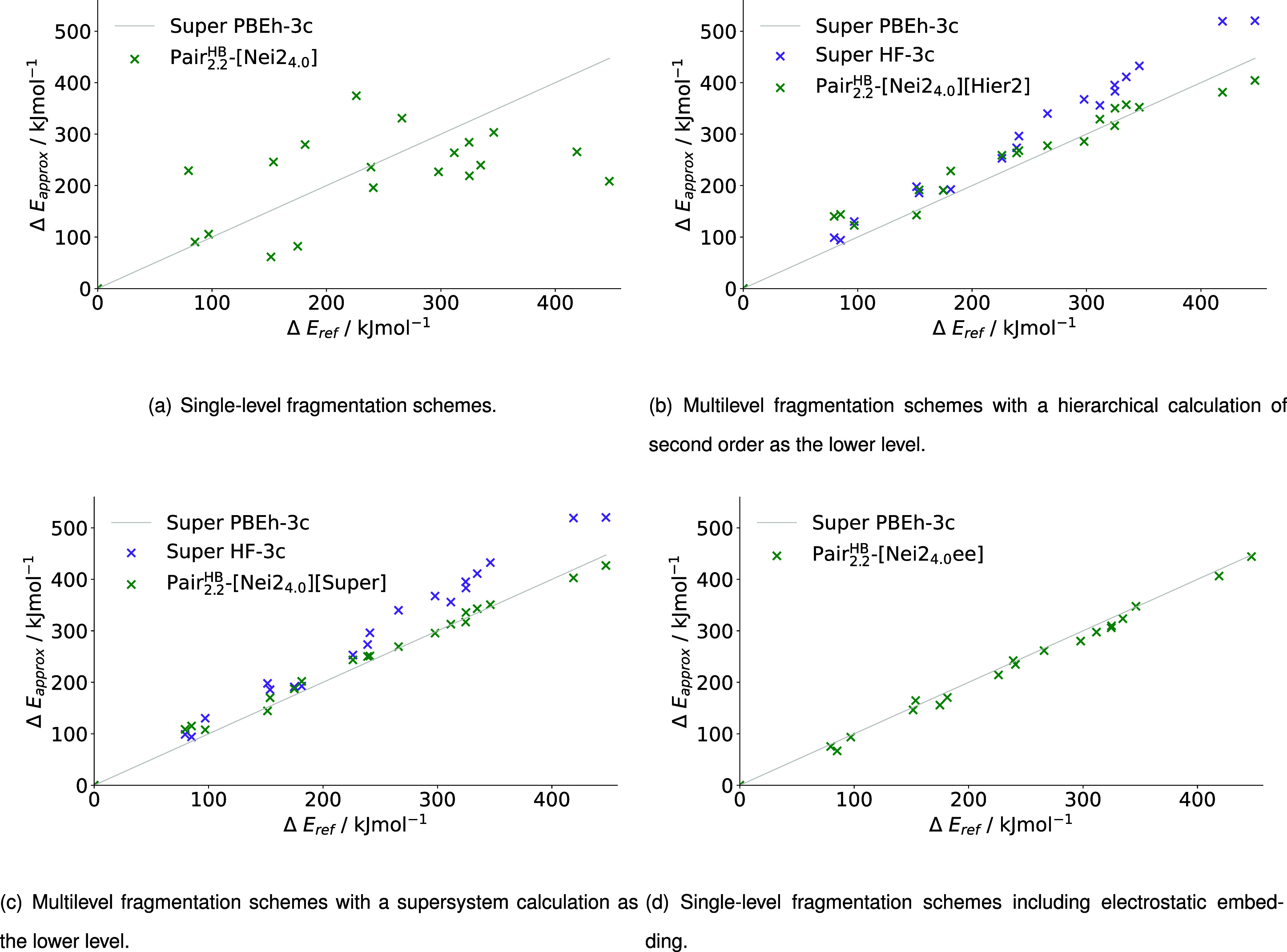
Relative energies of pp–GMBE (a) single-level fragmentation schemes, (b) multilevel fragmentation schemes with a hierarchical calculation of second order as the lower level, (c) multilevel fragmentation schemes with a supersystem calculation as the lower level and (d) single-level fragmentation schemes including electrostatic embedding for the relative energies of 20 conformers of 1WN8 calculated with PBEh-3c as higher level and HF-3c as lower level, where applicable.

**Table 5:** MADs and RMSDs of the Relative Energies of 1WN8 Conformers Obtained through pp–GMBE-Based Fragmentation Methods

	method	MAD/kJ mol^–1^	RMSD/kJ mol^–1^
SL	PAIR_2.2_^HB^-[Nei2_4.0_]	215.82	242.61
ML	PAIR_2.2_^HB^-[Nei2_4.0_][Hier2]	57.98	64.31
	PAIR_2.2_^HB^-[Nei2_4.0_][Super]	27.01	30.74
EE	PAIR_2.2_^HB^-[Nei2_4.0_ee]	7.78	9.75

Similar to above, the single-level method PAIR_2.2_^HB^-[Nei2_4.0_] shows considerable deviations from the supersystem results with an MAD of over 200 kJ mol^–1^ (c.f. Table 5). Multilevel approaches again lead to better agreement with the supermolecular reference. Starting with the multilevel approach with a hierarchical lower level, an MAD of 57.98 kJ mol^–1^ is attained. This is only about one-fourth of the MAD in comparison to the SL approach (c.f. Table 5). Still, the PAIR_2.2_^HB^-[Nei2_4.0_]­[Super] method with a supermolecular lower level can obtain more accurate relative energies with an MAD of 27.01 kJ mol^–1^. While the accuracy of the two ML approaches is quite different when comparing the MADs, the graphs of relative energies show some similar characteristics (c.f. Figure 10 (b), (c)). The largest deviations from the supermolecular reference are obtained for the same conformers: This is especially noticeable for the two conformers at around 80 kJ mol^–1^ as well as the conformers with the highest energies at 418.89 and 447.22 kJ mol^–1^. Nevertheless, the outliers are more prominent in the PAIR_2.2_^HB^-[Nei2_4.0_]­[Hier2] method than for PAIR_2.2_^HB^-[Nei2_4.0_]­[Super].

The methods with electrostatically embedded fragment calculations obtain the most accurate relative energies for the pp–GMBE scheme. With an MAD of only 7.78 kJ mol^–1^, the PAIR_2.2_^HB^-[Nei2_4.0_ee] setup is substantially more accurate in terms of MADs in comparison to the next best method, the ML approach with a supermolecular lower level (c.f. Table 5).

In summary, the PAIR_2.2_^HB^-[Nei2_4.0_] methods lead to similar findings as the MFHC methods: The single-level approaches cannot deliver relative energies of reasonable accuracy for further use. The implementation of multilevel methods improves the accuracy significantly, with a hierarchical lower level being less accurate than a supermolecular lower level. The electrostatically embedded calculations deliver the most reliable relative energies in our calculations with MADs of less than 10 kJ mol^–1^.

#### MIM

Next, Figure 11 and Table 6 show the relative energies and their average errors in our implementation of the MIM scheme. Fragmentation methods including overlapping fragments of different sizes are compared. However, no multilevel calculations with a hierarchical lower level are included (c.f. Table 3), because a hierarchical lower level in the MIM-[η = *Y*] scheme, which contains small initial disjoint fragments, would lead to over double the amount of FCs in comparison to MFHC-[Nei2_4.0_]­[Hier2] or PAIR_2.2_^HB^-[Nei2_4.0_]­[Hier2]. Therefore, a hierarchical lower level would lead to an unfeasible amount of fragment combinations for larger proteins.

**Figure 11:**
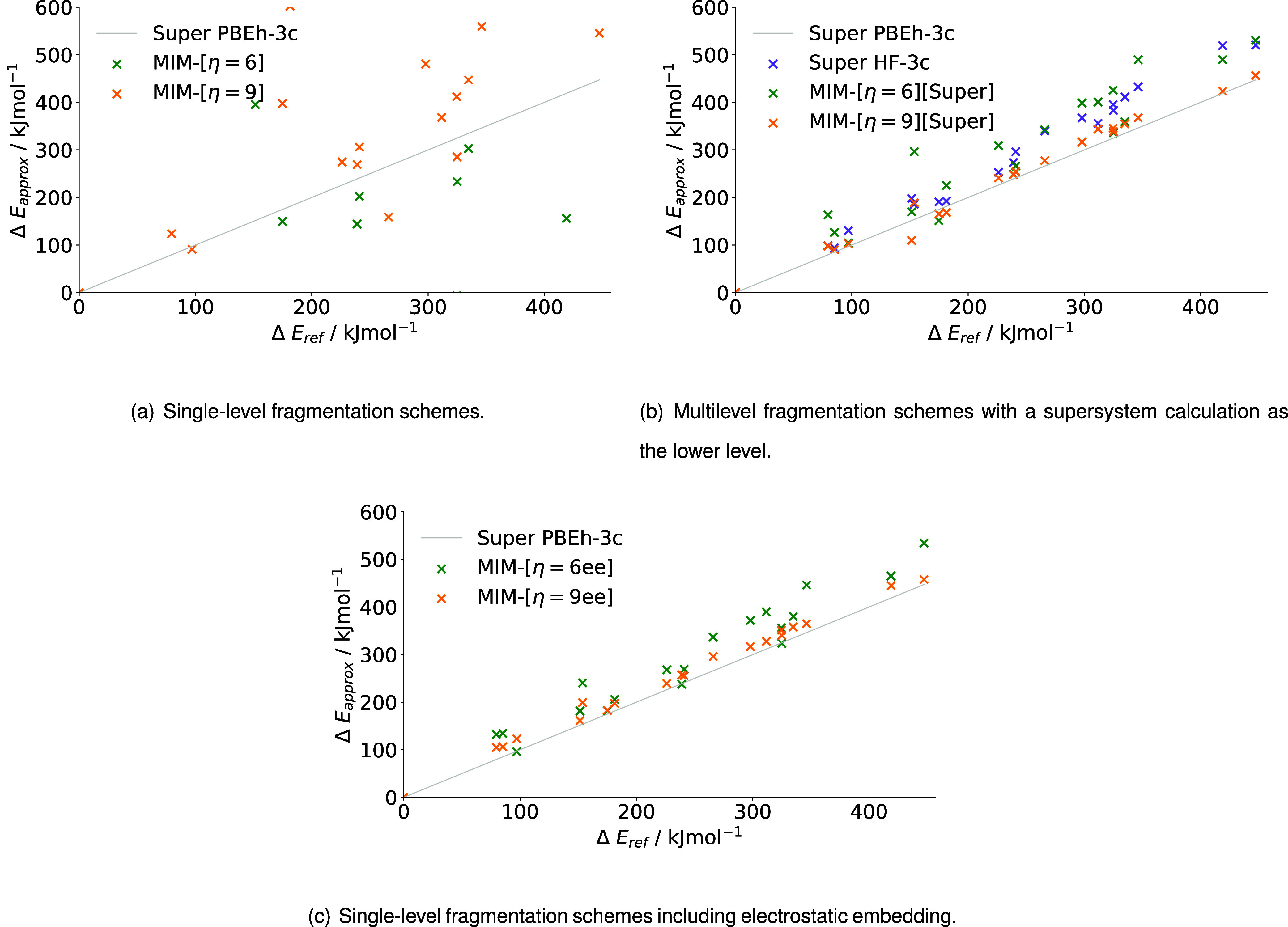
Relative energies of MIM (a) single-level fragmentation schemes, (b) multilevel fragmentation schemes with a supersystem calculation as the lower level and (c) single-level fragmentation schemes including electrostatic embedding for the relative energies of 20 conformers of 1WN8 calculated with PBEh-3c as higher level and HF-3c as lower level, where applicable.

**Table 6:** MADs and RMSDs of the Relative Energies of 1WN8 Conformers Obtained through MIM-Based Fragmentation Methods

	method	MAD/kJ mol^–1^	RMSD/kJ mol^–1^
SL	MIM-[η = 6]	449.73	542.27
	MIM-[η = 9]	149.36	227.86
ML	MIM-[η = 6][Super]	42.66	54.69
	MIM-[η = 9][Super]	11.57	16.86
EE	MIM-[η = 6ee]	45.70	55.94
	MIM-[η = 9ee]	9.92	12.97

The single-level method MIM-[η = 6] exhibits significant deviations from the supersystem results with an MAD above 400 kJ mol^–1^ (c.f. Table 6). Note that Figure 11 (a) does not show all data points due to massive outliers. The complete set of results can be found in Figure S–I in the Supporting Information (SI). The larger overlapping fragment combinations of the MIM-[η = 9] method cut the MAD into one-third, but it is still very inaccurate. The inclusion of a supermolecular lower level leads to significant improvements in the obtained relative energies. Both the MIM-[η = 6]­[Super] and the MIM-[η = 9]­[Super] methods MADs are lower by a factor of 10 in comparison to their single-level counterparts (c.f. Table 6). Taking a closer look at the relative energy graphs in Figure 11, the MIM-[η = 6]­[Super] method shows very irregular relative energies with some outliers that deviate from the supermolecular reference by over 100 kJ mol^–1^. MIM-[η = 9]­[Super] on the other hand leads to energies close to the reference, which reflects in an MAD of only 11.57 kJ mol^–1^. Despite this rather high accuracy, the relative energies of the conformers at 151.48 and 153.71 kJ mol^–1^ as well as 311.70 kJ mol^–1^ deviate slightly from the order in relative energies shown by the reference calculations.

At first glance, the methods involving electrostatically embedded fragment calculations in the MIM-[η = *Y*] scheme do not appear to be more accurate than the ML approaches. Exclusively looking at the MADs in Table 6, the accuracy of the methods utilizing a supermolecular lower level and the methods utilizing electrostatic embedding does not seem to differ significantly, when comparing those methods with the same size of initial overlapping fragments. Nevertheless, the graphs in Figure 11 (c), originating from the electrostatically embedded calculations, are quite different from the graphs for the multilevel schemes in Figure 11 (b). Starting with the MIM-[η = 6ee] method, the graph displays many conformers that strongly deviate from the supermolecular reference. Only the conformers at 97.03, 238.95, and 324.65 kJ mol^–1^ are energetically very close to the reference. While the relative energies of all other conformers differ from the reference significantly, they are less far off the reference than the outliers in the MIM-[η = 6]­[Super] method (c.f. Figure 11). With that, the graph of MIM-[η = 6ee] also presents a smoother trend with less oscillations around the supermolecular reference than MIM-[η = 6]­[Super]. For the MIM-[η = 9ee] method, similar observations can be made. All relative energies obtained with the method are overestimations (c.f. Figure 11 (c)). However, with the exception of the conformer at 153.71 kJ mol^–1^, these overestimation are rather small in value. In contrast, the MIM-[η = 9]­[Super] setup obtains some relative energies that are lower and some that are higher than the reference (c.f. Figure 11 (b)). Overall, the relative energies show a less uniform behavior for the ML approach than for the EE approach. Therefore, the electrostatically embedded methods are once again to be favorably used for the protein 1WN8.

#### KEM

Lastly, Figure 12 and Table 7 show relative energies, MADs and RMSDs in our implementation of the KEM scheme. As already shown in Table 3, only methods forming pairs of initial disjoint fragments are compared in our implementation to comply with the double kernels established for KEM by Huang et al.^25^ The single-level method KEM-[Nei2_4.0_] again exhibits considerable deviations from the supersystem results with an MAD of nearly 200 kJ mol^–1^ (c.f. Table 7). Note that Figure 12 (a) does not show all data points due to massive outliers and the complete set of results can be found in Figure S–I in the Supporting Information (SI). This result can be considerably improved by a second level of theory. Starting with the ML approach with a hierarchical lower level, an MAD of only 27.80 kJ mol^–1^ is attained, which cuts the MAD obtained with the SL method into one-seventh. A supermolecular lower level achieves an even higher accuracy in relative energies with an MAD of 14.98 kJ mol^–1^, nearly cutting the error in half again. Figure 12 (b), (c) visually support the differing MADs and RMSDs. While the conformers relative energies are already rather close to the supermolecular reference for KEM-[Nei2_4.0_]­[Hier2], especially in comparison to the SL approach (c.f. Figure 12 (a)), there are two outliers deviating from the supermolecular reference by about 100 kJ mol^–1^. Namely, its the conformers at about 79.46 and 181.35 kJ mol^–1^. The deviations from the reference are considerably smaller in the KEM-[Nei2_4.0_]­[Super] scheme (c.f. Figure 12 (c)), where all data points can be found within a 50 kJ mol^–1^ error from the supermolecular calculation.

**Figure 12:**
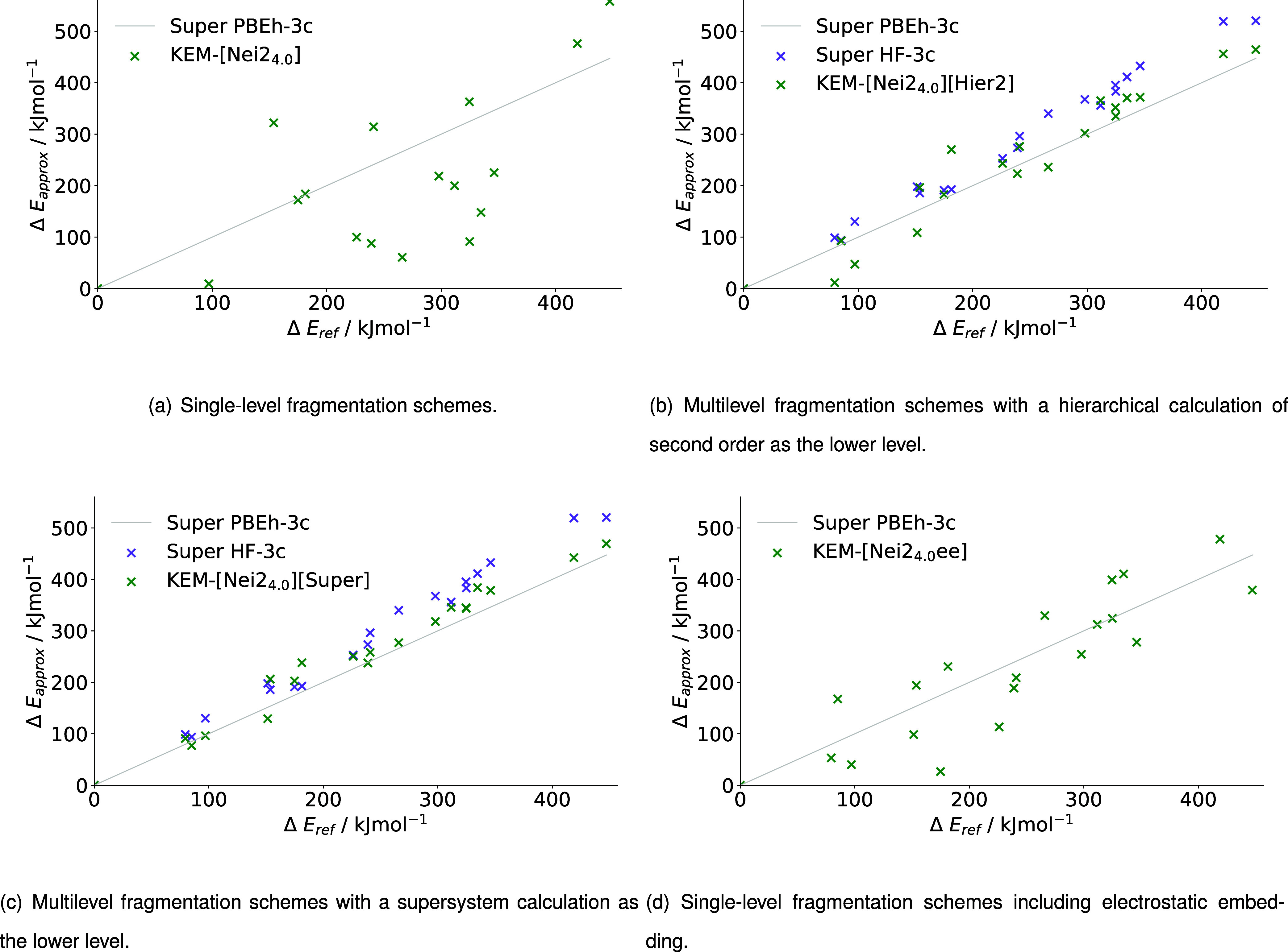
Relative energies of KEM (a) single-level fragmentation schemes, (b) multilevel fragmentation schemes with a hierarchical calculation of second order as the lower level, (c) multilevel fragmentation schemes with a supersystem calculation as the lower level and (d) single-level fragmentation schemes including electrostatic embedding for the relative energies of 20 conformers of 1WN8 calculated with PBEh-3c as higher level and HF-3c as lower level, where applicable.

**Table 7:** MADs and RMSDs of the Relative Energies of 1WN8 Conformers Obtained through KEM-Based Fragmentation Methods

	method	MAD/kJ mol^–1^	RMSD/kJ mol^–1^
SL	KEM-[Nei2_4.0_]	194.57	232.35
ML	KEM-[Nei2_4.0_][Hier2]	27.80	38.12
	KEM-[Nei2_4.0_][Super]	14.98	20.31
EE	KEM-[Nei2_4.0_ee]	64.48	78.85

Opposing observations can be made for KEM-[Nei2_4.0_ee]. The MAD of 64.48 kJ mol^–1^ (c.f. Table 7) already suggests relative energies that do not agree with the supermolecular reference. This can also be observed in Figure 12 (d), where the relative energies of only two conformers between 300 kJ mol^–1^ and 330 kJ mol^–1^ are in close agreement with the supermolecular calculation using PBEh-3c. At a first glance, this result may be surprising, as the electrostatically embedded fragmentation schemes were the most accurate for our implementations of MFHC, pp–GMBE and MIM (c.f. Tables 4–6). However, a possible reason can be found in the choice of initial disjoint fragments. The initial disjoint KEM-[1] fragments are the only ones obtained by severing the peptide bond, which has a partial double bond character. By doing so, interactions are not accounted for. This does not lead to major problems in the ML schemes, as the initial disjoint fragments are recombined and the peptide bond is recovered. However, the KEM-[1] fragments are utilized in the KEM-[Nei2_4.0_ee] scheme for obtaining the needed point charges. Therefore, the severed partial double bond is presumably at fault for the large errors in the EE calculations.

Concluding the results, the SL and ML KEM-[Nei2_4.0_] schemes align with the findings of the MFHC-[Nei*Y*_*d*_], PAIR_2.2_^HB^-[Nei2_4.0_], and MIM-[η = *Y*] schemes. The SL approaches are the most inaccurate out of all fragmentation schemes investigated in this work. The incorporation of a second level of theory improves the relative energies obtained for the conformers of 1WN8, but electrostatically embedded approaches deliver the most reliable results, except for KEM-[Nei2_4.0_ee].

#### Run Times

Although the accuracy of the various fragmentation schemes is undeniably important, their computational cost and feasibility are equally crucial. Therefore, Figure 13 shows run times that are averaged over the 20 investigated conformers of 1WN8. As a reminder, reported run times refer to the sum of the accumulated walltimes of the individual fragment calculations. A KEM-[Nei2_4.0_] FCR, for example, contains about 100 FCs for 1WN8, a MIM-[η = 9] FCR contains about 140 individual combinations, while an MFHC-[Nei2_4.0_] FCR contains about 300 FCs, and a PAIR_2.2_^HB^-[Nei2_4.0_] FCR even contains 1030 individual fragment combinations, which all require individual electronic-structure calculations. Of course, the individual calculations can easily be parallelized.

**Figure 13:**
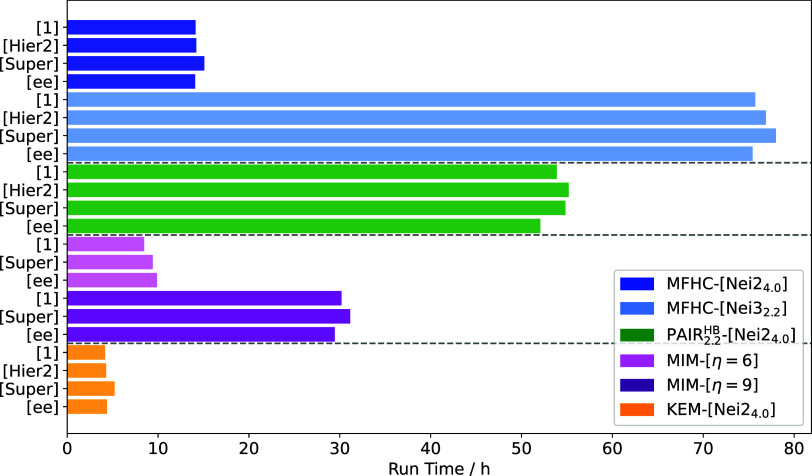
Average run times of different fragmentation schemes for 20 conformers of 1WN8 calculated with PBEh-3c as higher level and HF-3c as lower level, where applicable.

The KEM-[Nei2_4.0_]-based methods obtain results in the lowest amount of time. All of them accumulate only up to 5 h of run time, respectively. Similarly low run times are only achieved with the MIM-[η = 6]-based methods, which can be completed in less than 10 h of run time, respectively. Carrying on with larger initial overlapping fragments in the MIM scheme, the methods with MIM-[η = 9] FCs, of course, accumulate larger run times. Nevertheless, the calculations can still be completed in about 30 h. Similar results can be found for the MFHC-[Nei2_4.0_]- and MFHC-[Nei3_2.2_]-based methods. Beginning with MFHC-[Nei2_4.0_], the fragmentation methods accumulate about 15 h of run time. The larger initial overlapping fragment combinations again lead to a tripling of the run times. When comparing MFHC-[Nei2_4.0_ee] and MFHC-[Nei3_2.2_ee], it can be noted that the computing time for the larger FCs is about five times higher, leading to an accumulated time of about 75 h and therefore the highest computational effort for all investigated fragmentation methods. The run time of the pp–GMBE scheme lies with about 50 h in between the MFHC-[Nei2_4.0_]- and MFHC-[Nei3_2.2_]-based approaches (c.f. Figure 13).

In general, the use of a second level of theory or electrostatic embedding hardly increases the computing time, when compared to the single-level approach in each of the MFHC-[Nei*Y*_*d*_], PAIR_2.2_^HB^-[Nei2_4.0_], MIM-[η = *Y*], and KEM-[Nei2_4.0_] schemes (c.f. Figure 13). This also holds for supermolecular lower-level calculations. This contribution, however, will superlinearly increase with larger system sizes as long as nonlinear lower-level scaling methods are applied. In that case, one may consider much larger fragments for a fragmented lower-level calculation, similar to what is done in the MTA.^26,29^

For our implementations of both the MFHC and MIM schemes we find a significant increase in run time when increasing the coupling limit from MFHC-[Nei2_4.0_] to MFHC-[Nei3_2.2_] and from MIM-[η = 6] to MIM-[η = 9], respectively. While in the MIM case this increase in run time is accompanied by a significant improvement of the results, the MADs for the MFHC cases stay similar (c.f. Tables 4 and 6). In interest of a beneficial cost-accuracy ratio, we, hence, focus on the MFHC-[Nei2_4.0_] and MIM-[η = 9] schemes in the following.

#### Fragment Combination Sizes

To understand the above-discussed difference in run times of the different fragmentation schemes, two factors are of importance: the size and the number of the fragment combinations examined in each method. Figure 14 shows histograms of the number of fragment combinations with a defined number of atoms for the FCRs of the MFHC-[Nei2_4.0_], PAIR_2.2_^HB^-[Nei2_4.0_], MIM-[η = 9], and KEM-[Nei2_4.0_] method for 1WN8, averaged over the 20 conformers. Histograms showing the FC sizes separately for the conformers can be found in Figures S–II to S–V in the SI.

**Figure 14:**
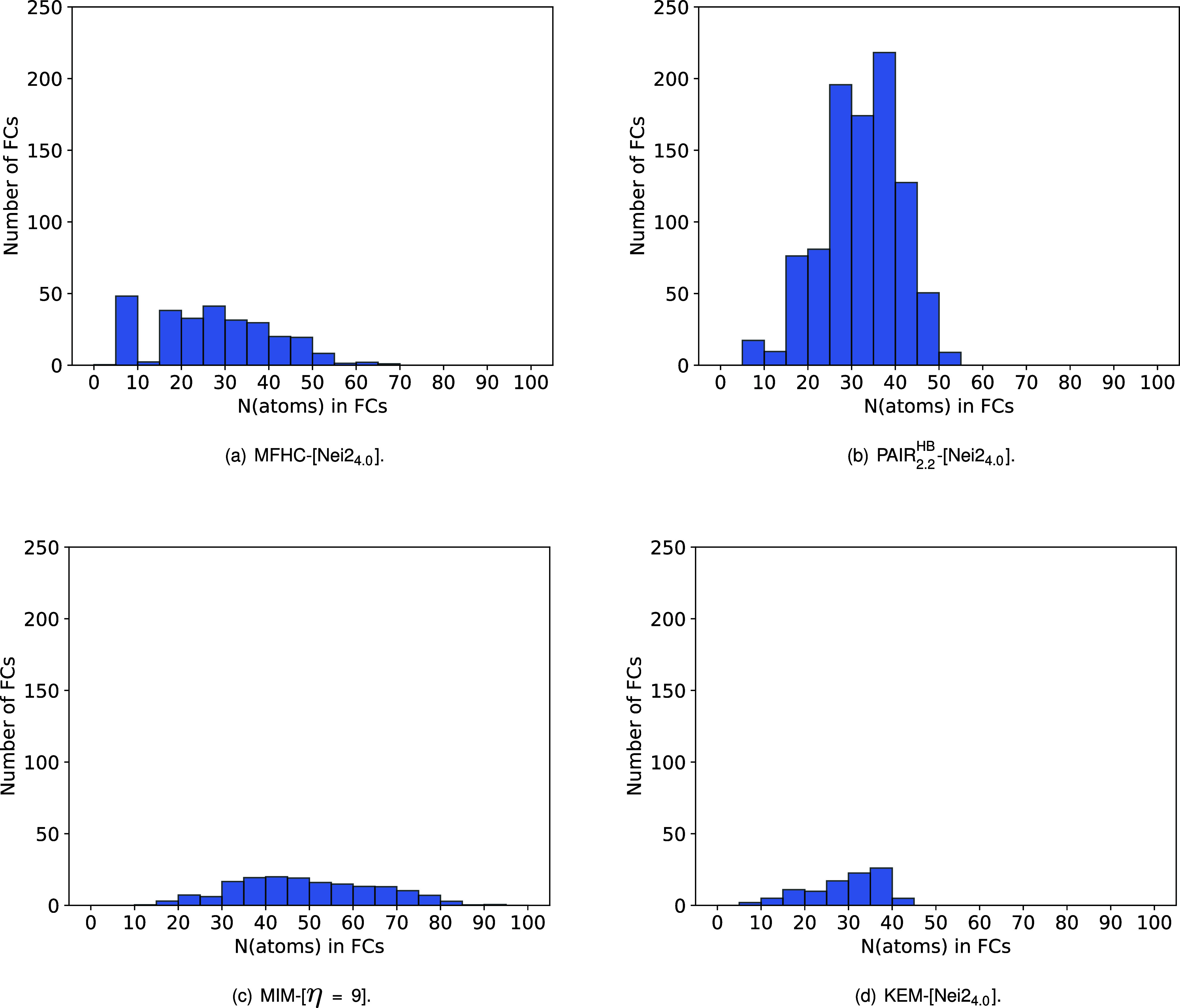
Histograms of the count of fragments with a defined number of atoms for the final overlapping fragments of our implementations of the (a) MFHC, (b) pp–GMBE, (c) MIM, and (d) KEM scheme for the 1WN8 conformers.

Starting with the MFHC scheme, the fragments of MFHC-[Nei2_4.0_] range from a few to 70 atoms (c.f. Figure 14 (a)). The largest share of FCs has a size of five to ten and 15 to 40 atoms. In the PAIR_2.2_^HB^-[Nei2_4.0_] schemes, the results are distinctively different. While the smallest fragments also start at 5 atoms, no fragment exceeds 55 atoms (c.f. Figure 14 (b)), as demanded by the criteria of the pp–GMBE fragmentation scheme described above.^20^ Most of the FCs range from 25 to 45 atoms. The number of FCs in the PAIR_2.2_^HB^-[Nei2_4.0_] scheme is approximately four times greater than in the MFHC-[Nei2_4.0_] scheme. Compared to the MIM-[η = 9] scheme, the PAIR_2.2_^HB^-[Nei2_4.0_] scheme even requires ten times more fragments (c.f. Figure 14 (b), (c)). However, larger fragment combinations can be found for the MIM scheme. The MIM-[η = 9] overlapping fragments start at a size of ten atoms and end with a maximum of 95 atoms. The majority of the fragments range in size from 40 to 70 atoms, which is approximately twice the size of the MFHC-[Nei2_4.0_] fragments. The KEM-[Nei2_4.0_] fragments show an opposing behavior of this fragmentation scheme. All fragments are rather small, ranging from a few to only about 45 atoms (c.f. Figure 14 (d)). Similar to the PAIR_2.2_^HB^-[Nei2_4.0_] fragments, most FCs have a size of 25 to 40 atoms. However, there is only about one-seventh of the number of fragment combinations to be found in the KEM scheme.

These findings match the already discussed run times. Since the majority of the fragments in the MFHC-[Nei2_4.0_] and PAIR_2.2_^HB^-[Nei2_4.0_], as well as the KEM-[Nei2_4.0_] schemes are of similar size, the number of fragments significantly impacts the computing time. With a strongly increasing number of fragment combinations, the accumulated run times of course also increase (c.f. Figure 13). The very small number of FCs in the MIM-[η = 9] scheme (similar to the KEM-[Nei2_4.0_] scheme) should generally correlate with rather small run times. However, the fragment combinations MIM-[η = 9] are as already mentioned noticeably larger than those in the KEM-[Nei2_4.0_], MFHC-[Nei2_4.0_], and PAIR_2.2_^HB^-[Nei2_4.0_] scheme (c.f. Figure 14). While the correlation of the number of fragments to the run time should be linear for fragments of the same size, the increase in run time for larger fragments is, of course, not linear but polynomial, depending on the chosen electronic-structure methods. For this reason, the MIM-[η = 9] schemes accumulate larger run times than the KEM-[Nei2_4.0_] schemes, despite a similar number of fragments (c.f. Figures 13, 14).

### Comparison of Fragmentation Schemes for Different Proteins

The calculations on 1WN8 gave a first insight into the performance of different fragmentation methods. The relative energies as well as run times suggest that the use of multilevel and embedding schemes is of importance, while the transition to larger initial overlapping fragments only leads to a significant improvement in the MIM-[η = *Y*] scheme. While the ML KEM-[Nei2_4.0_] schemes can keep up with the other schemes in terms of accuracy, the electrostatic embedding in particular shows very high MADs (c.f. Table 7). Therefore, we will focus on the fragmentation schemes which provide consistently accurate results over all fragmentation methods and omit further comparisons with our implementation of the KEM scheme. Consequently, MFHC-[Nei2_4.0_], PAIR_2.2_^HB^-[Nei2_4.0_] and MIM-[η = 9] methods will be applied in the following.

#### Performance for Relative Energies

Eleven additional protein systems are studied and compared in addition to the already discussed 1WN8. All of these proteins with the pdb-codes 2RT4, 1EWS, 2LEW, 2GW9, 2LG5, 2M9E, 2NC3, 2KCF, 2KYJ, 1AML, and 5KPH have multiple conformers available in the PDB. Hence, the results can be shown as MADs of the relative energies. For all fragmentation methods and proteins, these can be found in Figure 15. The investigated systems are ordered by increasing size from left to right.

**Figure 15:**
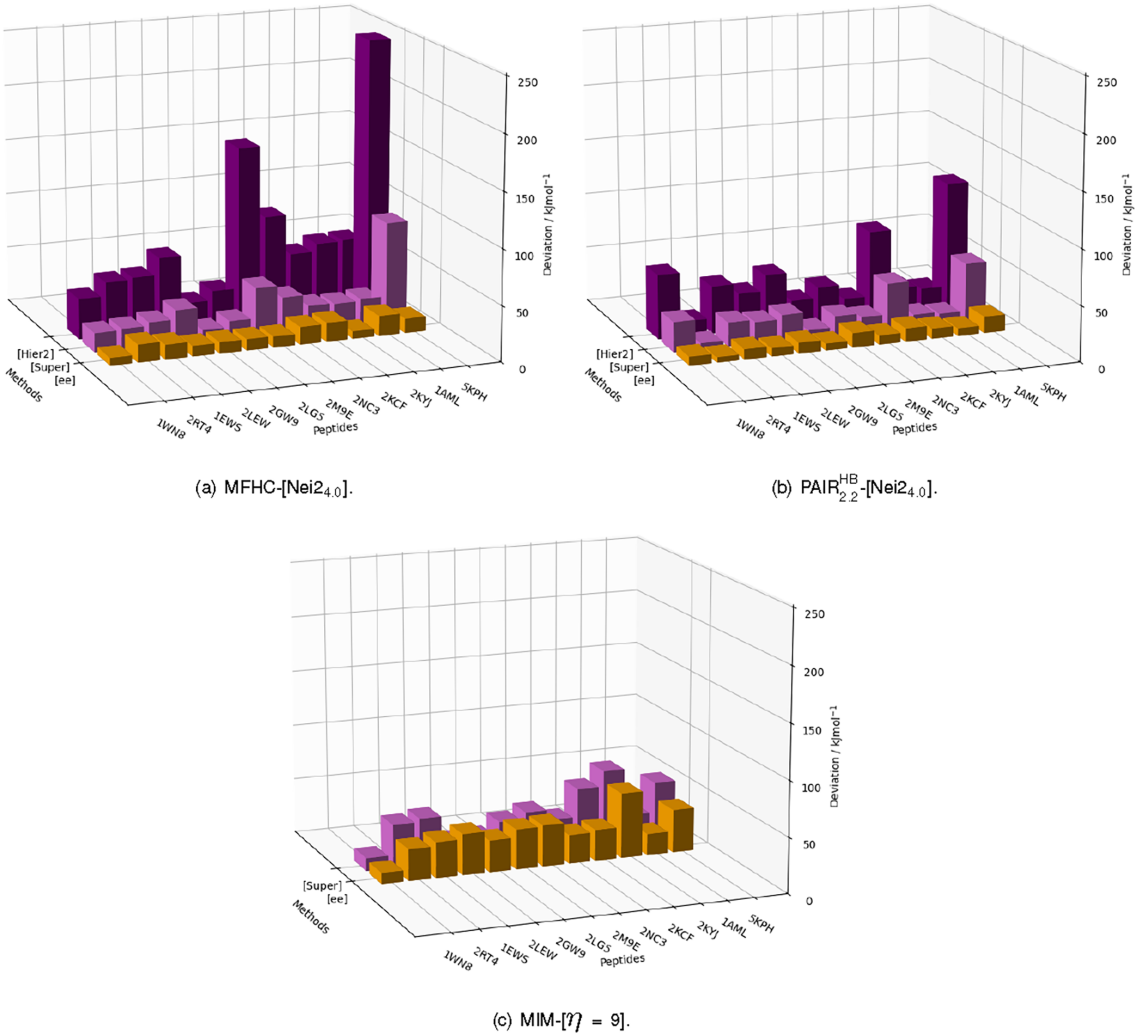
MADs of the relative energies obtained through different fragmentation methods in our implementations of the (a) MFHC, (b) pp–GMBE and (c) MIM scheme for conformers of 12 different proteins calculated with PBEh-3c as higher level and HF-3c as lower level, where applicable.

Starting with results obtained with the MFHC-[Nei2_4.0_] scheme in Figure 15 (a), some interesting observations can be made: None of the methods MFHC-[Nei2_4.0_]­[Hier2], MFHC-[Nei2_4.0_]­[Super] and MFHC-[Nei2_4.0_ee] show MADs significantly increasing with the size of the protein system. This is particularly obvious for the MFHC-[Nei2_4.0_]­[Hier2] method. While 1WN8 and 2RT4 are distinctly smaller than the other systems, the MADs are similar in value to five of the other proteins MADs, namely 1EWS, 2LEW, 2KCF, 2KYJ, and 1AML. The alternating behavior for 2GW9, 2LG5, and 2M9E with respect to system size does not seem to support a correlation between size and error. However, the MAD of the relative energies of the conformers of the largest protein 5KPH shows a large value of nearly 250 kJ mol^–1^. A more detailed discussion on the influence of the system size on the calculations can be found in subsequent sections.

Going on with MFHC-[Nei2_4.0_]­[Super], most MADs are in a similar range: We obtain lower MADs for 2GW9 and 2LG5 and higher MADs for 2M9E and 5KPH. While the MADs show the same trend as the ones obtained for MFHC-[Nei2_4.0_]­[Hier2], all MADs are now distinctly lower than for the hierarchical lower level, with the MAD of 5KPH for example almost being lowered by two-thirds with a value of under 100 kJ mol^–1^ in the MFHC-[Nei2_4.0_]­[Super] scheme. Lastly looking at the electrostatically embedded method MFHC-[Nei2_4.0_ee], all MADs lie below 25 kJ mol^–1^. Especially interesting is the result for the largest protein 5KPH. With an MAD of 12.85 kJ mol^–1^, the calculations even show a higher agreement with the supermolecular reference than those for some of the smaller proteins like 1AML, 2KCF or even 2RT4. Therefore, the inclusion of just the Coulomb interactions of the whole system into the final energies seems to account for the interaction missing from the calculations on fragment combinations reliably. While both MFHC-[Nei2_4.0_]­[Super] and MFHC-[Nei2_4.0_ee] include global interactions, the electrostatic embedding seems to yield better agreement with the supermolecular reference. Therefore, utilizing the higher level of theory for the Coulombic long-range interactions seems to be of greater importance for better agreement to supermolecular results than other nonclassical interaction included in the lower-level supermolecular treatment.

For the PAIR_2.2_^HB^-[Nei2_4.0_] methods in Figure 15 (b), the largest MAD is only slightly larger than 100 kJ mol^–1^. The two ML methods PAIR_2.2_^HB^-[Nei2_4.0_]­[Hier2] and PAIR_2.2_^HB^-[Nei2_4.0_]­[Super] show the same trend in the MADs for the 12 investigated systems, with no clear relation of the MAD to the system size and a higher accuracy with a supermolecular lower level than a hierarchical lower level. The PAIR_2.2_^HB^-[Nei2_4.0_ee] method also mostly resembles the trend shown by the ML approaches, only less pronounced. These observations are very similar to the ones for MFHC-[Nei2_4.0_] methods, especially for the ML methods. They again support that including long-range interactions with a higher level of theory results in a higher accuracy.

Lastly looking at the MIM-[η = 9] methods (c.f. Figure 15 (c)), where only the ML method with a supermolecular lower level and the EE method are compared, MADs of mostly under or about 50 kJ mol^–1^ are achieved, except for the protein 2KYJ with 58.92 kJ mol^–1^ for MIM-[η = 9ee] and 68.84 kJ mol^–1^ for MIM-[η = 9]­[Super]. Interestingly, the ML and EE approaches show very similar performances in the MIM-[η = 9] scheme. There is no general trend which method achieves higher accuracies, for some systems like 2LEW and 2GW9 the ML approach is more accurate, while for example for 2RT4 and 1EWS the EE approach is superior. Nevertheless, the highest accuracy in the MIM-[η = 9] approach is still noticeably lower than the highest accuracies in the PAIR_2.2_^HB^-[Nei2_4.0_ee] and MFHC-[Nei2_4.0_] methods.

For better analysis of the numerical computational scaling of the fragmentation methods with the system size, Figure 16 illustrates the correlation between the number of atoms in the protein systems and the accumulated run times. For the sake of clarity, we here restrict the discussion to the electrostatically embedded versions of the three different fragmentation schemes, MFHC-[Nei2_4.0_ee], PAIR_2.2_^HB^-[Nei2_4.0_ee] and MIM-[η = 9ee].

**Figure 16:**
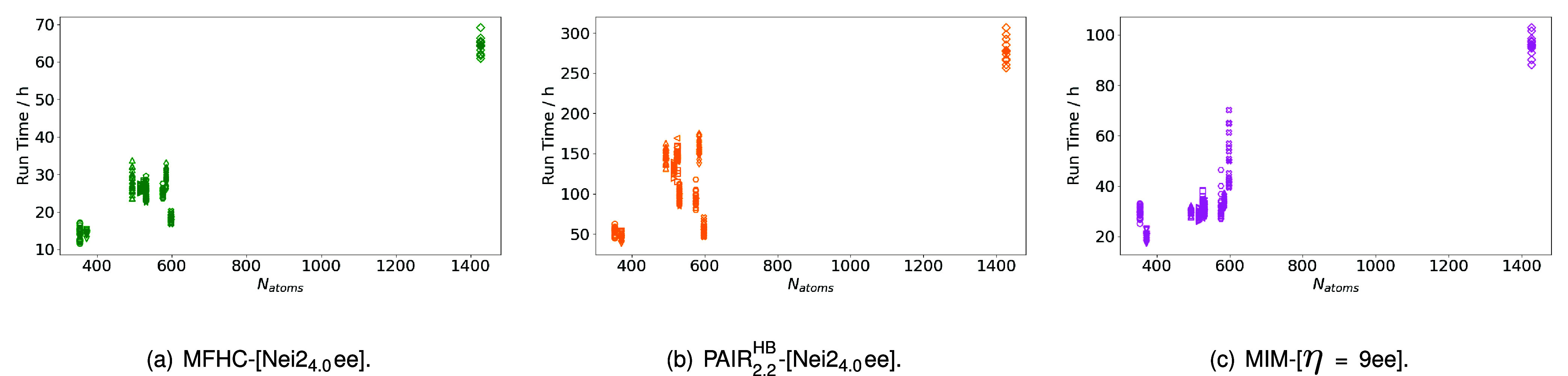
Run times obtained through different fragmentation methods using EE in our implementations of the (a) MFHC, (b) pp–GMBE and (c) MIM scheme for conformers of 12 different proteins calculated with PBEh-3c. Filled markers showcase average run times for all investigated conformers of one protein, while unfilled markers show the run times of the individual conformers. The assignment of markers to proteins can be found in Table S–II in the SI.

The run times of these methods are confirming the insights gained from the 1WN8 calculations (c.f. Figures 13 and 16): For the MFHC-[Nei2_4.0_ee] method, results can, for the smallest proteins 1WN8 and 2RT4 with less than 400 atoms, be obtained in only about 15 h. This value is nearly doubled for the systems with 500 to 600 atoms with an exception being the conformers of protein 1AML, shown as crosses (c.f. Figure 16(a)). For 1AML, the fragment calculations accumulate run times of under 20 h. As expected, the large protein 5KPH accumulates increased computing times of 60 to 70 h. For MFHC-[Nei2_4.0_ee], the accumulated run times for different conformations of the same protein vary by about 5 to 10 h. An exception is the third smallest protein 1EWS, where the different conformers lead to a variation of over 10 h. These are interesting findings, because it shows that the variability in run times does not only depend on the size of the protein, but is also significantly caused by other structural factors, even for the same peptide. One explanation for this is that the numbers and sizes of the fragment combinations differ between the conformers. Because all coupling schemes use distance cut-offs, changes in tertiary structure have an influence on how initial fragments are combined. A higher amount of larger final fragment combinations in conformers can therefore lead to higher run times. Data illustrating this for 1WN8 can be found in Figure S–VI in the SI.

Going on to the PAIR_2.2_^HB^-[Nei2_4.0_ee] method, the run times generally increase with the system size, leading to run times of 250 to 300 h for the conformers of the largest system 5KPH. In comparison to MFHC-[Nei2_4.0_ee], this time scale of the accumulated run times is over four times as high (c.f. Figure 16 (a), (b)). The computing times of the proteins in the 500 to 600 atom region spread out between about 30 and 175 h. 1AML again accumulates the smallest run times with around 50 h, which is nearly the same as for the small proteins 1WN8 and 2RT4. In contrast to that, the protein 2KYJ with a similar atom count accumulates over 150 h of computing time. Interestingly, not only the run times are high for PAIR_2.2_^HB^-[Nei2_4.0_ee], but also the variability of the run times between the conformers of one protein. They differ by about 25 to 50 h for most proteins (c.f. Figure 16 (b)).

Lastly looking at the MIM-[η = 9ee] approach in Figure 16 (c), the computing times again increase with the system size. The run times are generally closer to those accumulated in the MFHC-[Nei2_4.0_ee] method than the ones seen for PAIR_2.2_^HB^-[Nei2_4.0_ee] (c.f. Figure 16), but still somewhat higher. The small proteins 1WN8 and 2RT4 start off with run times of about 30 and 20 h, respectively. The larger proteins with the exception of 1AML accumulate 30 to 40 h of run time and the conformers of 5KPH 90 to 100 h of computing time. Additionally, the run times for most proteins vary by about 10 to 15 h between conformers. As also mentioned for the other methods, 1AML (at 598 atoms) is an outlier. In comparison to the other investigated systems in the same size range, the conformers of 1AML show an average run time of about 50 h and a variability in run times of nearly 40 h. This again shows the influence of structural factors aside from the system size on the run times.

As a general conclusion, we find that MFHC-[Nei2_4.0_ee] obtains results the fastest, closely followed by MIM-[η = 9ee], while PAIR_2.2_^HB^-[Nei2_4.0_ee] accumulates two to four times the run times.

So far, we have discussed run times and accuracy of the methods separately. Figure 17 now depicts the correlation between accuracy and run times for MFHC-[Nei2_4.0_ee], PAIR_2.2_^HB^-[Nei2_4.0_ee] and MIM-[η = 9ee]. The figure nicely summarizes what was already discussed in previous chapters: The MIM-[η = 9ee] scheme generally maintains rather low run times of up to about 50 h for the smaller proteins and 100 h for the large protein 5KPH. However, the deviations range from under 10 kJ mol^–1^ to over 150 kJ mol^–1^. These values are improved when using MFHC-[Nei2_4.0_ee]. The computing times with this scheme are similar or smaller than for MIM-[η = 9ee], while the accuracies are improved to less than 50 kJ mol^–1^ for all investigated systems. Higher accuracies were in this work only achieved with PAIR_2.2_^HB^-[Nei2_4.0_ee], where the majority of results obtained with the fragmentation scheme only deviate from the supermolecular reference by under 25 kJ mol^–1^. While this high accuracy is desirable, the computational feasibility becomes problematic. As already shown in Figure 16 (b), the calculations in the PAIR_2.2_^HB^-[Nei2_4.0_ee] scheme accumulate run times of over 150 h for the proteins with up to 600 atoms and even over 300 h for the larger system 5KPH.

**Figure 17:**
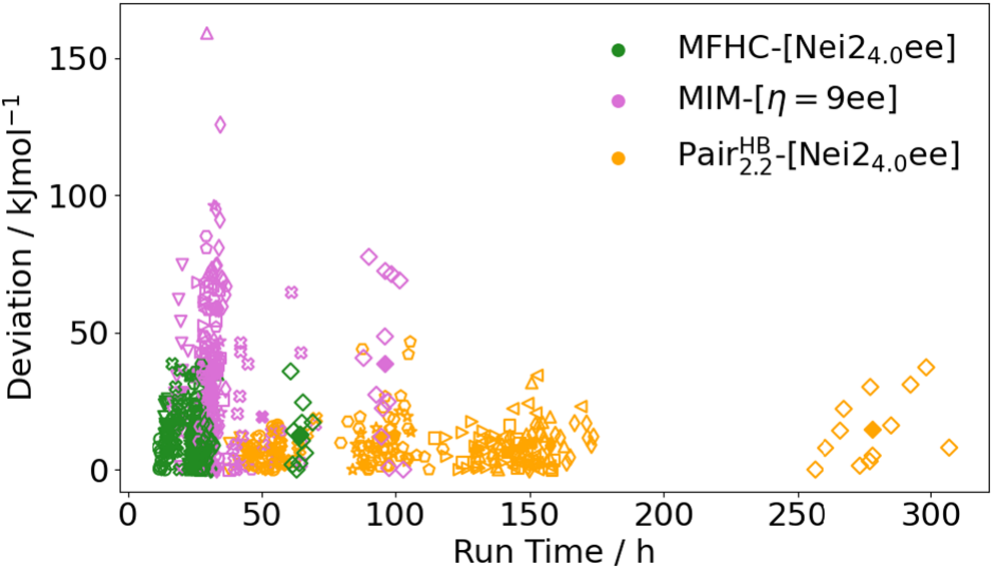
Deviations of the relative energies obtained through different fragmentation methods using EE for conformers of 12 different proteins calculated with PBEh-3c. Different markers denote different protein systems. Filled markers showcase MADs for all investigated conformers of one protein, while unfilled markers show the relative energy deviations of the individual conformers. The assignment of markers to proteins can be found in Table S–II in the SI.

Hence, the utilization of the MIM-[η = 9ee] scheme for relative energies is neither justified by accuracy nor computational cost (c.f. Figures 15, 17). Comparing the MFHC-[Nei2_4.0_ee] with the PAIR_2.2_^HB^-[Nei2_4.0_ee] scheme, both schemes show rather high accuracies, with the PAIR_2.2_^HB^-[Nei2_4.0_ee] scheme being somewhat superior to the MFHC-[Nei2_4.0_ee] scheme in accuracy (c.f. Figure 15). However, the MFHC-[Nei2_4.0_ee] scheme is a minimum of 2.5 times faster, while still achieving reliable relative energies (c.f. Figure 17), which is especially interesting for larger system sizes. Therefore, both the MFHC-[Nei2_4.0_ee] and PAIR_2.2_^HB^-[Nei2_4.0_ee] scheme are good compromises between accuracy and feasibility. It can be decided for each specific application of the FCR framework whether the emphasis should be placed on achieving greater accuracy or more computationally feasible calculations.

#### Performance for Absolute Energies

After having assessed the applicability of the fragmentation schemes for relative energies of 12 proteins, we now extend the test systems to a number of other proteins up to over 2200 atoms. By this, we showcase the applicability to a larger set of proteins and also larger systems. Initially focusing on protein systems with sizes in the same range as before of up to 800 atoms, where we also compare to a supermolecular reference, the calculated absolute energies are presented in Table 8. The supermolecular reference energy is provided as an absolute value, while the energies derived from the fragmentation methods are expressed as deviations from this reference.

**Table 8:** Energies of Various Protein Systems Calculated with PBEh-3c As Well As MADs for the Different Fragmentation Methods^a^

protein	*N*_atoms_	*N*_conf_	energy/kJ mol^–1^								
			MFHC-[Nei2_4.0_]			PAIR_2.2_^HB^-[Nei2_4.0_]			MIM-[η = 9]		Super
			[Hier2]	[Super]	[ee]	[Hier2]	[Super]	[ee]	[Super]	[ee]	
1WN8	354	20	58.91	30.68	6.86	47.50	31.09	11.41	14.72	7.50	–23396193.55
2RT4	372	20	110.08	46.22	14.03	30.47	18.32	5.39	44.73	88.56	–24665115.52
1EWS	494	20	129.03	57.02	9.87	43.16	27.37	15.41	69.29	62.89	–38707313.71
2LEW	516	20	85.93	50.76	7.68	51.76	39.45	8.91	87.65	58.69	–37418574.45
2GW9	524	20	235.06	101.41	10.87	43.58	25.79	9.37	118.66	38.02	–38118535.59
2LG5	526	10	62.72	41.40	17.00	37.79	30.93	6.32	50.08	120.01	–39540903.79
2M9E	531	20	254.06	107.99	16.73	32.15	32.71	19.31	150.45	121.25	–35368825.02
2NC3	531	20	150.56	72.69	14.16	47.08	27.79	10.16	74.69	174.28	–35368309.97
2KCF	576	20	177.22	86.46	11.06	70.52	44.63	14.36	97.25	191.89	–38478311.29
2KYJ	585	20	190.57	89.39	23.69	125.32	65.96	7.30	76.27	153.58	–40520478.67
1AML	598	20	36.61	14.90	17.15	21.19	13.27	7.47	27.86	24.20	–39908261.86
5KPH	1427	12	751.83	298.86	65.08	101.15	89.28	43.79	280.06	360.01	–92130309.65
1VTP	396	1	21.46	7.60	2.31	–17.61	–13.03	–15.31	30.52	–14.41	–26396908.94
1BZG	573	1	–97.59	–40.22	0.06	–1.84	–3.36	–14.04	–0.19	17.96	–36061502.51
2YSC	578	1	–21.71	–24.84	6.60	–21.88	–20.07	–16.43	–5.44	208.44	–38573521.00
2JPK	589	1	80.27	19.53	26.74	–14.97	–6.21	–7.18	60.10	45.53	–36523049.38
2RLK	590	1	140.36	48.08	13.40	150.13	72.99	–1.32	61.11	52.78	–38405213.28
1BHI	591	1	–126.26	–76.33	30.04	–60.79	–49.02	–19.76	–43.63	120.19	–42133015.22
2PPZ	608	1	2181.74	–1013.84	162.69	–26.41	–27.00	–2.95	–46.56	81.51	–39421810.43
3I40	784	1	38.75	–25.52	40.15	–33.90	–44.65	340.01	–26.83	–36.67	–56729488.07
2KIB	824	1	–594.90	–209.78	–16.85	–118.34	–65.82	12.80	–382.31	152.39	–51963002.72
MAD			179.71	80.59	16.85	53.21	35.54	13.85	85.50	106.78	

a Absolute energies for the supermolecular references, deviations from the reference for the fragmentation schemes.

Starting with the MFHC-[Nei2_4.0_] schemes, the fragmentation methods show trends in accuracy similar to those seen for the systems with multiple conformers: Generally, calculations using electrostatic embedding show the smallest deviations from the reference for all proteins, except for 2JPK, 1AML, and 3I40, resulting in a mean absolute deviation of 16.85 kJ mol^–1^ (c.f. Table 8). For the protein 2JPK, the results with electrostatic embedding differ from the reference by 26.74 kJ mol^–1^, whereas the MFHC-[Nei2_4.0_]­[Super] method yields results differing by only 19.53 kJ mol^–1^. For 1AML, the EE results differ from the reference by 17.15 kJ mol^–1^, whereas the MFHC-[Nei2_4.0_]­[Super] results differ by only 14.90 kJ mol^–1^. The case for 3I40 is somewhat different, because both the MFHC-[Nei2_4.0_]­[Hier2] and the MFHC-[Nei2_4.0_]­[Super] method differ from the reference less than MFHC-[Nei2_4.0_ee] (c.f. Table 8). Nevertheless, these differences are rather small and all methods are of a desirable accuracy. The multilevel methods are generally of a lower accuracy in comparison to the EE approach. As expected, multilevel calculations using a hierarchical lower level, which have an MAD of about 180 kJ mol^–1^, are outperformed by the methods utilizing a supermolecular lower level within the MFHC-[Nei2_4.0_] scheme. However, even the largest deviation, found in the 2PPZ structure with 2181.74 kJ mol^–1^ for the MFHC-[Nei2_4.0_]­[Hier2] method, represents merely a 0.01% discrepancy when compared to the full system energy. This is evident when looking at the supermolecular reference, where all absolute energies fall within the 10^7^ kJ mol^–1^ range, making them 7 orders of magnitude greater than the deviations observed in the fragmentation schemes.

Similar remarks can be made for the PAIR_2.2_^HB^-[Nei2_4.0_] schemes: Methods incorporating electrostatic embedding yield the most accurate results, followed by the ML approach with a supermolecular lower level, and then the ML approach with a hierarchical lower level (c.f. Table 8). Again, the absolute energies of some protein systems show interesting exceptions to the trend of the hierarchical lower level being the least accurate, followed by the supermolecular lower level and then the electrostatically embedded calculations. Most noteworthy is the protein 1BZG, where the PAIR_2.2_^HB^-[Nei2_4.0_] approach presents more accurate ML than EE methods (c.f. Table 8) and the use of a hierarchical lower level is even more accurate than a supermolecular lower level. This is assigned to fortunate error cancellation, because it contrasts with the results for all other investigated systems and because the deviations are rather small for all methods discussed. While PAIR_2.2_^HB^-[Nei2_4.0_ee] shows deviations exceeding 10 kJ mol^–1^ for 1BZG, the ML schemes PAIR_2.2_^HB^-[Nei2_4.0_]­[Hier2] and PAIR_2.2_^HB^-[Nei2_4.0_]­[Super] achieve deviations of less than 4 kJ mol^–1^.

For the MIM-[η = 9] methods, the MIM-[η = 9]­[Super] approach is the most accurate with an MAD of 85.50 kJ mol^–1^ for the examined proteins, which is in contrast to the above-discussed cases of MFHC-[Nei2_4.0_] and PAIR_2.2_^HB^-[Nei2_4.0_]. The more accurate ML results can not only be observed for 1BZG as for PAIR_2.2_^HB^-[Nei2_4.0_], but several other proteins, namely 2RT4, 2LG5, 2NC3, 2KCF, 2KYJ, 5KPH, 1BHI, 2PPZ, and 2YSC. All ten proteins demonstrate an unexpected, better performance of the MIM-[η = 9]­[Super] approach in comparison to the MIM-[η = 9ee] approach. Especially noteworthy is 2YSC, where the difference between both methods exceeds 200 kJ mol^–1^ (c.f. Table 8). Despite this seemingly large value, it is important to again note that the deviations of all fragmentation methods from the reference energy are very small in absolute terms, remaining below 0.01%.

Still, even the lowest MAD of 85.50 kJ mol^–1^ for MIM-[η = 9]­[Super] is about five times higher than the lowest MAD in the MFHC-[Nei2_4.0_] approaches, 16.85 kJ mol^–1^ for MFHC-[Nei2_4.0_ee] and nearly seven times higher than the lowest MAD in the PAIR_2.2_^HB^-[Nei2_4.0_] approaches, 13.85 kJ mol^–1^ for PAIR_2.2_^HB^-[Nei2_4.0_ee].

We note that a size-extensive error of interaction energies is generally expected and has also been numerically shown, e.g., for many-body expansions of lower order applied to water clusters^11,80^ However, for water clusters up to 55 water molecules GMBE(2) did not show size-extensive errors in interaction energies.^11^ Since the fragmentation schemes investigated here are somewhat similar in spirit to GMBE(2), we here investigate the size-dependence of the fragmentation error. Figure 18 (a) shows the absolute deviations of all results obtained with MFHC-[Nei2_4.0_ee] against the system size. The data point for 2PPZ in the absolute energies is as an outlier excluded from the depiction for readability reasons. A figure including that data point as well as results for PAIR_2.2_^HB^-[Nei2_4.0_ee] and MIM-[η = 9ee] can be found in figures S–VII and S–VIII the SI.

**Figure 18:**
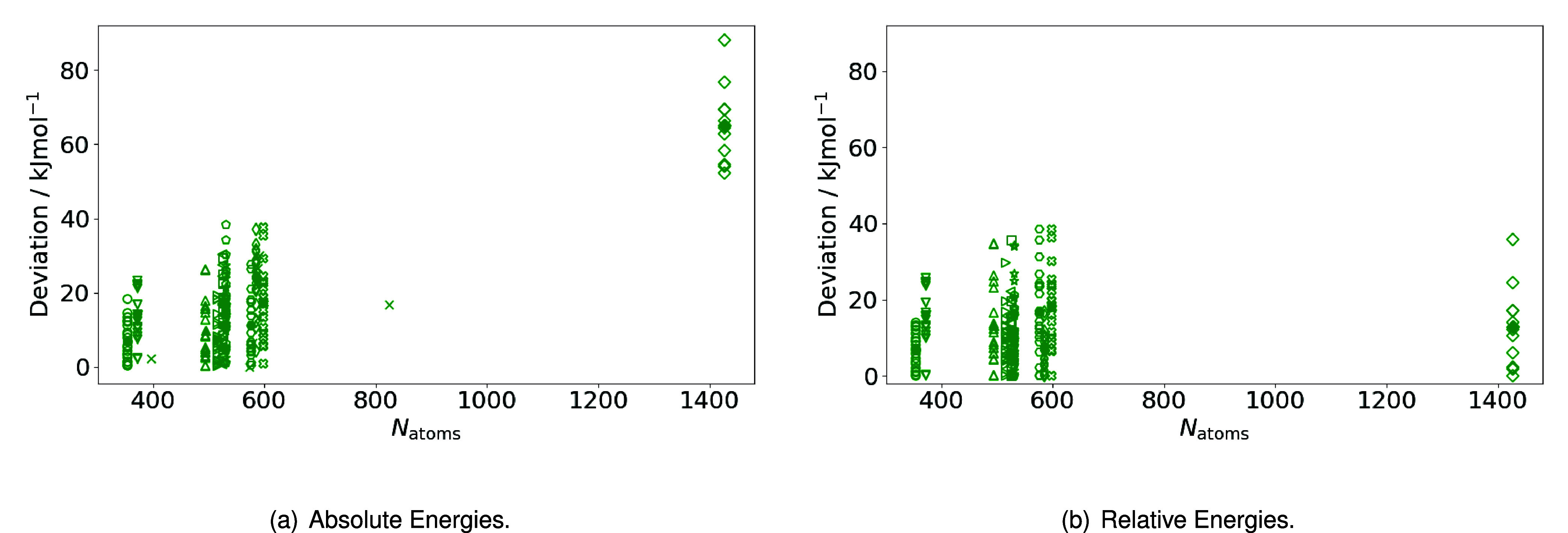
Deviations of the (a) absolute and (b) relative energies obtained through MFHC-[Nei2_4.0_ee] for conformers of different proteins calculated with PBEh-3c as higher level and HF-3c as lower level, where applicable. Different markers denote different protein systems. Filled markers show MADs for all investigated conformers of one protein, while unfilled markers show the energy deviations of the individual conformers. The assignment of markers to proteins can be found in Table S–II in the SI.

Starting with the deviations of the absolute energies in Figure 18 (a), they for no protein or conformer except 5KPH deviate by more than 40 kJ mol^–1^. For all of those proteins with multiple conformers, a deviation close to zero is obtained for at least one of the conformers. However, the deviations vary between the conformers of the same protein. First looking at the smallest proteins 1WN8 and 2RT4, the deviations of the absolute energies vary by only about 20 kJ mol^–1^. The slightly larger protein 2RT4 shows some higher deviations over 20 kJ mol^–1^, while most of 1WN8s conformers show low deviations, which also reflects in a low MAD of 6.86 kJ mol^–1^ (c.f. Figure 18, Table 8). When going on to the systems with about 500 to 600 atoms, a higher variance in deviations of about 40 kJ mol^–1^ can be observed, which is especially noticeable for example for 2M9E, 2KYJ, and 1AML. This increase in variance of the deviations with the system size is not sustained for the largest protein 5KPH with over 1400 atoms. While the large system generally shows higher deviations than the smaller systems of about 45 to 85 kJ mol^–1^, the results for the different conformers similar to the smaller proteins deviate from each other in a range of 40 kJ mol^–1^. Concluding these results for the absolute energies, a slight dependency of the energy deviations on the system size could be assumed. However, the correlation is overshadowed by the influence of other factors that could lead to the variance in deviations between conformers, for example tertiary structure. Hence, we cannot provide a clear statement from the available numerical data.

Since the results in this chapter are shown as absolute energies instead of the relative energies discussed before, it seems sensible to include a comparison of the behavior of absolute and relative energies. Therefore, Figure 18 (b) shows the deviations of the calculated relative energies obtained with MFHC-[Nei2_4.0_ee] against the system size. The only large difference to the absolute energies can be seen for 5KPH. The variance in deviations as well as the value range stays nearly the same for the proteins with up to 600 atoms. While the variance in deviations of 35 to 40 kJ mol^–1^ also stays the same for 5KPH, the value range is now equalized in comparison to the smaller proteins and lays between zero and 40 kJ mol^–1^.

Comparing absolute and relative energy deviations, it seems that the main difference is a possible dependency on the system size for the absolute energies, while relative energies do not show this dependency. However, more proteins of differing sizes would be needed to definitively prove these assumptions. Additionally, the conformers of the proteins show a high variation in energy deviations, which overshadows possible effects of the protein size.

Reasons for differences in MADs of different proteins could be suspected in the structure, like an influence of the secondary or tertiary structure of the proteins. However, an effect of the secondary structure on the accuracy of fragmented calculations could not be proven with our data set, for which reason figures showing the dependency of the MADs on the secondary structure of the systems can be found in figure S-IX in the SI for MFHC-[Nei2_4.0_ee], PAIR_2.2_^HB^-[Nei2_4.0_ee] and MIM-[η = 9ee].

As a next step, we investigated six larger protein systems within the FCR framework. Figure 19 shows the energies of six proteins with system sizes of about 1000 atoms up to over 2200 atoms. The exact values utilized for the figure can be found in Table S–III in the SI. The PAIR_2.2_^HB^-[Nei2_4.0_ee] scheme was chosen as the reference, because the calculations with this scheme have proven to obtain the most accurate energies in the previous chapters.

**Figure 19:**
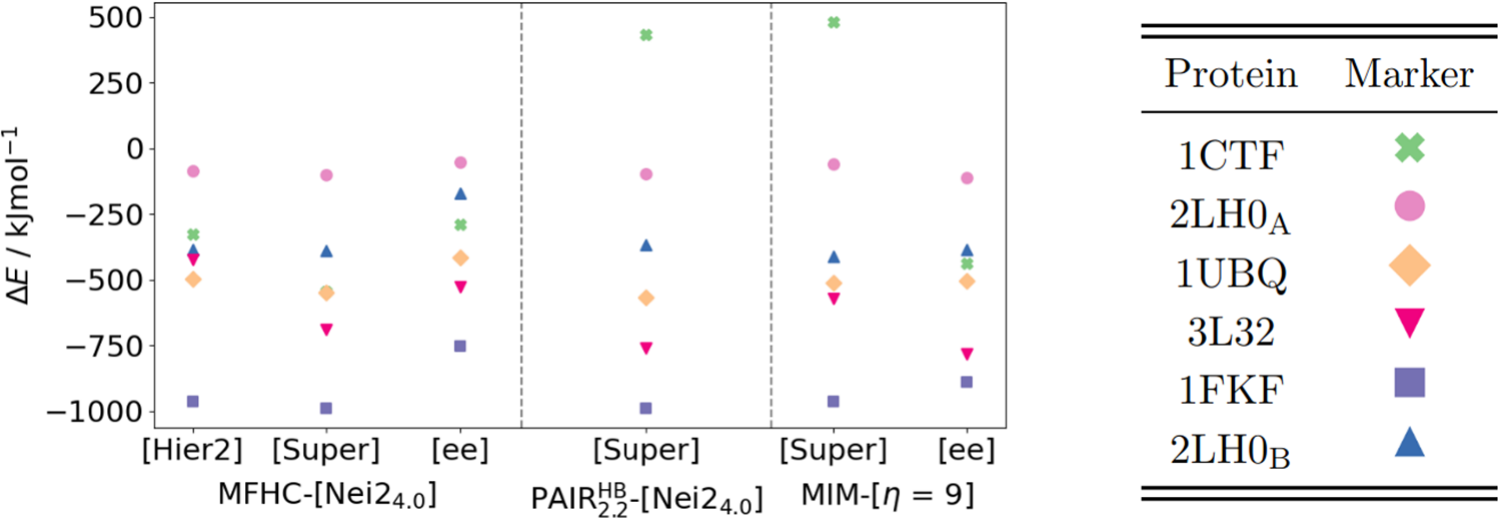
Energies of various protein systems calculated with PBEh-3c. The PAIR_2.2_^HB^-[Nei2_4.0_ee] calculations are used as the reference for each of the three fragmentation schemes.

No PAIR_2.2_^HB^-[Nei2_4.0_]­[Hier2] calculations are included in Figure 19. The reason for this is an unfeasible amount of FCs generated by this method (c.f. Figure 14 (b)). As already mentioned in the case of MIM-[η = 9ee]­[Hier], a very large amount of FCs cannot straight-forwardly and computationally feasibly be managed in the current FCR framework.

First looking at the MFHC-[Nei2_4.0_] schemes, the differences between the energies obtained with the methods and PAIR_2.2_^HB^-[Nei2_4.0_ee] are distributed over a wide range of values from 50 kJ mol^–1^ to 1000 kJ mol^–1^. However, the differences between the respective MFHC-[Nei2_4.0_] methods themselves are rather small. The largest difference in calculated energies can be found for the protein 1CTF with 253.22 kJ mol^–1^ between the setup with a supermolecular lower level and the electrostatic embedding (c.f. Figure 19). Deviations of similar value can be found for two other proteins in both ML approaches, namely 1FKF and chain B of 2LH0. For the other systems, all differences between the ML methods and MFHC-[Nei2_4.0_ee] are lower than 200 kJ mol^–1^, in the case of the MFHC-[Nei2_4.0_]­[Hier2] method for 3I40 even as low as 1.40 kJ mol^–1^.

For the PAIR_2.2_^HB^-[Nei2_4.0_]­[Super] scheme, the deviations from the EE energies are noticeably higher. Only the ML calculation for chain A of 2LH0 leads to a difference of less than 100 kJ mol^–1^ from the EE method, while the highest deviation for 1FKF is of over ten times the value (c.f. Figure 19). The reason for this could be that the PAIR_2.2_^HB^-[Nei2_4.0_ee] calculations lead to absolute energies way lower than the corresponding MFHC-[Nei2_4.0_] and MIM-[η = 9] schemes (c.f. Figure 19). However, to make a decision about which absolute energies are closer to the real absolute energy, a supermolecular reference calculation would be needed as definite proof.

The results for the MIM-[η = 9] schemes look quite similar to the MFHC-[Nei2_4.0_] results. The deviations of MIM-[η = 9ee] and MIM-[η = 9]­[Super] from the PAIR_2.2_^HB^[Nei2_4.0_ee] reference are with values of 60 kJ mol^–1^ to about 1000 kJ mol^–1^ distributed over a large range. Again, however, the MIM-[η = 9] schemes obtain consistent values per protein compared to one another. Most deviations between MIM-[η = 9ee] and MIM-[η = 9]­[Super] are lower than 100 kJ mol^–1^ (c.f. Figure 19). Only the results for 3L32 and 1CTF exceed this threshold. Still, in comparison of the different methods, MFHC-[Nei2_4.0_] schemes obtain results closer to the generally most accurate method PAIR_2.2_^HB^-[Nei2_4.0_ee] than MIM-[η = 9] schemes.

#### Influence of the Electronic Structure Method

To see whether the above findings are specific for the chosen electronic structure method, we repeated the analysis applying B3LYP as higher level, and PBEh-3c as lower level. For this, we chose the same proteins with one conformer as in Table 8 as well as the proteins 1WN8, 1AML, and 2KCF. Due to the increased computational cost of B3LYP reference calculations we are here limited to smaller proteins. Figure 20 exclusively shows the results for the methods using electrostatic embedding, as they were the most accurate when utilizing PBEh-3c as the higher level of electronic-structure theory. Results including the ML schemes can be found in Figures S-X to S-XII in the SI. Especially for 1AML and 2KCF, the relative energies obtained in Figure 20 (b), (c) show a very similar behavior to the relative energies obtained with PBEh-3c (c.f. Figures S-XI and S-XII). Nevertheless, some interesting characteristics can be discussed: For both proteins, the fragmentation methods obtain energies close to the reference with MADs of under 40 kJ mol^–1^. Generally, PAIR_2.2_^HB^-[Nei2_4.0_ee] obtains the most accurate results for both 1AML and 2KCF. In contrast to the PBEh-3c results, the B3LYP results do not show a distinct improvement in the agreement to the reference for MFHC-[Nei2_4.0_ee] results in comparison to MIM-[η = 9ee] results (c.f. Figures 20, S-XI and S-XII).

**Figure 20:**
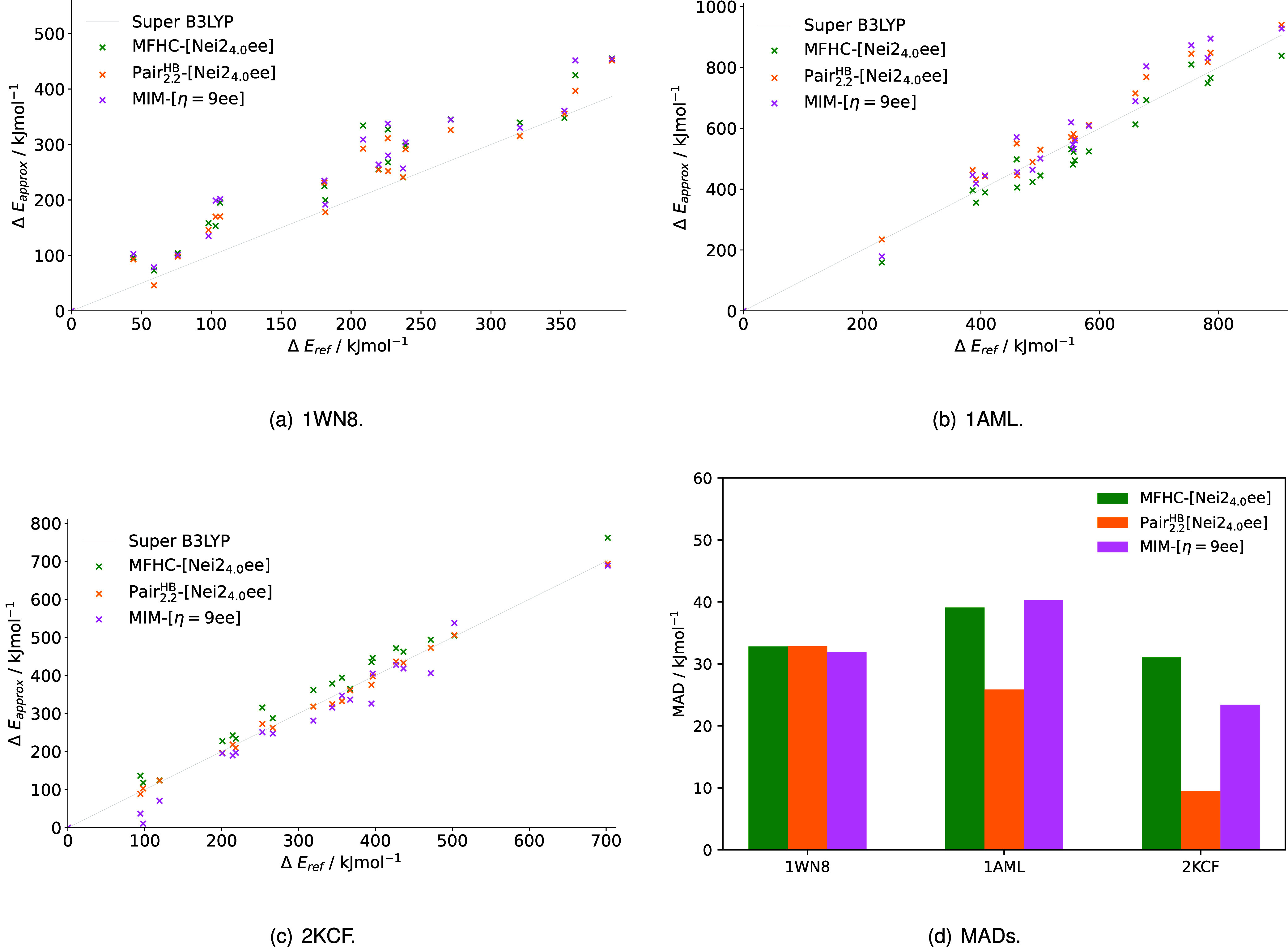
Relative energies of different EE fragmentation schemes for the relative energies of 20 conformers of (a) 1WN8, (b) 1AML and (c) 2KCF calculated with B3LYP/def2-TZVP in comparison to the supermolecular reference (B3LYP/def2-TZVP) as well as (d) respective MADs.

Looking at the results for 1WN8 (c.f. Figure 20 (a)) on the other hand, a rather different behavior from the PBEh-3c analoga can be observed (c.f. Figures 9–11). All fragmentation methods show very similar MADs of about 30 kJ mol^–1^, mostly overestimating the energies. The reason for this is that the relative energies obtained with fragmented B3LYP calculations for 1WN8 do not align with supermolecular B3LYP results but rather with both fragmented and supermolecular PBEh-3c results. This can be seen in Figure S-XIII in the SI, where a version of the B3LYP plots with PBEh-3c as the reference can be found. One reason for this rather unexpected result could possibly be the already mentioned Mulliken charge instability. While the transition from PBEh-3c charges to B3LYP-D3/def2-TZVP charges in this chapter could be assumed as part of the problem, Figure S-XIV in the SI shows that the use of PBEh-3c point charges in the electrostatically embedded B3LYP-D3/def2-TZVP calculations does not lead to a drastic difference in relative energies. Hence, the unexpected deviations from the respective reference calculations in this case can likely not be explained by inconsitencies the point charges used for the electrostatic embedding.

Concluding these results, the fragmented B3LYP calculations on 1AML and 2KCF show similar behaviors as their PBEh-3c counterparts. Therefore, the fragmented calculations could possibly be systematically improved by using higher electronic-structure methods, if a better performance of a more advanced electronic structure method is implied. However, the results for 1WN8 do not support this claim, for which reason a reliable prognosis would require further investigation.

Going on to the proteins with one conformer, the results presented in Table 9 utilize a supermolecular B3LYP calculation as the reference. The energy differences between the various fragmentation methods generally remain within 300 kJ mol^–1^, with exceptions such as 2KIB in the MFHC-[Nei2_4.0_]­[Hier2] scheme, and 3I40 in the MIM-[η = 9ee] scheme (c.f. Table 9). However, the fragmentation methods utilizing a supermolecular lower level seem to mostly obtain results in better agreement with the reference than the ones utilizing electrostatic embedding. The only exceptions to this are the results for 1VTP and 2PPZ in the MFHC-[Nei2_4.0_] as well as PAIR_2.2_^HB^-[Nei2_4.0_] schemes, and 2YSC and 3I40 in the PAIR_2.2_^HB^-[Nei2_4.0_] schemes (c.f. Table 9). This stands in opposition to the results when using PBEh-3c as the higher level of electronic structure theory, where the EE methods mostly obtained the most accurate results in comparison to the reference (c.f. Table 8). The reason for this could possibly be a large improvement in accuracy when using PBEh-3c in comparison to HF-3c for the lower level of theory. Nevertheless, the EE methods when using B3LYP-D3/def2-TZVP are still reasonably accurate with deviations from the reference of under 0.01%. Therefore, when including computational feasibility into the considerations, the EE methods are still of importance, because of their favorable scaling in comparison to a supermolecular lower level. Additionally, consistent trends in comparison to the discussed PBEh-3c results can be found when comparing MFHC-[Nei2_4.0_], PAIR_2.2_^HB^-[Nei2_4.0_] and MIM-[η = 9ee] among each other: The PAIR_2.2_^HB^-[Nei2_4.0_] approaches generally obtain results in good agreement with the reference, closely followed by MFHC-[Nei2_4.0_] schemes. The MIM-[η = 9ee] methods are with some exceptions of a lesser accuracy in comparison to supermolecular results.

**Table 9:** Energies of Various Protein Systems Calculated with B3LYP/def2-TZVP^a^

protein	*N*_atoms_	energy/kJ mol^–1^								
		MFHC-[Nei2_4.0_]			PAIR_2.2_^HB^-[Nei2_4.0_]			MIM-[η = 9]		Super
		[Hier2]	[Super]	[ee]	[Hier2]	[Super]	[ee]	[Super]	[ee]	
1VTP	396	–7.53	–27.28	16.51	27.21	45.62	12.36	–15.89	36.94	–26450487.78
1BZG	573	–50.87	50.40	80.91	88.83	89.87	90.33	39.22	92.53	–36134357.31
2YSC	578	–30.40	18.85	19.14	–22.50	16.24	7.04	80.41	298.64	–38651655.89
2JPK	589	169.37	76.65	135.09	7.24	61.68	91.99	48.49	129.87	–36596723.93
2RLK	590	247.84	95.23	117.80	232.81	109.96	128.71	93.46	196.59	–38482964.55
1BHI	591	–169.06	–17.81	75.27	–59.31	1.95	13.22	43.63	212.12	–42214144.61
2PPZ	608	284.50	–215.71	–65.25	24.36	44.80	27.39	–1.13	118.42	–39499253.12
3I40	784	90.76	45.45	186.80	9.38	44.65	18.16	73.91	450.74	–56838039.74
2KIB	824	–679.98	–8.67	76.17	–87.39	21.46	25.02	67.07	233.35	–52068156.61

a Absolute energies for the supermolecular references, deviations from the reference for the fragmentation schemes.

## Summary, Conclusions, and Outlook

In this work, we presented a comparative benchmark study for fragmentation schemes, namely the molecular fractionation with hydrogen caps (MFHC),^8^ the pair–pair approximation to the generalized many-body expansion (pp–GMBE),^20^ the molecules-in-molecules (MIM)^22^ approach and the kernel energy method (KEM),^25^ using the FCR-based general framework for energy-based fragmentation methods.^8^ We note that our implementations of the different fragmentation schemes are named in compliance with the nomenclature of the FCR framework. Therefore, MFHC-[Nei*Y*_*d*_] is our implementation of the MFHC scheme, which, as opposed to the more widely known MFCC^48^ scheme, includes fragment combinations connected by nonbonded interactions. Our implementation of the original pp–GMBE is PAIR_2.2_^HB^-[Nei2_4.0_ee], while the MIM and KEM approaches are implemented as MIM-[η = *Y*] and KEM-[Nei2_4.0_], respectively. A selection of single-level and multilevel schemes and variants applying an electrostatic embedding scheme within the investigated fragmentation schemes were employed to compare absolute and relative energies, run times and fragment combination sizes for 27 proteins of different sizes.

We have shown for the protein 1WN8 that fragmentation methods using MFHC-[Nei2_4.0_], PAIR_2.2_^HB^-[Nei2_4.0_], and MIM-[η = 9] initial overlapping fragments are a good compromise between accuracy of calculated energies and computational effort, while we refrained from KEM-[Nei2_4.0_] approaches for further calculations in this work. Furthermore, we found that a multilevel approach or electrostatic embedding are of importance for obtaining reliable relative energies. With these findings, we calculated and compared relative energies for 11 more proteins. The comparison of relative energies showed that the use of electrostatic embedding leads to the most accurate results in case of our implementations of MFHC and pp–GMBE, while a multilevel approach with a supermolecular lower level and electrostatic embedding lead to results of comparable accuracy for our implementation of the MIM-approach. For most of the proteins, the smallest mean absolute deviations are found for the PAIR_2.2_^HB^-[Nei2_4.0_ee] method. However, the MFHC-[Nei2_4.0_ee] results are of a similar accuracy and for some proteins, like 1WN8, for example, even show a higher accuracy. Additionally taking run times into account, we find that the pp–GMBE scheme demands far higher computing times than the MFHC and MIM schemes, because of a large number of individual fragment calculations. Nevertheless, a favorable linear scaling of the run time with the system size could be assumed for MFHC-[Nei2_4.0_ee], PAIR_2.2_^HB^-[Nei2_4.0_ee], and MIM-[η = 9ee]. Furthermore, we find that all fragmentation methods utilized in this work lead to similar results for the absolute energies of proteins of various sizes. The deviation in absolute energies between a supermolecular reference and the fragmented calculations is less than 0.1% for all investigated cases, while the deviations in relative energies generally lie within 50 kJ mol^–1^ for pp–GMBE and MFHC. A change in the used electronic structure method appears to shift the supermolecular reference energy and the hierarchy in accuracy between the multilevel and electrostatically embedded fragmentation methods, but still maintains some similar characteristics between the fragmented calculations with both B3LYP/def2-TZVP and PBEh-3c. However, unexpected, larger effects can be seen for 1WN8.

Concluding these remarks, our implementations of the MFHC and pp–GMBE schemes generally achieve more accurate absolute and relative energy calculations compared to the MIM scheme. Considering all evaluations, the MFHC-[Nei2_4.0_ee] method offers the best balance between accuracy in relative energies and computational efficiency for proteins with system sizes up to about 800 atoms. For larger systems and particularly for absolute energies, all fragmentation methods yield comparable results. Thus, multilevel approaches employing a hierarchical lower level or electrostatically embedded calculations can be utilized to keep computing times low.

While we herein already give an overview of 27 different protein systems and different electronic structure methods, additional large proteins and more demanding electronic structure methods could be investigated to scout out the full potential of the FCR framework. Also, additional proteins with a higher variety of protein sizes would be useful to confirm and quantify the correlations of the accuracy with the protein size and the run times with the protein size.

## Supplementary Material



## Data Availability

Data for this article, including xyz-coordinate files for all protein structures and initial disjoint fragments are available at https://doi.org/10.5281/zenodo.19050904.

## Notes

Additional extended figures to display more data points or individual plots alongside mean values, marker definitions, and supplementary comparisons between proteins, as well as data underlying figures in the main text (PDF)
