# Supplementary material for: Comprehensive Comparison of Molecular Fragmentation Schemes for Proteins
Source: J Chem Theory Comput. 2026 Mar 21;22(7):3282–304. doi: 10.1021/acs.jctc.5c01949 (PMC13085253; doi:10.1021/acs.jctc.5c01949)
Supplement: Supplementary file 1 [file ct5c01949_si_001.pdf]

Supporting Information to: Comprehensive Comparison of Molecular Fragmentation Schemes for Proteins

Katharina Rüther, Ken Bunge, Lasse M. Hilmer, Janine Hellmers, and Carolin König*

Institute of Physical Chemistry and Electrochemistry, Leibniz University Hannover, Callinstr. 3A, 30167 Hannover, Germany.

E-mail: carolin.koenig@pci.uni-hannover.de

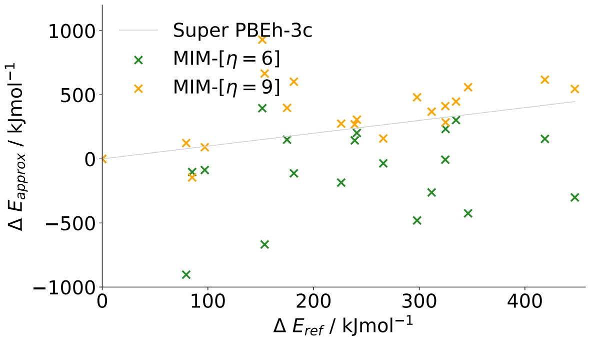

Figure S-I: Relative energies of different single-level MIM fragmentation schemes for the relative energies of 20 conformers of 1WN8 calculated with PBEh-3c.

Table S-I: Data underlying figure 13 in the main text: Average run times of different fragmentation schemes for 20 conformers of 1WN8 calculated with PBEh-3c as higher level and HF-3c as lower level, where applicable.

method	time / h
MFHC-[Nei2$_{4.0}$]	14.13
MFHC-[Nei3$_{2.2}$]	75.73
MFHC-[Nei2$_{4.0}$][Hier2]	14.21
MFHC-[Nei3$_{2.2}$][Hier2]	76.90
MFHC-[Nei2$_{4.0}$][Super]	15.09
MFHC-[Nei3$_{2.2}$][Super]	78.01
MFHC-[Nei2$_{4.0}$ee]	14.08
MFHC-[Nei3$_{2.2}$ee]	75.43
PAIR$^{HB}_{2.2}$-[Nei2$_{4.0}$]	53.89
PAIR$^{HB}_{2.2}$-[Nei2$_{4.0}$][Hier2]	55.20
PAIR$^{HB}_{2.2}$-[Nei2$_{4.0}$][Super]	54.84
PAIR$^{HB}_{2.2}$-[Nei2$_{4.0}$ee]	52.08
MIM-[$\eta = 6$]	8.47
MIM-[$\eta = 9$]	30.19
MIM-[$\eta = 6$][Super]	9.42
MIM-[$\eta = 9$][Super]	31.15
MIM-[$\eta = 6$ee]	9.89
MIM-[$\eta = 9$ee]	29.45

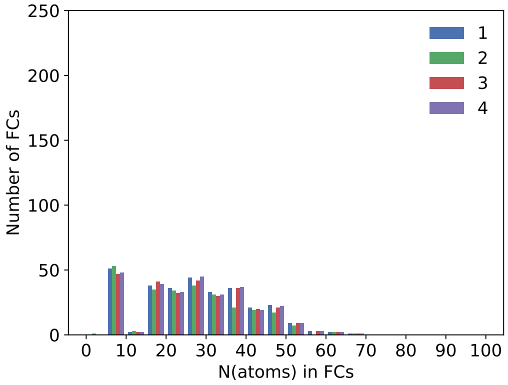

(a) Conformer 1 to 4.

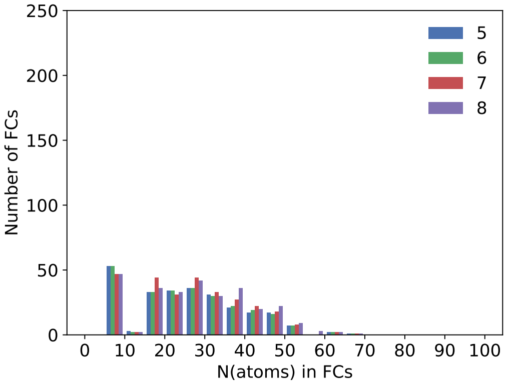

(b) Conformer 5 to 8.

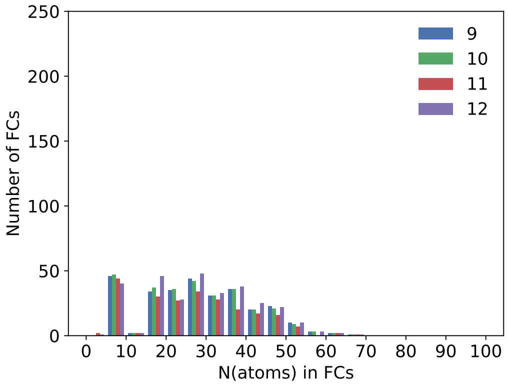

(c) Conformer 9 to 12.

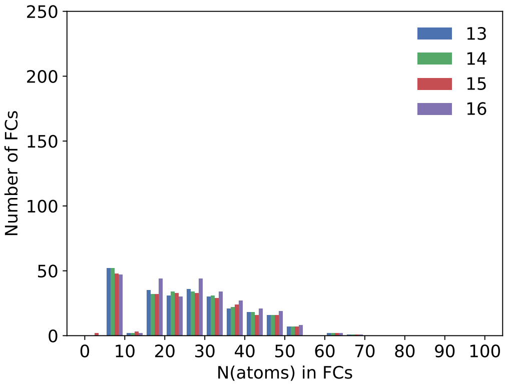

(d) Conformer 13 to 16.

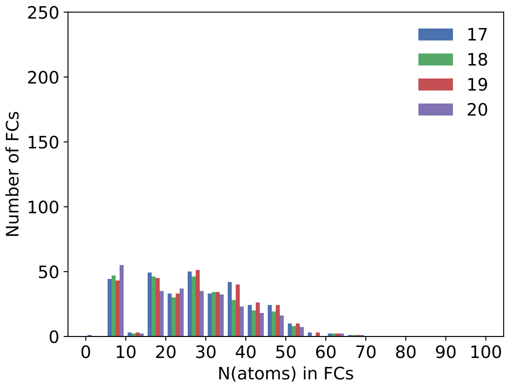

(e) Conformer 17 to 20.

Figure S-II: Histograms of the number of fragment combinations with a defined number of atoms for MFHC-[Nei2$_{4.0}$] calculations for the 1WN8 conformers (a) 1 to 4, (b) 5 to 8, (c) 9 to 12, (d) 13 to 16, and (e) 17 to 20.

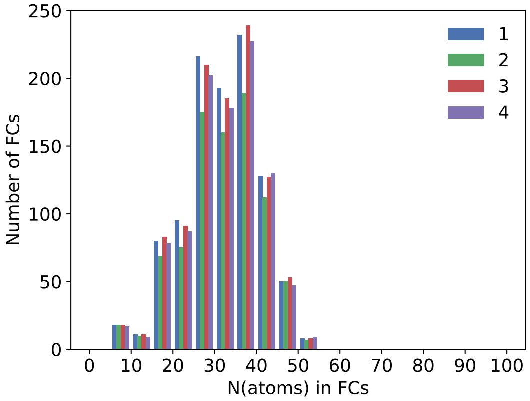

(a) Conformer 1 to 4.

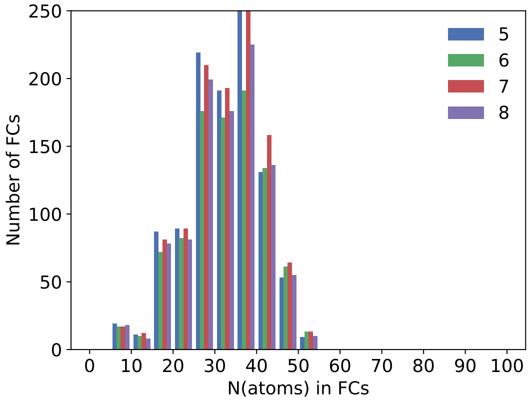

(b) Conformer 5 to 8.

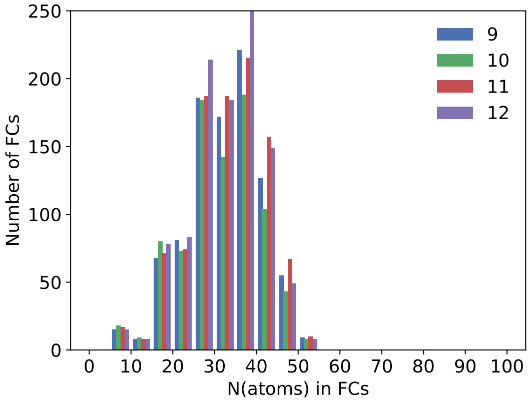

(c) Conformer 9 to 12.

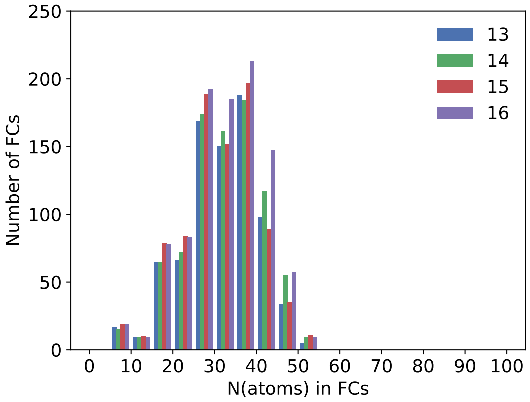

(d) Conformer 13 to 16.

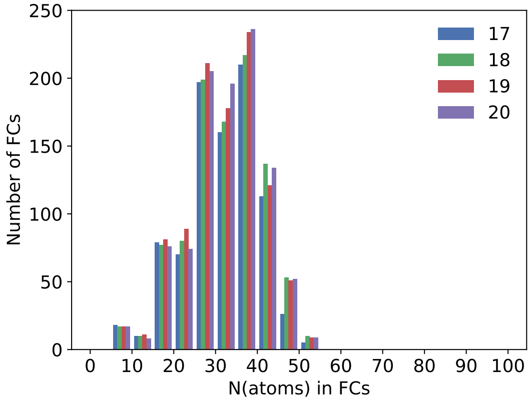

(e) Conformer 17 to 20.

Figure S-III: Histograms of the number of fragment combinations with a defined number of atoms for $\text{PAIR}^{\text{HB}}_{2.2}$-[$\text{Nei2}_{4.0}$] calculations for the 1WN8 conformers (a) 1 to 4, (b) 5 to 8, (c) 9 to 12, (d) 13 to 16, and (e) 17 to 20.

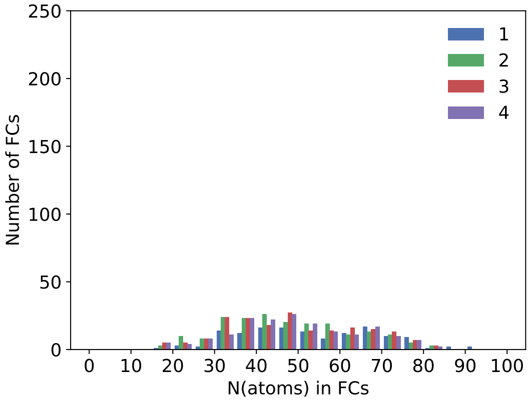

(a) Conformer 1 to 4.

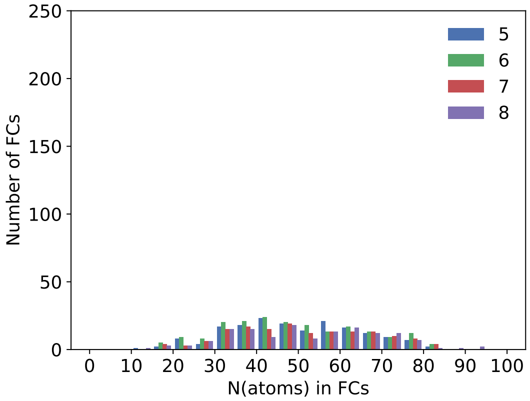

(b) Conformer 5 to 8.

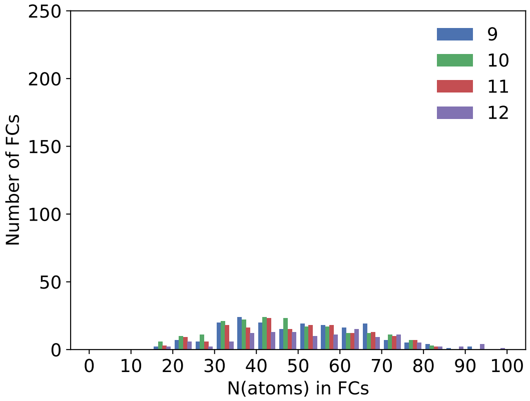

(c) Conformer 9 to 12.

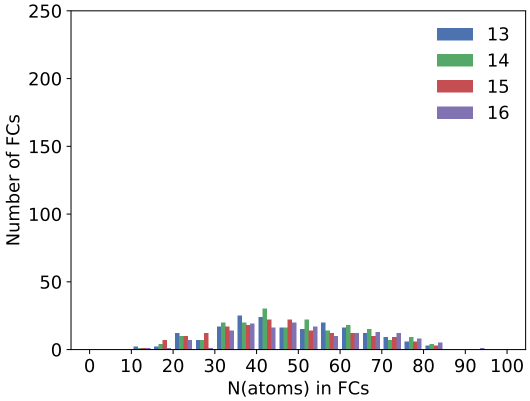

(d) Conformer 13 to 16.

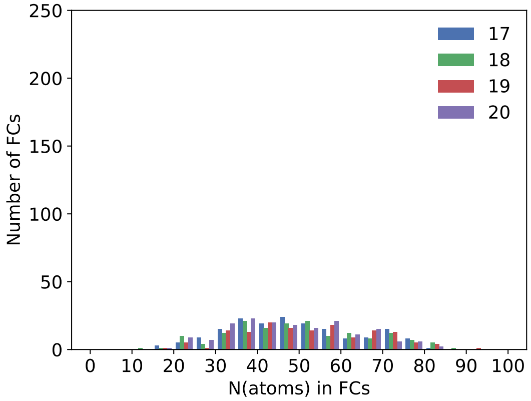

(e) Conformer 17 to 20.

Figure S-IV: Histograms of the number of fragment combinations with a defined number of atoms for MIM-[$\eta = 9$] calculations for the 1WN8 conformers (a) 1 to 4, (b) 5 to 8, (c) 9 to 12, (d) 13 to 16, and (e) 17 to 20.

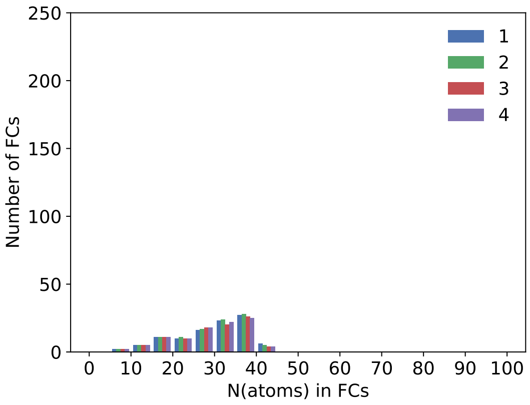

(a) Conformer 1 to 4.

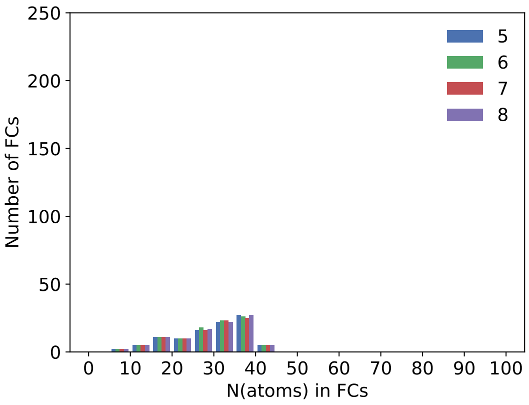

(b) Conformer 5 to 8.

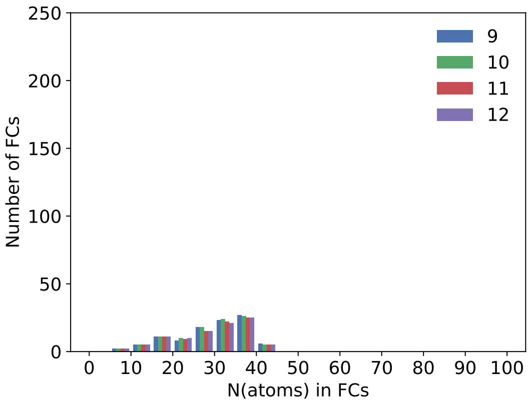

(c) Conformer 9 to 12.

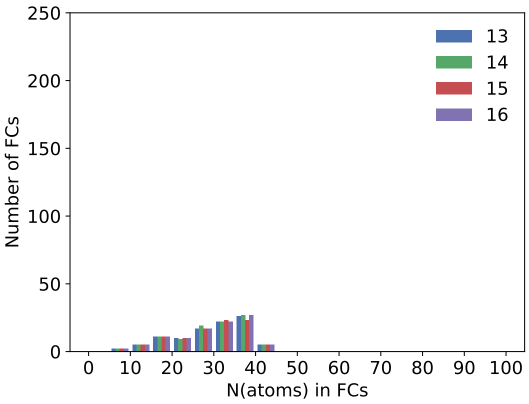

(d) Conformer 13 to 16.

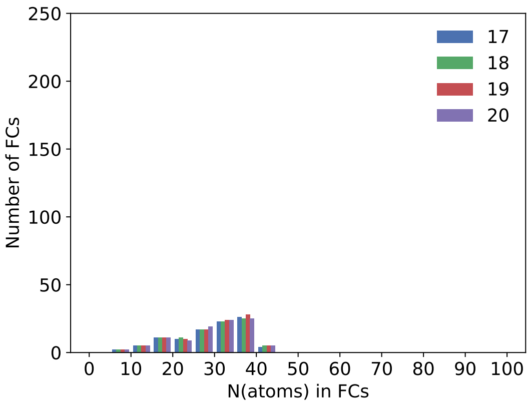

(e) Conformer 17 to 20.

Figure S-V: Histograms of the number of fragment combinations with a defined number of atoms for KEM-[Nei2$_{4.0}$] calculations for the 1WN8 conformers (a) 1 to 4, (b) 5 to 8, (c) 9 to 12, (d) 13 to 16, and (e) 17 to 20.

Table S-II: Markers used for the different proteins.

Protein	Marker
1WN8	●
2RT4	▼
1EWS	▲
2LEW	▶
2GW9	◀
2LG5	■
2M9E	⬟
2NC3	★
2KCF	⬢
2KYJ	♦
1AML	✖
5KPH	◆
Single proteins	×

Table S-III: Energies of various protein systems calculated with PBEh-3c. The PAIR$^{HB}_{2.2}$-[Nei2$_{4.0}$ee] calculations are used as the reference for each of the three fragmentation schemes.

protein	N_{atoms}	Energy / kJ mol^{-1}						
		MFHC-[Nei2$_{4.0}$]			PAIR$^{HB}_{2.2}$-[Nei2$_{4.0}$]		MIM-[$\eta = 9$]	
		[Hier2]	[Super]	[ee]	[Super]	[ee]	[Super]	[ee]
1CTF	1005	-328.09	-544.51	-291.29	431.05	-62641450.31	481.26	-438.76
2LH0$_A$	1139	-85.98	-102.18	-53.29	-95.53	-75108921.54	-60.81	-112.30
1UBQ	1231	-496.60	-549.40	-414.14	-566.65	-78093538.73	-513.52	-504.39
3L32	1493	-423.13	-691.30	-526.14	-761.48	-97071076.53	-570.74	-782.37
1FKF	1662	-964.38	-990.88	-754.80	-988.51	-109594017.5	-963.20	-889.90
2LH0$_B$	2278	-385.37	-390.69	-169.90	-366.09	-150217875	-412.60	-384.40

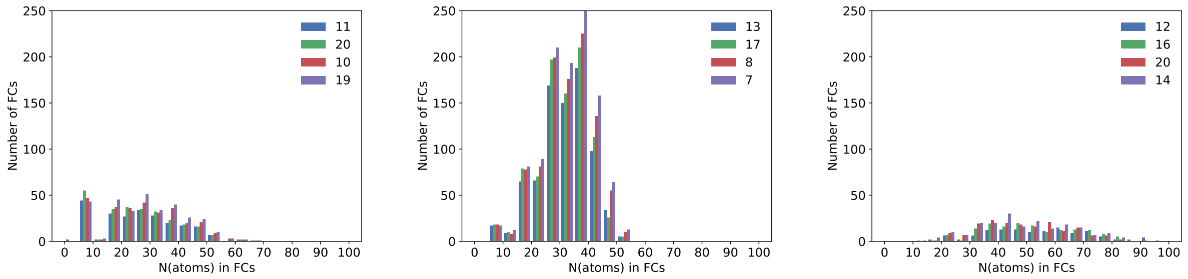

(a) MFHC-[Nei$2_{4.0}$ee]. (b) $\text{PAIR}_{2.2}^{\text{HB}}$-[Nei$2_{4.0}$ee]. (c) MIM-[$\eta = 9$ee].

Figure S-VI: Histograms of the number of fragment combinations with a defined number of atoms for fragmented calculations in our implementations of the (a) MFHC, (b) pp–GMBE, and (c) MIM fragmentation schemes for the 1WN8 conformers. The blue bars represent the respective conformers of 1WN8 that accumulated the lowest run times in the investigated fragmentation schemes. The violet bars represent the conformers that accumulated the highest run times. The green and red bars correspond to conformers with intermediate run times, falling between the observed extremes.

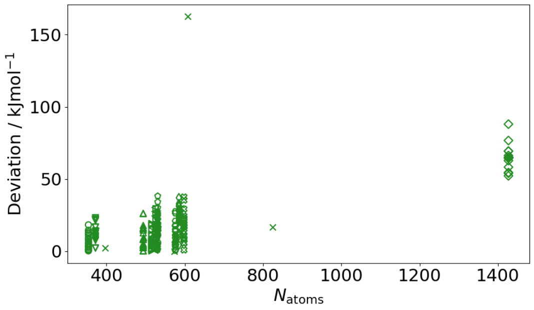

Figure S-VII: Deviations of the energies obtained through MFHC-[Nei$2_{4.0}$ee] for conformers of different proteins calculated with PBEh-3c as higher level and HF-3c as lower level, where applicable. Different markers denote different protein systems. Filled markers showcase MADs for all investigated conformers of one protein, while unfilled markers show the energy deviations of the individual conformers.

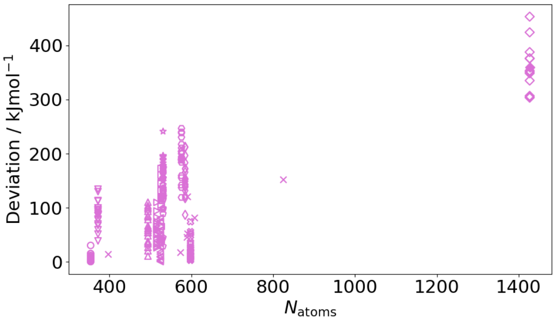

(a) Absolute Energies.

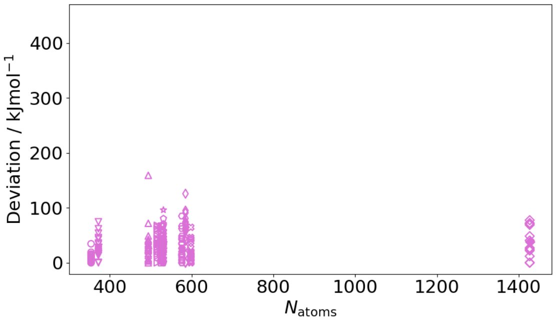

(b) Relative Energies.

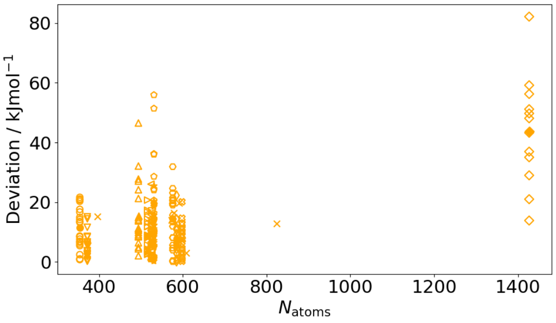

(c) Absolute Energies.

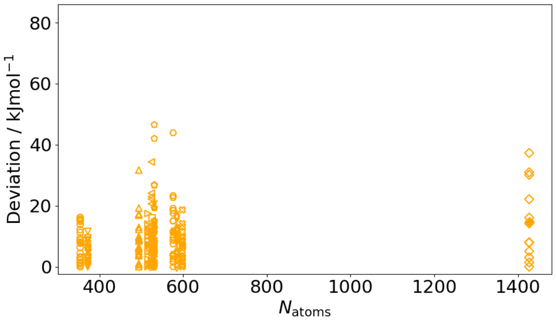

(d) Relative Energies.

Figure S-VIII: Deviations and MADs of absolute (left, (a) and (c)) and relative (right, (b) and (d)) energies of proteins with multiple conformers and those with only one conformer for calculations performed with different methodologies: (a)-(b) using MIM-[$\eta = 9$ee], and (c)-(d) using PAIR$^{\mathrm{HB}}_{2.2}$-[Nei2$_{4.0}$ee].

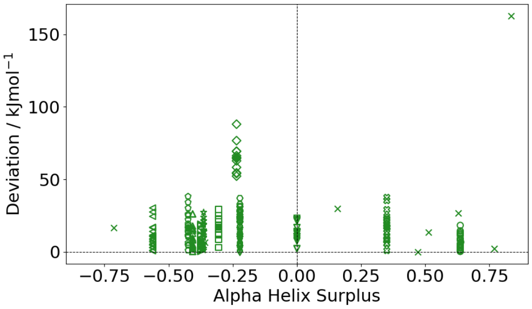

(a) MFHC-[Nei2$_{4.0}$ee].

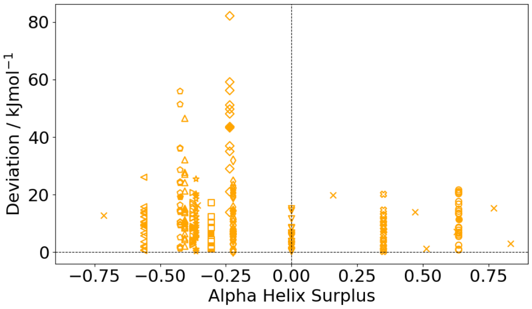

(b) PAIR$^{\mathrm{HB}}_{2.2}$-[Nei2$_{4.0}$ee].

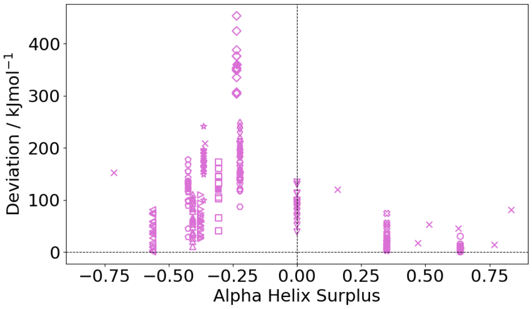

(c) MIM-[$\eta = 9$ee].

Figure S-IX: Deviations and MADs plotted against alpha helix surplus of the proteins for calculations performed with electrostatic embedding in our implementations of the (a) MFHC, (b) pp–GMBE, and (c) MIM scheme.

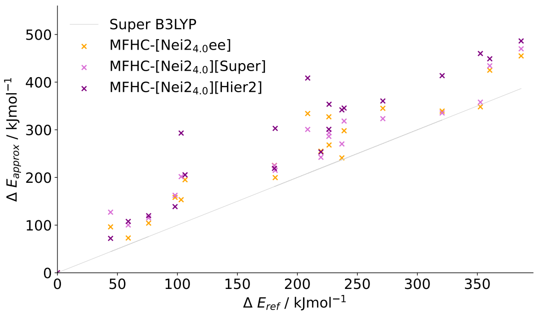

(a) MFHC.

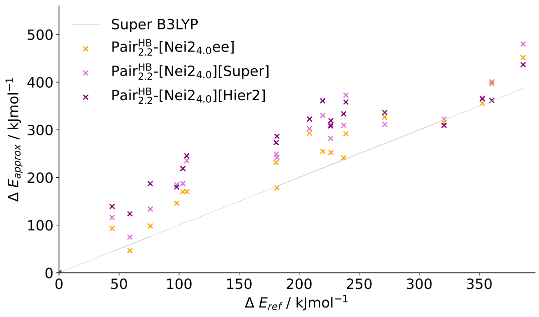

(b) pp-GMBE.

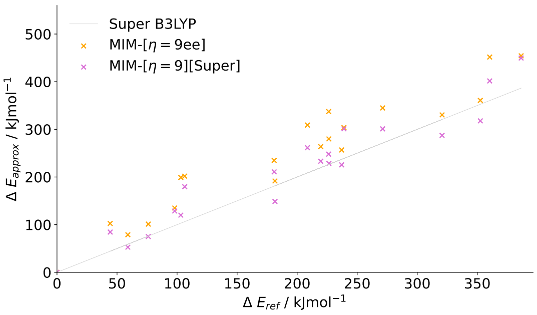

(c) MIM.

Figure S-X: Relative energies of different (a) MFHC, (b) pp–GMBE, and (c) MIM fragmentation schemes for the relative energies of 20 conformers of 1WN8 calculated with B3LYP in comparison to a supermolecular reference (B3LYP).

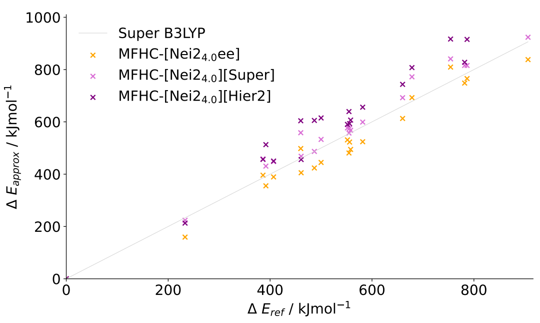

(a) MFHC.

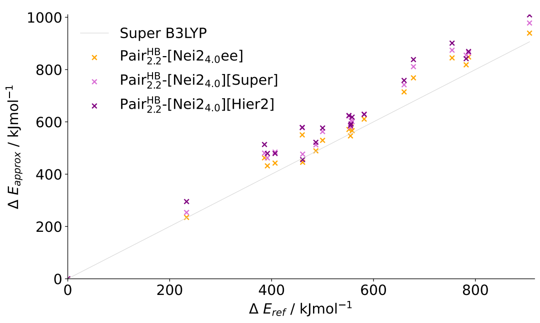

(b) pp-GMBE.

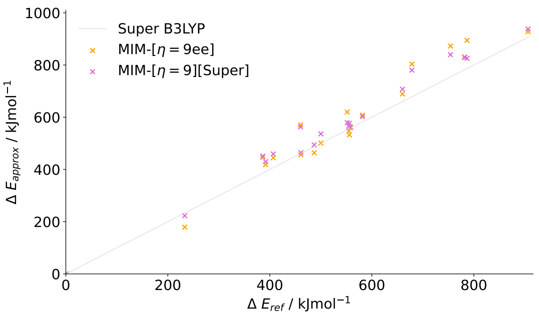

(c) MIM.

Figure S-XI: Relative energies of different (a) MFHC, (b) pp–GMBE, and (c) MIM fragmentation schemes for the relative energies of 20 conformers of 1AML calculated with B3LYP in comparison to a supermolecular reference (B3LYP).

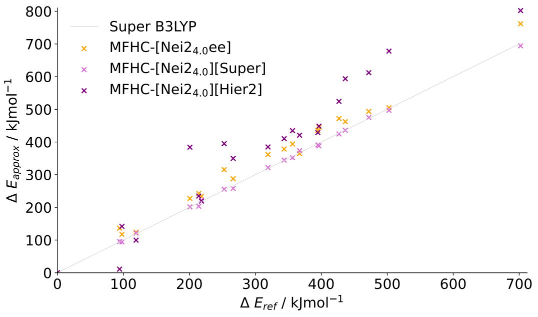

(a) MFHC.

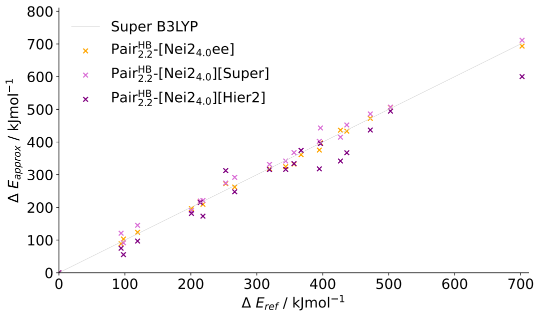

(b) pp-GMBE.

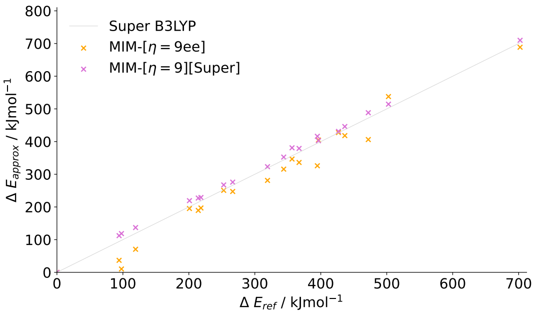

(c) MIM.

Figure S-XII: Relative energies of different (a) MFHC, (b) pp–GMBE, and (c) MIM fragmentation schemes for the relative energies of 20 conformers of 2KCF calculated with B3LYP in comparison to a supermolecular reference (B3LYP).

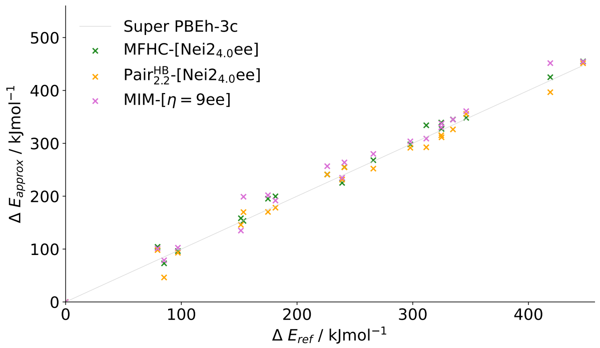

Figure S-XIII: Relative energies of different EE fragmentation schemes for the relative energies of 20 conformers of 1WN8 calculated with B3LYP in comparison to a supermolecular reference (PBEh-3c).

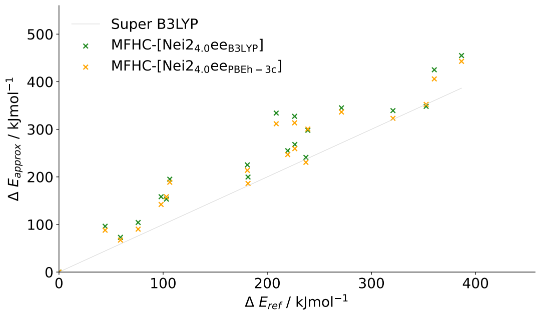

(a) B3LYP reference.

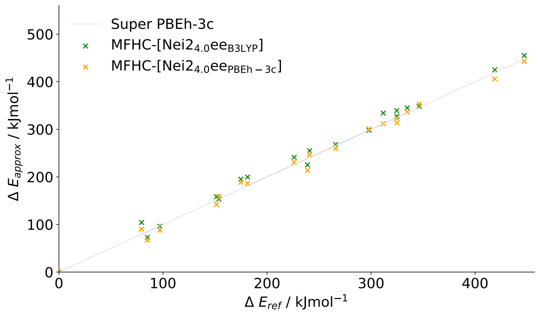

(b) PBEh-3c reference.

Figure S-XIV: Relative energies of different EE fragmentation schemes for the relative energies of 20 conformers of 1WN8 calculated with B3LYP in comparison to a (a) B3LYP or (b) PBEh-3c supermolecular reference. The calculations for obtaining the point charges were either carried out using B3LYP or PBEh-3c, which is shown by the subscript in the nomenclature.

Absolute and Relative Energies

Deviations of Absolute Energies

Table S-IV: Deviations of the absolute energies of 1WN8.

Conf.	Energy Deviations / $kJmol^{-1}$								Abs. Energy / $kJmol^{-1}$
	MFHC-[Nei2$_{4.0}$]			[$\eta = 9$]		PAIR$^{HB}_{2.2}$-[Nei2$_{4.0}$]			Super
	[Hier2]	[Super]	[ee]	[Super]	[ee]	[Hier2]	[Super]	[ee]	PBEh-3c
1	84.34	26.68	7.91	3.56	10.24	1.89	8.55	6.59	-23396349.15
2	94.39	48.12	18.32	12.61	3.29	38.16	26.48	0.80	-23396189.66
3	73.97	33.13	0.80	16.85	5.97	3.74	7.70	20.78	-23396343.52
4	4.36	5.66	0.39	34.73	1.12	15.58	17.30	13.30	-23396247.26
5	111.75	58.78	5.76	63.48	5.24	71.41	44.76	7.65	-23396277.13
6	141.64	63.68	13.40	16.03	10.70	36.94	27.02	6.00	-23396331.58
7	84.42	41.82	3.34	21.85	15.08	62.67	37.72	2.45	-23396428.61
8	22.51	1.63	0.38	10.09	1.20	45.36	36.45	16.72	-23396116.91
9	7.46	15.84	1.48	1.19	11.65	70.97	45.32	21.06	-23396103.97
10	11.88	8.20	8.72	6.96	1.93	29.84	20.25	14.23	-23396202.50
11	87.26	40.40	4.34	31.29	6.87	46.97	25.20	21.64	-23396253.72
12	4.83	7.54	14.52	13.48	30.24	24.78	21.37	8.44	-23396274.90
13	51.39	29.87	1.37	1.59	8.24	40.28	29.48	13.53	-23396093.84
14	73.66	40.48	11.25	8.32	0.56	37.25	27.04	17.61	-23396103.69
15	78.32	45.12	12.18	8.51	0.37	35.71	27.58	8.67	-23396187.65
16	59.31	32.11	2.59	3.25	3.53	74.86	40.08	20.33	-23396130.69
17	21.56	19.07	5.27	10.33	14.84	51.07	34.39	6.91	-23396162.65
18	73.53	43.61	12.47	12.63	4.42	105.59	58.10	5.47	-23395981.40
19	15.42	7.89	3.47	0.36	3.49	56.58	33.19	1.05	-23396082.40
20	76.16	44.03	9.28	17.23	11.05	100.28	53.80	14.92	-23396009.72
MAD	58.91	30.68	6.86	14.72	7.50	47.50	31.09	11.41	

Table S-V: Deviations of the absolute energies of 2RT4.

Conf.	Energy Deviations / kJmol^{-1}								Abs. Energy / kJmol^{-1}
	MFHC-[Nei2$_{4.0}$]			MIM-[$\eta = 9$]		PAIR$^{HB}_{2.2}$-[Nei2$_{4.0}$]			Super
	[Hier2]	[Super]	[ee]	[Super]	[ee]	[Hier2]	[Super]	[ee]	PBEh-3c
1	106.41	50.50	10.82	16.11	99.30	32.65	23.32	6.73	-24665182.53
2	34.08	18.06	9.27	18.10	79.17	19.92	8.97	11.61	-24664972.27
3	60.42	31.17	2.14	58.28	59.25	40.68	22.40	0.27	-24664913.97
4	88.96	33.69	7.48	29.61	129.69	17.62	14.59	5.94	-24665109.25
5	185.38	83.18	22.33	57.45	89.29	20.21	16.83	6.05	-24665280.98
6	81.24	28.85	16.84	8.06	99.70	26.93	20.54	3.80	-24665030.12
7	88.78	39.52	13.10	39.82	96.17	38.04	21.44	1.82	-24665021.97
8	186.80	74.88	12.38	111.35	38.61	12.71	0.48	0.41	-24665359.81
9	58.64	30.21	13.38	29.44	96.10	56.43	29.24	2.93	-24665065.30
10	135.91	47.29	16.78	43.99	95.03	37.17	20.58	3.56	-24665064.01
11	119.08	58.01	10.31	75.87	70.04	50.64	26.19	2.59	-24665213.00
12	119.36	49.71	22.15	39.44	92.49	30.79	18.60	0.77	-24665164.44
13	55.74	25.14	10.50	2.99	134.26	45.02	24.71	15.11	-24665045.18
14	99.30	40.18	23.26	50.70	67.23	26.34	15.42	14.44	-24664969.45
15	179.53	70.18	21.75	119.08	51.27	5.46	8.02	0.33	-24665277.98
16	124.30	48.86	8.25	51.38	77.14	29.02	20.27	8.58	-24665175.44
17	186.61	70.40	22.09	79.15	85.63	11.07	9.87	6.54	-24665350.20
18	98.82	39.99	21.18	29.17	85.40	23.29	16.23	8.33	-24665132.51
19	124.98	55.05	14.18	28.80	112.21	47.57	28.64	2.39	-24665152.65
20	67.27	29.50	2.41	5.81	113.25	37.81	20.08	5.63	-24664829.40
MAD	110.08	46.22	14.03	44.73	88.56	30.47	18.32	5.39	

Table S-VI: Deviations of the absolute energies of 1EWS.

Conf.	Energy Deviations / kJmol^{-1}								Abs. Energy / kJmol^{-1}
	MFHC-[Nei2$_{4.0}$]			MIM-[$\eta = 9$]		PAIR$^{HB}_{2.2}$-[Nei2$_{4.0}$]			Super
	[Hier2]	[Super]	[ee]	[Super]	[ee]	[Hier2]	[Super]	[ee]	PBEh-3c
1	136.76	63.65	5.27	86.28	66.34	1.76	11.70	10.58	-38706815.63
2	243.23	117.94	16.36	155.05	97.74	70.25	53.72	27.07	-38707682.79
3	113.06	50.15	8.43	70.85	61.36	11.44	18.18	14.82	-38706786.31
4	37.94	13.27	15.71	29.00	58.02	11.24	9.89	8.70	-38706938.70
5	108.35	55.36	3.88	106.35	54.25	10.97	28.76	9.15	-38707045.27
6	120.88	42.66	14.67	42.16	100.46	52.95	15.46	4.38	-38707202.99
7	129.84	59.95	4.81	12.18	10.54	57.00	38.81	4.91	-38706940.13
8	177.57	64.24	0.39	20.45	53.72	18.71	22.42	14.15	-38707479.71
9	68.89	33.75	0.24	39.04	83.12	9.58	14.39	32.10	-38707231.81
10	172.95	76.92	8.16	79.94	93.24	26.02	25.36	11.01	-38707213.15
11	167.97	82.46	26.08	48.85	103.82	10.03	10.47	2.07	-38707305.40
12	198.14	58.18	8.59	117.23	26.61	110.44	33.17	24.17	-38706941.30
13	78.15	39.91	3.00	131.77	35.64	153.27	88.61	27.74	-38707290.86
14	87.07	37.26	2.61	24.33	78.14	4.67	7.23	9.86	-38707189.59
15	208.95	92.21	26.32	112.27	47.89	159.15	75.92	21.24	-38707056.86
16	74.88	40.31	2.48	15.49	85.40	20.49	6.40	8.41	-38707076.50
17	37.00	16.82	12.77	44.31	37.75	57.70	17.13	11.25	-38706995.44
18	194.85	94.25	4.17	138.85	20.34	33.15	40.65	46.50	-38707604.89
19	160.27	70.46	17.86	89.22	33.24	20.19	27.67	6.41	-38707452.15
20	63.83	30.56	15.67	22.19	110.15	24.20	1.43	13.74	-38710024.69
MAD	129.03	57.02	9.87	69.29	62.89	43.16	27.37	15.41	

Table S-VII: Deviations of the absolute energies of 2LEW.

Conf.	Energy Deviations / kJmol^{-1}								Abs. Energy / kJmol^{-1}
	MFHC-[Nei2$_{4.0}$]			MIM-[$\eta = 9$]		PAIR$^{HB}_{2.2}$-[Nei2$_{4.0}$]			Super
	[Hier2]	[Super]	[ee]	[Super]	[ee]	[Hier2]	[Super]	[ee]	PBEh-3c
1	61.79	42.32	18.35	81.79	66.40	35.58	41.49	8.35	-37418613.22
2	66.41	43.24	7.05	99.16	32.96	10.06	17.19	4.07	-37418652.32
3	40.00	31.26	14.25	42.27	70.82	16.01	20.71	5.69	-37418673.96
4	70.28	48.22	6.99	82.01	74.47	35.97	33.62	4.33	-37418750.00
5	93.91	56.82	3.38	98.97	51.32	108.16	67.06	11.91	-37418629.25
6	77.57	44.10	19.22	86.11	44.83	24.04	28.42	8.23	-37418661.62
7	111.10	66.04	5.52	102.33	32.23	26.16	31.14	3.79	-37418535.16
8	152.08	80.68	11.33	101.34	94.10	53.42	47.65	3.26	-37418389.10
9	92.87	47.38	18.20	100.20	59.46	26.89	0.12	2.70	-37418464.66
10	63.09	26.21	4.62	79.06	59.50	15.56	5.93	8.43	-37418465.27
11	88.08	44.11	12.00	83.05	41.60	37.08	26.65	17.31	-37418581.76
12	61.03	39.81	1.59	68.92	109.78	119.79	68.25	11.79	-37418458.24
13	80.53	50.20	1.08	124.04	82.01	51.57	39.71	14.36	-37418536.58
14	116.86	70.04	5.27	98.46	60.29	43.43	36.40	2.56	-37418619.87
15	67.64	41.39	7.42	57.32	25.89	138.89	79.80	20.71	-37418524.30
16	147.96	75.60	1.91	95.31	61.36	32.40	35.13	3.57	-37418626.82
17	96.93	56.16	8.25	111.22	58.54	116.14	82.71	16.75	-37418746.85
18	68.47	55.84	6.38	95.59	28.46	38.68	31.24	9.00	-37418566.16
19	70.28	46.90	0.25	27.18	70.21	43.86	49.60	8.13	-37418538.94
20	91.77	48.78	0.63	118.76	49.48	61.57	46.24	13.23	-37418454.95
MAD	85.93	50.76	7.68	87.65	58.69	51.76	39.45	8.91	

Table S-VIII: Deviations of the absolute energies of 2GW9.

Conf.	Energy Deviations / kJmol^{-1}								Abs. Energy / kJmol^{-1}
	MFHC-[Nei2$_{4.0}$]			MIM-[$\eta = 9$]		PAIR$^{HB}_{2.2}$-[Nei2$_{4.0}$]			Super
	[Hier2]	[Super]	[ee]	[Super]	[ee]	[Hier2]	[Super]	[ee]	PBEh-3c
1	219.99	103.01	4.70	126.98	75.26	48.44	31.40	0.79	-38118587.80
2	249.06	98.50	10.89	132.03	18.02	27.06	0.58	12.35	-38118516.56
3	246.72	113.40	27.53	109.36	1.70	161.03	88.88	12.32	-38118559.00
4	244.83	92.69	11.52	134.59	26.74	23.09	2.72	9.27	-38118749.24
5	258.30	105.38	30.32	124.75	58.97	49.64	7.93	9.64	-38118474.48
6	210.38	91.21	9.12	135.34	39.99	34.23	21.93	2.16	-38118393.31
7	241.30	97.22	2.89	120.66	48.93	8.49	12.70	0.61	-38118634.81
8	220.98	99.75	16.70	118.63	14.32	11.49	4.07	8.30	-38118382.40
9	264.62	96.90	8.28	121.57	8.66	50.37	27.54	14.68	-38118434.75
10	206.88	73.18	6.92	89.01	52.13	42.75	3.79	6.66	-38118419.60
11	213.91	101.39	1.12	113.01	10.09	51.16	36.03	13.47	-38118602.65
12	236.09	93.59	7.06	148.54	0.36	4.68	8.32	5.71	-38118455.76
13	247.68	104.49	0.75	93.81	72.44	0.05	14.19	10.41	-38118626.04
14	229.83	95.65	3.10	83.61	57.88	0.69	19.49	10.68	-38118599.23
15	284.63	136.59	24.49	135.09	81.23	22.88	20.01	1.79	-38118588.72
16	190.46	86.30	10.65	168.55	33.86	59.42	39.16	15.81	-38118513.31
17	241.79	112.60	14.08	92.43	3.66	69.56	45.46	26.05	-38118540.57
18	209.55	100.58	4.78	76.09	73.43	73.44	45.77	3.64	-38118403.19
19	211.48	96.35	5.44	88.02	34.70	54.07	33.61	9.15	-38118533.58
20	272.63	129.38	17.02	161.16	48.04	79.09	52.15	13.90	-38118696.85
MAD	235.06	101.41	10.87	118.66	38.02	43.58	25.79	9.37	

Table S-IX: Deviations of the absolute energies of 2LG5.

Conf.	Energy Deviations / $kJmol^{-1}$								Abs. Energy / $kJmol^{-1}$
	MFHC-[Nei$2_{4.0}$]			MIM-[$\eta = 9$]		PAIR$^{HB}_{2.2}$-[Nei$2_{4.0}$]			Super
	[Hier2]	[Super]	[ee]	[Super]	[ee]	[Hier2]	[Super]	[ee]	PBEh-3c
1	83.86	58.40	3.06	77.50	65.29	54.39	37.43	2.11	-39541083.18
2	48.12	30.58	22.70	29.81	105.90	24.62	30.09	1.15	-39540543.56
3	125.54	65.23	24.96	69.57	144.12	35.65	33.79	17.19	-39541039.98
4	19.74	31.25	29.37	7.91	102.13	25.82	29.68	8.59	-39540962.00
5	9.08	16.60	22.29	11.95	40.44	57.29	33.34	4.23	-39540685.97
6	63.64	46.40	15.69	96.17	132.16	43.00	33.71	1.60	-39540969.39
7	32.91	24.37	11.94	43.88	146.04	50.12	31.28	3.81	-39540700.82
8	82.28	49.78	12.91	23.55	160.25	81.94	48.33	12.26	-39541038.34
9	99.02	47.95	8.52	67.85	172.59	1.69	17.67	1.79	-39541087.47
10	62.98	43.44	18.54	72.58	131.19	3.44	13.95	10.44	-39540927.24
MAD	62.72	41.40	17.00	50.08	120.01	37.79	30.93	6.32	-38118602.65

Table S-X: Deviations of the absolute energies of 2M9E.

Conf.	Energy Deviations / $kJmol^{-1}$								Abs. Energy / $kJmol^{-1}$
	MFHC-[Nei$2_{4.0}$]			MIM-[$\eta = 9$]		PAIR$^{HB}_{2.2}$-[Nei$2_{4.0}$]			Super
	[Hier2]	[Super]	[ee]	[Super]	[ee]	[Hier2]	[Super]	[ee]	PBEh-3c
1	163.64	74.83	14.46	79.30	154.74	17.86	12.11	24.05	-35368667.98
2	352.30	151.71	25.08	186.32	98.87	67.60	54.12	36.00	-35368818.43
3	258.58	103.03	10.52	77.83	167.55	25.80	26.01	20.62	-35368965.33
4	293.09	141.31	10.75	177.18	120.79	18.06	16.91	1.88	-35368803.93
5	221.73	115.60	10.82	155.09	131.72	82.27	52.07	24.43	-35368806.34
6	412.99	149.80	22.23	146.51	96.75	1.49	18.24	9.35	-35368607.03
7	271.21	91.42	26.03	222.62	129.60	51.42	16.78	10.39	-35369034.32
8	195.68	89.09	19.34	99.07	136.25	0.39	32.81	10.51	-35368782.14
9	375.22	152.98	30.20	248.94	28.72	42.05	11.96	13.98	-35368957.88
10	276.75	124.62	16.78	200.10	44.80	62.45	52.30	14.56	-35368788.08
11	221.62	100.93	16.03	61.63	155.05	39.03	44.59	51.38	-35368856.05
12	206.29	87.91	6.31	157.57	142.48	25.30	38.19	28.61	-35368763.11
13	122.17	69.07	18.10	135.16	128.53	23.28	36.29	36.21	-35368838.31
14	280.53	126.29	38.30	110.04	114.72	28.03	45.25	5.19	-35368730.81
15	196.55	86.71	13.37	167.32	116.54	21.89	31.21	1.28	-35368886.14
16	262.01	95.79	34.20	136.31	134.77	7.08	31.87	1.43	-35368866.47
17	288.63	118.83	1.08	173.49	132.03	33.87	43.09	9.67	-35368893.40
18	221.66	81.19	4.40	171.35	124.99	27.38	11.58	14.26	-35368714.77
19	277.49	122.00	4.99	131.43	177.34	5.45	28.12	16.40	-35368905.86
20	183.03	76.79	11.51	171.67	88.75	62.37	50.71	55.91	-35368814.14
MAD	254.06	107.99	16.73	150.45	121.25	32.15	32.71	19.31	

Table S-XI: Deviations of the absolute energies of 2NC3.

Conf.	Energy Deviations / $kJmol^{-1}$								Abs. Energy / $kJmol^{-1}$
	MFHC-[Nei$2_{4.0}$]			MIM-[$\eta = 9$]		PAIR$^{HB}_{2.2}$-[Nei$2_{4.0}$]			Super
	[Hier2]	[Super]	[ee]	[Super]	[ee]	[Hier2]	[Super]	[ee]	PBEh-3c
1	81.89	52.62	11.63	81.79	189.53	69.11	36.87	15.64	-35368243.08
2	48.05	37.71	9.70	78.19	182.55	33.41	18.15	19.54	-35368307.50
3	218.11	102.10	14.80	79.34	193.81	46.74	27.36	18.51	-35368333.45
4	252.45	112.23	27.31	110.02	178.34	43.02	42.13	9.07	-35368410.99
5	93.95	56.11	18.76	18.83	241.14	59.85	34.18	12.21	-35368304.95
6	245.34	93.61	10.36	104.22	99.41	25.32	15.46	0.98	-35368499.42
7	91.09	50.12	5.26	35.93	174.77	39.70	22.97	5.58	-35368213.18
8	75.54	46.31	18.78	62.04	162.79	35.60	14.92	1.04	-35368141.08
9	250.33	114.63	23.89	101.61	196.37	3.47	21.07	6.47	-35368294.92
10	67.20	40.92	1.18	10.92	160.86	33.91	13.35	25.44	-35368278.37
11	148.77	69.45	18.56	95.60	154.76	45.48	21.97	7.13	-35368426.76
12	257.64	114.72	21.32	98.71	171.79	94.17	57.88	11.09	-35368355.34
13	124.10	63.96	5.15	76.03	165.06	56.13	29.98	4.97	-35368306.02
14	59.97	40.04	18.88	56.05	163.67	40.45	25.28	20.26	-35368227.25
15	276.72	121.48	21.50	159.72	148.78	21.19	19.42	5.24	-35368455.23
16	222.47	88.92	15.16	62.37	195.54	60.23	38.79	14.10	-35368101.79
17	100.00	57.16	5.61	23.61	170.43	56.69	29.46	0.31	-35368260.33
18	272.72	113.43	13.87	92.58	153.59	72.91	34.40	8.40	-35368426.46
19	66.52	42.91	19.06	70.57	195.03	56.32	30.77	4.93	-35368232.34
20	58.34	35.28	2.46	75.74	187.40	47.92	21.37	12.34	-35368381.02
MAD	150.56	72.69	14.16	74.69	174.28	47.08	27.79	10.16	

Table S-XII: Deviations of the absolute energies of 2KCF.

Conf.	Energy Deviations / $kJmol^{-1}$								Abs. Energy / $kJmol^{-1}$
	MFHC-[Nei$2_{4.0}$]			MIM-[$\eta = 9$]		PAIR$^{HB}_{2.2}$-[Nei$2_{4.0}$]			Super
	[Hier2]	[Super]	[ee]	[Super]	[ee]	[Hier2]	[Super]	[ee]	PBEh-3c
1	260.29	114.85	9.21	186.62	155.36	76.72	44.98	8.15	-38478480.64
2	214.93	107.82	4.42	114.40	137.31	106.72	56.21	14.47	-38478227.28
3	156.71	72.86	11.24	101.59	188.39	72.99	55.19	31.89	-38478277.75
4	65.41	39.64	6.22	42.88	246.66	40.78	36.91	20.49	-38478127.82
5	72.66	56.65	1.19	86.28	204.05	52.21	45.57	19.03	-38478429.78
6	216.47	105.65	4.08	31.21	193.79	31.18	26.53	11.95	-38478417.36
7	202.39	99.96	21.03	164.75	119.00	84.39	61.02	21.53	-38478508.70
8	105.43	52.96	26.36	83.71	210.43	10.54	2.09	20.78	-38478387.57
9	184.11	95.93	6.09	131.56	188.03	64.76	47.83	24.62	-38478259.64
10	141.58	67.84	5.01	48.15	217.19	97.40	56.89	19.50	-38478209.61
11	210.58	102.51	6.77	106.01	189.30	31.08	24.17	4.68	-38478243.42
12	111.98	71.12	13.76	107.61	141.81	79.48	46.02	0.47	-38478123.25
13	183.29	84.77	17.61	72.56	239.99	79.51	48.15	8.10	-38478249.73
14	327.44	142.46	0.48	164.41	159.11	88.31	54.97	3.97	-38478519.99
15	151.16	85.55	17.47	44.28	204.10	147.51	85.90	23.13	-38477929.86
16	178.58	82.68	15.31	139.03	190.83	39.69	22.82	5.62	-38478289.91
17	251.13	109.56	5.89	64.43	200.03	75.81	36.98	15.66	-38478406.85
18	162.72	76.95	3.19	67.41	183.86	83.36	48.77	0.13	-38478339.14
19	94.20	48.57	27.62	41.11	230.34	117.26	59.94	13.38	-38478170.43
20	253.34	110.76	18.16	146.94	238.24	30.75	31.68	19.65	-38478627.16
MAD	177.22	86.46	11.06	97.25	191.89	70.52	44.63	14.36	

Table S-XIII: Deviations of the absolute energies of 2KYJ.

Conf.	Energy Deviations / $kJmol^{-1}$								Abs. Energy / $kJmol^{-1}$
	MFHC-[Nei2$_{4.0}$]			MIM-[$\eta = 9$]		PAIR$^{HB}_{2.2}$-[Nei2$_{4.0}$]			Super
	[Hier2]	[Super]	[ee]	[Super]	[ee]	[Hier2]	[Super]	[ee]	PBEh-3c
1	140.51	74.02	23.79	54.76	131.67	128.93	63.59	6.38	-40520403.59
2	350.76	140.34	4.35	204.11	121.48	115.92	59.84	14.39	-40520836.41
3	209.68	92.05	24.41	37.99	153.81	124.27	61.83	10.70	-40520403.91
4	172.86	90.01	16.84	109.15	138.37	128.37	72.24	7.24	-40520553.40
5	135.38	67.04	27.88	52.61	117.57	184.94	89.87	6.14	-40520267.55
6	216.91	95.81	28.89	16.52	171.55	131.77	66.99	0.92	-40520262.37
7	126.18	70.17	21.17	7.43	212.50	124.07	61.99	5.39	-40520176.55
8	191.84	87.98	31.56	84.79	142.83	83.42	47.67	6.15	-40520608.44
9	176.97	94.13	22.59	141.23	138.09	145.20	80.61	11.45	-40520731.10
10	194.92	93.90	28.61	128.13	143.67	148.96	85.48	7.31	-40520529.33
11	173.22	89.79	10.75	146.06	139.66	177.27	83.78	7.90	-40520536.94
12	241.88	105.95	32.95	79.97	172.50	145.39	70.48	22.30	-40520559.75
13	256.83	103.86	25.80	68.10	153.08	33.90	30.61	4.32	-40520561.04
14	122.82	61.74	37.06	9.37	211.64	130.70	63.09	7.25	-40520253.47
15	172.17	85.47	20.62	106.79	148.84	122.02	65.89	6.21	-40520567.48
16	172.00	84.26	24.27	81.04	165.67	110.65	65.14	2.75	-40520369.99
17	221.09	99.25	27.82	32.97	183.22	89.31	52.40	0.17	-40520450.63
18	208.30	95.46	20.96	32.29	86.80	96.79	59.74	2.95	-40520514.58
19	159.05	75.33	31.04	56.20	142.07	168.12	79.92	9.37	-40520422.83
20	168.14	81.21	12.34	75.88	196.53	116.40	58.15	6.62	-40520564.11
MAD	190.57	89.39	23.69	76.27	153.58	125.32	65.96	7.30	

Table S-XIV: Deviations of the absolute energies of 1AML.

Conf.	Energy Deviations / $kJmol^{-1}$								Abs. Energy / $kJmol^{-1}$
	MFHC-[Nei2$_{4.0}$]			MIM-[$\eta = 9$]		PAIR$^{HB}_{2.2}$-[Nei2$_{4.0}$]			Super
	[Hier2]	[Super]	[ee]	[Super]	[ee]	[Hier2]	[Super]	[ee]	PBEh-3c
1	26.55	28.44	35.35	49.84	18.42	33.88	20.53	14.67	-39908802.23
2	82.80	21.90	17.71	36.51	4.13	22.14	14.90	4.53	-39908315.86
3	55.36	15.61	17.12	45.50	16.88	6.67	0.39	8.75	-39908287.14
4	49.68	13.93	5.55	13.63	10.84	17.05	11.57	3.10	-39908308.39
5	83.46	23.31	0.90	35.36	9.49	0.49	5.86	1.50	-39907891.34
6	18.74	0.08	8.06	25.31	7.03	26.17	9.76	0.90	-39908242.46
7	39.38	23.40	21.44	11.66	23.62	43.81	18.88	10.24	-39908351.24
8	66.52	16.93	18.90	28.69	47.98	26.49	20.62	1.75	-39908015.92
9	0.90	9.29	37.62	9.19	8.22	36.97	15.09	12.64	-39908260.79
10	22.60	3.96	9.04	20.48	2.74	9.57	8.77	8.84	-39908149.33
11	23.22	15.05	10.63	12.20	6.37	50.82	27.82	0.24	-39908029.38
12	17.27	16.93	24.44	0.86	74.09	34.70	27.12	1.76	-39908240.66
13	58.97	25.33	29.21	62.71	55.68	10.17	8.78	4.60	-39908001.16
14	21.74	4.22	18.74	36.84	25.91	5.16	8.93	6.69	-39908400.82
15	12.52	2.60	8.10	2.30	11.76	17.55	11.39	11.29	-39908264.81
16	10.81	1.53	13.38	24.48	52.05	2.52	5.14	4.00	-39908056.42
17	32.02	27.31	5.83	63.20	33.43	1.55	9.68	20.13	-39908572.78
18	29.90	23.19	23.11	4.76	18.65	41.74	22.24	11.05	-39908363.10
19	56.80	20.61	15.35	41.61	20.53	5.63	5.39	12.87	-39908410.71
20	23.00	4.44	22.56	32.16	36.20	30.77	12.47	9.75	-39908272.73
MAD	36.61	14.90	17.15	27.86	24.20	21.19	13.27	7.47	

Table S-XV: Deviations of the absolute energies of 5KPH.

	Energy Deviations / $kJmol^{-1}$								Abs. Energy / $kJmol^{-1}$
Conf.	MFHC-[$Nei2_{4.0}$]			MIM-[$\eta = 9$]		$PAIR^{HB}_{2.2}$-[$Nei2_{4.0}$]			Super
	[Hier2]	[Super]	[ee]	[Super]	[ee]	[Hier2]	[Super]	[ee]	PBEh-3c
2	873.92	341.48	62.78	378.91	306.82	68.78	65.16	59.14	-92130771.08
4	836.21	286.93	88.03	191.53	335.07	50.93	19.34	56.22	-92129978.97
5	835.52	337.52	54.04	245.67	376.31	123.17	104.30	49.71	-92130491.73
6	844.91	337.96	58.28	435.45	303.37	11.24	59.36	36.93	-92130714.93
8	736.52	289.68	54.52	319.32	304.54	86.30	78.00	35.04	-92130144.25
9	898.19	360.38	64.41	213.83	453.29	81.21	71.20	29.00	-92130495.25
10	735.61	281.96	65.00	245.46	351.02	160.59	138.63	21.01	-92130099.99
12	696.02	276.78	76.70	309.80	348.49	144.09	118.40	82.10	-92130148.80
13	753.96	344.83	66.28	262.10	424.27	158.00	106.40	43.24	-92130057.69
14	744.67	296.83	69.32	278.06	387.75	75.80	95.30	48.08	-92130568.66
17	561.15	222.54	69.40	241.84	353.42	45.65	73.95	13.87	-92130369.73
20	505.30	209.48	52.24	238.79	375.76	208.08	141.37	51.13	-92129874.74
MAD	751.83	298.86	65.08	280.06	360.01	101.15	89.28	43.79	

Deviations of Relative Energies

Table S-XVI: Deviations of the relative energies of 1WN8.

	Energy Deviations / $kJmol^{-1}$								Rel. Energy / $kJmol^{-1}$
Conf.	MFHC-[$Nei2_{4.0}$]			MIM-[$\eta = 9$]		$PAIR^{HB}_{2.2}$-[$Nei2_{4.0}$]			Super
	[Hier2]	[Super]	[ee]	[Super]	[ee]	[Hier2]	[Super]	[ee]	PBEh-3c
1	10.81	16.93	4.56	9.07	14.66	103.70	49.55	1.13	-367.76
2	20.86	4.50	5.85	0.02	7.71	67.43	31.62	6.27	-208.27
3	0.44	10.49	13.28	4.22	10.39	101.85	50.40	15.32	-362.13
4	77.89	37.96	12.87	22.10	5.54	90.01	40.80	7.83	-265.87
5	38.22	15.17	6.71	50.85	0.82	34.18	13.34	2.19	-295.74
6	68.11	20.07	0.93	3.40	15.12	68.65	31.08	0.53	-350.19
7	10.89	1.79	9.13	9.22	10.66	42.92	20.38	3.01	-447.22
8	96.04	41.99	12.09	22.72	5.61	60.23	21.65	11.25	-135.51
9	66.07	27.78	13.96	11.44	16.07	34.62	12.78	15.59	-122.57
10	61.65	35.41	3.75	5.67	2.48	75.76	37.85	8.76	-221.10
11	13.73	3.21	8.13	18.66	2.46	58.62	32.90	16.18	-272.33
12	78.36	36.07	2.05	26.11	34.65	80.81	36.73	13.91	-293.51
13	22.14	13.74	11.10	11.04	12.66	65.31	28.62	8.06	-112.45
14	0.13	3.13	1.23	4.30	4.97	68.34	31.06	12.14	-122.30
15	4.79	1.51	0.29	4.12	4.05	69.88	30.51	3.20	-206.26
16	14.22	11.50	9.89	9.37	7.95	30.73	18.02	14.86	-149.29
17	51.97	24.54	7.20	2.30	19.26	54.53	23.70	1.45	-181.26
18	0.00	0.00	0.00	0.00	0.00	0.00	0.00	0.00	0.00
19	88.95	35.72	9.00	12.27	7.91	49.02	24.91	4.42	-101.01
20	2.63	0.42	3.19	4.60	15.46	5.31	4.30	9.45	-28.33
MAD	36.39	17.10	6.76	11.57	9.92	58.09	27.01	7.78	

Table S-XVII: Deviations of the relative energies of 2RT4.

Conf.	Energy Deviations / kJmol^{-1}								Rel. Energy / kJmol^{-1}
	MFHC-[Nei2$_{4.0}$]			MIM-[$\eta = 9$]		PAIR$^{\text{HB}}_{2.2}$-[Nei2$_{4.0}$]			Super
	[Hier2]	[Super]	[ee]	[Super]	[ee]	[Hier2]	[Super]	[ee]	PBEh-3c
1	39.14	21.00	13.23	10.29	13.95	5.16	3.24	1.10	-353.13
2	33.20	11.43	11.68	12.28	34.08	17.89	11.10	5.98	-142.86
3	6.85	1.67	0.27	52.47	54.01	2.86	2.32	5.36	-84.57
4	21.69	4.20	9.88	23.80	16.44	20.20	5.49	11.57	-279.84
5	118.11	53.68	24.73	51.64	23.97	17.61	3.25	0.42	-451.58
6	13.97	0.64	19.25	2.25	13.56	10.88	0.46	1.83	-200.71
7	21.51	10.02	15.51	34.01	17.08	0.23	1.36	7.45	-192.57
8	119.53	45.38	14.79	105.53	74.64	50.52	19.60	6.04	-530.41
9	8.64	0.71	15.79	23.62	17.15	18.62	9.16	8.56	-235.90
10	68.63	17.79	19.18	38.17	18.23	0.65	0.51	2.07	-234.61
11	51.81	28.52	12.72	70.06	43.22	12.82	6.12	3.03	-383.60
12	52.08	20.22	24.56	33.62	20.76	7.03	1.48	6.40	-335.04
13	11.53	4.35	12.90	2.82	21.01	7.20	4.63	9.48	-215.78
14	32.02	10.68	25.67	44.89	46.03	11.48	4.66	8.81	-140.05
15	112.26	40.68	24.16	113.26	61.99	32.36	12.06	5.96	-448.58
16	57.02	19.36	10.66	45.57	36.12	8.79	0.20	2.95	-346.03
17	119.34	40.90	24.50	73.34	27.63	26.74	10.20	0.91	-520.80
18	31.55	10.49	23.59	23.35	27.86	14.52	3.84	2.70	-303.10
19	57.71	25.55	16.59	22.98	1.04	9.76	8.56	3.24	-323.24
20	0.00	0.00	0.00	0.00	0.00	0.00	0.00	0.00	0.00
MAD	48.83	18.36	15.98	39.20	28.44	13.77	5.41	4.69	

Table S-XVIII: Deviations of the relative energies of 1EWS.

Conf.	Energy Deviations / kJmol^{-1}								Rel. Energy / kJmol^{-1}
	MFHC-[Nei2$_{4.0}$]			MIM-[$\eta = 9$]		PAIR$^{\text{HB}}_{2.2}$-[Nei2$_{4.0}$]			Super
	[Hier2]	[Super]	[ee]	[Super]	[ee]	[Hier2]	[Super]	[ee]	PBEh-3c
1	23.70	13.50	13.69	15.43	4.99	13.20	6.47	4.24	-29.32
2	130.17	67.79	24.78	84.21	159.10	58.81	35.54	12.24	-896.48
3	0.00	0.00	0.00	0.00	0.00	0.00	0.00	0.00	0.00
4	75.12	36.87	7.28	99.85	3.34	22.67	8.29	6.13	-152.39
5	4.71	5.21	12.31	35.51	7.10	0.47	10.58	5.68	-258.96
6	7.82	7.49	23.10	28.68	39.10	64.39	33.64	19.20	-416.68
7	16.78	9.80	13.23	58.67	71.90	45.56	20.63	9.91	-153.82
8	64.52	14.09	8.81	50.40	7.64	7.27	4.24	0.68	-693.40
9	44.17	16.39	8.19	31.81	21.76	21.02	3.78	17.27	-445.50
10	59.90	26.77	16.58	9.09	31.88	14.58	7.18	3.81	-426.84
11	54.91	32.31	34.51	21.99	42.46	21.47	7.71	16.90	-519.09
12	85.08	8.03	0.17	46.39	34.75	121.87	51.34	9.35	-154.99
13	34.91	10.24	11.43	60.93	25.71	141.83	70.44	12.91	-504.54
14	25.98	12.89	5.81	46.51	16.78	6.77	10.95	4.96	-403.28
15	95.90	42.06	34.74	41.42	13.47	147.72	57.74	6.42	-270.55
16	38.18	9.84	5.95	55.36	24.04	31.93	11.77	6.41	-290.19
17	76.05	33.33	4.34	26.53	23.61	69.13	35.31	3.58	-209.13
18	81.80	44.10	4.26	68.00	41.02	21.71	22.47	31.68	-818.58
19	47.21	20.32	26.29	18.37	28.12	8.75	9.49	8.42	-665.84
20	49.23	19.59	7.24	48.66	48.79	35.64	19.61	1.08	-3238.38
MAD	50.81	21.53	13.14	42.39	32.28	42.74	21.36	9.04	

Table S-XIX: Deviations of the relative energies of 2LEW.

Conf.	Energy Deviations / $kJmol^{-1}$ MFHC-[$Nei2_{4.0}$] [Hier2]	MFHC-[$Nei2_{4.0}$] [Super]	MFHC-[$Nei2_{4.0}$] [ee]	MIM-[$\eta = 9$] [Super]	MIM-[$\eta = 9$] [ee]	PAIR$^{HB}_{2.2}$-[$Nei2_{4.0}$] [Hier2]	PAIR$^{HB}_{2.2}$-[$Nei2_{4.0}$] [Super]	PAIR$^{HB}_{2.2}$-[$Nei2_{4.0}$] [ee]	Rel. Energy / $kJmol^{-1}$ Super PBEh-3c
1	90.29	38.36	29.68	19.54	27.70	17.84	6.17	11.61	-224.11
2	85.66	37.44	4.28	2.18	61.15	43.35	30.46	0.81	-263.21
3	112.07	49.42	2.92	59.06	23.28	37.41	26.95	2.44	-284.86
4	81.80	32.46	4.33	19.32	19.63	17.44	14.03	1.07	-360.89
5	58.17	23.86	14.71	2.37	42.78	54.74	19.41	8.66	-240.15
6	74.50	36.58	7.89	15.23	49.27	29.37	19.23	4.97	-272.52
7	40.97	14.64	16.85	0.99	61.87	27.26	16.52	0.53	-146.06
8	0.00	0.00	0.00	0.00	0.00	0.00	0.00	0.00	0.00
9	59.20	33.30	6.87	1.14	34.64	80.30	47.78	0.55	-75.56
10	88.99	54.48	6.71	22.28	34.60	68.97	41.72	11.68	-76.16
11	63.99	36.57	0.67	18.29	52.51	16.34	21.00	14.05	-192.65
12	91.05	40.87	12.92	32.42	15.67	66.38	20.59	8.53	-69.13
13	71.54	30.48	10.25	22.70	12.09	1.85	7.95	11.10	-147.48
14	35.21	10.65	6.06	2.88	33.81	9.98	11.26	5.81	-230.76
15	84.43	39.29	3.90	44.02	68.21	85.47	32.14	17.46	-135.20
16	4.11	5.09	13.24	6.02	32.74	21.01	12.52	6.83	-237.71
17	55.15	24.52	19.58	9.88	35.56	62.73	35.06	13.49	-357.74
18	83.61	24.84	4.95	5.75	65.64	14.74	16.41	12.26	-177.06
19	81.80	33.78	11.58	74.15	23.89	9.56	1.94	4.87	-149.83
20	60.31	31.91	10.70	17.43	44.62	8.15	1.42	9.98	-65.84
MAD	66.14	29.93	9.40	18.78	36.98	33.64	19.13	7.34	

Table S-XX: Deviations of the relative energies of 2GW9.

Conf.	Energy Deviations / $kJmol^{-1}$ MFHC-[$Nei2_{4.0}$] [Hier2]	MFHC-[$Nei2_{4.0}$] [Super]	MFHC-[$Nei2_{4.0}$] [ee]	MIM-[$\eta = 9$] [Super]	MIM-[$\eta = 9$] [ee]	PAIR$^{HB}_{2.2}$-[$Nei2_{4.0}$] [Hier2]	PAIR$^{HB}_{2.2}$-[$Nei2_{4.0}$] [Super]	PAIR$^{HB}_{2.2}$-[$Nei2_{4.0}$] [ee]	Rel. Energy / $kJmol^{-1}$ Super PBEh-3c
1	0.99	3.26	12.00	8.35	60.94	59.93	27.33	9.09	-205.40
2	28.08	1.25	5.81	13.40	3.69	15.57	3.49	4.04	-134.16
3	25.74	13.65	10.83	9.27	16.02	172.52	84.81	20.62	-176.60
4	23.85	7.06	5.18	15.96	12.41	11.60	1.35	0.97	-366.85
5	37.32	5.63	13.62	6.12	44.65	38.15	12.00	1.34	-92.08
6	10.60	8.54	7.58	16.71	25.66	45.72	17.86	6.14	-10.91
7	20.32	2.53	13.81	2.03	34.61	3.00	8.63	7.70	-252.41
8	0.00	0.00	0.00	0.00	0.00	0.00	0.00	0.00	0.00
9	43.64	2.85	8.42	2.94	5.66	61.86	23.47	22.98	-52.35
10	14.10	26.57	9.78	29.62	37.80	31.26	7.86	1.65	-37.20
11	7.07	1.64	15.58	5.62	4.23	62.65	31.96	5.17	-220.25
12	15.11	6.16	9.64	29.91	14.68	6.81	4.25	2.60	-73.36
13	26.70	4.74	15.94	24.82	58.12	11.44	10.12	2.11	-243.64
14	8.85	4.10	19.80	35.01	43.56	12.19	15.42	2.37	-216.83
15	63.65	36.84	7.79	16.46	66.91	34.37	15.94	10.10	-206.32
16	30.52	13.45	6.05	49.92	19.54	70.91	35.09	24.11	-130.91
17	20.81	12.86	2.62	26.20	10.66	81.06	41.39	34.35	-158.17
18	11.43	0.83	11.92	42.54	59.11	84.93	41.70	11.94	-20.79
19	9.50	3.40	22.14	30.61	20.38	65.57	29.54	0.84	-151.18
20	51.65	29.63	0.32	42.53	33.72	90.58	48.07	22.20	-314.45
MAD	22.50	9.25	9.94	20.40	28.62	48.01	23.01	9.52	

Table S-XXI: Deviations of the relative energies of 2LG5.

Conf.	Energy Deviations / $kJmol^{-1}$								Rel. Energy / $kJmol^{-1}$
	MFHC-[Nei2$_{4.0}$]			MIM-[$\eta = 9$]		PAIR$^{HB}_{2.2}$-[Nei2$_{4.0}$]			Super
	[Hier2]	[Super]	[ee]	[Super]	[ee]	[Hier2]	[Super]	[ee]	PBEh-3c
1	35.74	27.82	19.64	47.69	40.61	29.77	7.34	3.25	-539.62
2	0.00	0.00	0.00	0.00	0.00	0.00	0.00	0.00	0.00
3	77.42	34.65	2.27	39.76	38.22	11.03	3.70	16.05	-496.42
4	28.38	0.67	6.68	21.90	3.77	1.20	0.42	9.73	-418.44
5	39.04	13.98	0.40	41.76	65.47	32.68	3.25	3.09	-142.41
6	15.52	15.82	7.01	66.36	26.26	18.38	3.62	2.75	-425.84
7	15.21	6.21	10.76	14.07	40.13	25.50	1.19	2.66	-157.27
8	34.16	19.20	35.61	6.26	54.34	57.32	18.24	11.11	-494.78
9	50.90	17.37	14.18	38.04	66.69	22.93	12.42	0.64	-543.92
10	14.86	12.86	4.16	42.77	25.28	21.18	16.15	11.58	-383.68
MAD	31.12	14.86	10.07	31.86	36.08	22.00	6.63	6.09	

Table S-XXII: Deviations of the relative energies of 2M9E.

Conf.	Energy Deviations / $kJmol^{-1}$								Rel. Energy / $kJmol^{-1}$
	MFHC-[Nei2$_{4.0}$]			MIM-[$\eta = 9$]		PAIR$^{HB}_{2.2}$-[Nei2$_{4.0}$]			Super
	[Hier2]	[Super]	[ee]	[Super]	[ee]	[Hier2]	[Super]	[ee]	PBEh-3c
1	249.35	74.97	7.77	67.21	57.99	19.35	6.13	14.70	-60.95
2	60.69	1.91	2.84	39.81	2.12	66.11	35.88	26.64	-211.40
3	154.41	46.77	11.71	68.67	70.80	27.29	7.77	11.27	-358.30
4	119.90	8.49	11.48	30.67	24.04	19.55	1.33	11.23	-196.90
5	191.25	34.21	11.42	8.58	34.97	80.78	33.83	15.08	-199.31
6	0.00	0.00	0.00	0.00	0.00	0.00	0.00	0.00	0.00
7	141.77	58.38	3.80	76.12	32.85	52.91	1.46	1.03	-427.30
8	217.31	60.71	2.90	47.44	39.51	1.88	14.57	1.15	-175.11
9	37.76	3.18	7.97	102.43	68.03	43.54	6.28	4.62	-350.85
10	136.24	25.18	5.45	53.59	51.95	60.96	34.06	5.20	-181.05
11	191.37	48.87	6.20	84.88	58.30	37.53	26.35	42.03	-249.02
12	206.69	61.90	15.92	11.06	45.73	23.81	19.95	19.25	-156.09
13	290.81	80.73	4.13	11.35	31.78	21.79	18.05	26.86	-231.28
14	132.46	23.51	16.06	36.47	17.97	26.54	27.01	4.17	-123.79
15	216.43	63.09	8.86	20.82	19.79	20.39	12.97	8.08	-279.11
16	150.97	54.01	11.97	10.20	38.02	8.57	13.63	7.93	-259.44
17	124.35	30.97	21.15	26.99	35.28	32.37	24.85	0.32	-286.37
18	191.33	68.61	17.83	24.85	28.24	28.87	6.66	4.90	-107.74
19	135.50	27.80	17.25	15.07	80.59	3.96	9.88	7.05	-298.83
20	229.96	73.02	10.72	25.16	8.00	60.88	32.47	46.56	-207.11
MAD	158.93	42.32	9.77	38.07	37.30	31.86	16.66	12.90	

Table S-XXIII: Deviations of the relative energies of 2NC3.

Conf.	Energy Deviations / kJmol^{-1}								Rel. Energy / kJmol^{-1}
	MFHC-[Nei2$_{4.0}$]			MIM-[$\eta = 9$]		PAIR$^{HB}_{2.2}$-[Nei2$_{4.0}$]			Super
	[Hier2]	[Super]	[ee]	[Super]	[ee]	[Hier2]	[Super]	[ee]	PBEh-3c
1	140.58	36.30	26.79	19.42	6.00	8.87	1.92	1.54	-141.29
2	174.43	51.21	24.86	15.82	12.99	26.83	20.64	5.44	-205.71
3	4.36	13.18	0.36	16.97	1.73	13.49	11.43	4.41	-231.67
4	29.98	23.31	12.15	47.65	17.20	17.21	3.34	5.03	-309.20
5	128.52	32.81	33.92	43.54	45.61	0.39	4.61	1.89	-203.16
6	22.87	4.69	4.80	41.85	96.13	34.91	23.33	13.12	-397.63
7	131.38	38.80	20.42	26.44	20.77	20.53	15.82	8.52	-111.39
8	146.93	42.61	33.94	0.33	32.75	24.64	23.87	15.14	-39.29
9	27.86	25.71	8.73	39.24	0.83	56.76	17.72	7.62	-193.13
10	155.27	48.01	16.34	51.45	34.67	26.32	25.44	11.34	-176.58
11	73.70	19.47	3.40	33.22	40.78	14.75	16.82	21.22	-324.97
12	35.17	25.80	6.16	36.34	23.75	33.94	19.08	3.01	-253.55
13	98.37	24.96	10.01	13.66	30.47	4.11	8.81	9.13	-204.23
14	162.50	48.88	34.04	6.33	31.87	19.78	13.52	6.16	-125.46
15	54.25	32.56	6.34	97.35	46.76	39.04	19.37	19.34	-353.44
16	0.00	0.00	0.00	0.00	0.00	0.00	0.00	0.00	0.00
17	122.47	31.76	20.77	38.76	25.10	3.54	9.33	13.79	-158.54
18	50.25	24.51	1.29	30.20	41.95	12.68	4.39	5.70	-324.67
19	155.95	46.01	34.22	8.19	0.51	3.91	8.02	9.17	-130.56
20	164.13	53.64	17.62	13.37	8.14	12.31	17.43	1.76	-279.23
MAD	93.95	31.21	15.81	29.01	25.90	18.70	13.25	8.17	

Table S-XXIV: Deviations of the relative energies of 2KCF.

Conf.	Energy Deviations / kJmol^{-1}								Rel. Energy / kJmol^{-1}
	MFHC-[Nei2$_{4.0}$]			MIM-[$\eta = 9$]		PAIR$^{HB}_{2.2}$-[Nei2$_{4.0}$]			Super
	[Hier2]	[Super]	[ee]	[Super]	[ee]	[Hier2]	[Super]	[ee]	PBEh-3c
1	109.14	29.30	26.69	142.35	48.74	70.79	40.92	14.98	-550.78
2	63.78	22.27	13.06	70.12	66.79	40.79	29.69	8.66	-297.42
3	5.55	12.70	6.24	57.32	15.71	74.51	30.71	8.76	-347.89
4	85.74	45.91	23.70	1.40	42.56	106.72	48.99	2.64	-197.96
5	78.50	28.90	16.28	42.00	0.05	95.30	40.33	4.10	-499.92
6	65.32	20.10	21.55	13.07	10.31	116.33	59.37	11.18	-487.50
7	51.23	14.41	38.50	120.48	85.10	63.12	24.88	1.61	-578.84
8	45.72	32.59	8.88	39.44	6.32	158.04	83.81	43.91	-457.71
9	32.95	10.38	11.39	87.28	16.08	82.75	38.07	1.49	-329.78
10	9.57	17.71	12.46	3.87	13.09	50.11	29.01	3.64	-279.75
11	59.43	16.96	24.24	61.73	14.80	116.42	61.73	18.46	-313.56
12	39.18	14.43	31.24	63.33	62.29	68.03	39.88	22.66	-193.39
13	32.14	0.78	0.14	28.29	35.89	68.00	37.75	15.03	-319.87
14	176.29	56.91	17.00	120.13	44.99	59.19	30.93	19.16	-590.13
15	0.00	0.00	0.00	0.00	0.00	0.00	0.00	0.00	0.00
16	27.43	2.87	2.17	94.75	13.27	107.82	63.08	17.51	-360.05
17	99.97	24.01	23.36	20.16	4.07	71.70	48.92	7.47	-476.99
18	11.57	8.60	14.29	23.13	20.24	64.14	37.13	23.27	-409.28
19	56.96	36.98	10.15	3.16	26.23	30.24	25.96	9.75	-240.57
20	102.19	25.20	35.64	102.67	34.13	116.76	54.22	3.48	-697.30
MAD	57.63	21.05	16.85	54.73	28.03	78.04	41.27	11.89	

Table S-XXV: Deviations of the relative energies of 2KYJ.

	Energy Deviations / $kJmol^{-1}$								Rel. Energy / $kJmol^{-1}$
Conf.	MFHC-[$Nei2_{4.0}$]			MIM-[$\eta = 9$]		$PAIR^{HB}_{2.2}$-[$Nei2_{4.0}$]			Super
	[Hier2]	[Super]	[ee]	[Super]	[ee]	[Hier2]	[Super]	[ee]	PBEh-3c
1	14.33	3.84	2.62	47.33	80.82	4.86	1.60	0.99	-227.05
2	224.58	70.17	16.82	196.69	91.02	8.16	2.15	9.00	-659.87
3	83.51	21.88	3.24	30.56	58.69	0.20	0.17	16.09	-227.36
4	46.68	19.84	4.33	101.72	74.13	4.30	10.25	12.62	-376.85
5	9.20	3.13	6.70	45.18	94.93	60.86	27.87	11.53	-91.00
6	90.73	25.64	7.72	9.09	40.94	7.70	5.00	4.47	-85.82
7	0.00	0.00	0.00	0.00	0.00	0.00	0.00	0.00	0.00
8	65.66	17.81	10.39	77.36	69.67	40.65	14.32	11.54	-431.89
9	50.79	23.96	1.41	133.81	74.41	21.13	18.61	16.83	-554.56
10	68.74	23.73	7.43	120.70	68.82	24.89	23.49	12.69	-352.78
11	47.04	19.62	10.43	138.63	72.84	53.20	21.78	2.52	-360.39
12	115.71	35.78	11.78	72.54	40.00	21.32	8.48	16.92	-383.20
13	130.66	33.68	4.62	60.67	59.42	90.17	31.38	9.71	-384.50
14	3.36	8.43	15.89	1.94	0.86	6.63	1.09	12.64	-76.92
15	45.99	15.30	0.56	99.36	63.66	2.06	3.90	11.60	-390.93
16	45.82	14.09	3.10	73.61	46.83	13.42	3.15	2.63	-193.45
17	94.92	29.08	6.65	25.54	29.28	34.77	9.60	5.22	-274.08
18	82.13	25.29	0.21	24.86	125.70	27.29	2.25	8.34	-338.04
19	32.87	5.16	9.87	48.77	70.43	44.05	17.92	3.98	-246.29
20	41.97	11.04	8.84	68.46	15.96	7.68	3.85	12.01	-387.56
MAD	64.73	20.37	6.63	68.84	58.92	23.67	10.34	9.07	

Table S-XXVI: Deviations of the relative energies of 1AML.

	Energy Deviations / $kJmol^{-1}$								Rel. Energy / $kJmol^{-1}$
Conf.	MFHC-[$Nei2_{4.0}$]			MIM-[$\eta = 9$]		$PAIR^{HB}_{2.2}$-[$Nei2_{4.0}$]			Super
	[Hier2]	[Super]	[ee]	[Super]	[ee]	[Hier2]	[Super]	[ee]	PBEh-3c
1	110.01	51.75	36.25	85.20	8.93	33.39	14.67	13.17	-910.89
2	0.67	1.40	18.61	1.15	13.62	21.65	9.04	3.04	-424.53
3	28.10	7.69	18.02	10.14	7.39	6.18	5.47	7.25	-395.80
4	33.78	9.38	6.45	21.73	20.33	16.56	5.71	1.60	-417.05
5	0.00	0.00	0.00	0.00	0.00	0.00	0.00	0.00	0.00
6	64.72	23.23	8.96	10.05	2.46	25.68	3.90	0.59	-351.12
7	122.84	46.71	22.34	47.02	14.13	43.32	13.02	8.75	-459.91
8	16.95	6.38	19.80	6.67	38.49	26.01	14.76	3.24	-124.58
9	82.56	14.01	38.52	44.55	1.28	36.49	9.23	14.14	-369.45
10	60.86	19.35	9.94	14.88	12.23	9.08	2.91	7.34	-257.99
11	106.68	38.36	11.52	23.16	3.12	50.33	21.96	1.73	-138.04
12	100.73	40.23	25.34	34.50	64.59	34.21	21.26	3.26	-349.32
13	24.49	2.03	30.11	27.35	46.18	10.66	14.64	6.10	-109.82
14	105.20	19.09	19.64	1.48	16.42	4.68	3.07	5.20	-509.48
15	70.94	25.91	9.00	37.66	2.27	17.06	5.53	9.80	-373.47
16	72.65	21.77	14.28	10.88	42.56	2.04	0.72	2.50	-165.08
17	115.49	50.61	6.72	98.56	42.92	1.06	3.82	18.63	-681.44
18	113.36	46.50	24.00	30.60	9.15	41.25	16.38	9.56	-471.76
19	26.66	2.70	16.25	6.25	11.03	5.15	0.47	11.38	-519.37
20	60.46	18.87	23.46	3.20	26.71	30.28	6.61	8.25	-381.39
MAD	65.86	22.30	17.96	25.75	19.19	20.75	8.66	6.78	

Table S-XXVII: Deviations of the relative energies of 5KPH.

Conf.	Energy Deviations / kJmol^{-1}								Rel. Energy / kJmol^{-1}
	MFHC-[Nei2$_{4.0}$]			MIM-[$\eta = 9$]		PAIR$^{HB}_{2.2}$-[Nei2$_{4.0}$]			Super
	[Hier2]	[Super]	[ee]	[Super]	[ee]	[Hier2]	[Super]	[ee]	PBEh-3c
2	368.61	132.00	10.54	140.13	68.93	139.30	76.20	8.01	-896.34
4	330.90	77.45	35.79	47.25	40.68	259.01	122.03	5.09	-104.22
5	330.22	128.04	1.80	6.88	0.55	84.91	37.06	1.42	-616.98
6	339.60	128.48	6.05	196.67	72.38	196.84	82.01	14.20	-840.19
8	231.21	80.19	2.29	80.53	71.21	121.78	63.36	16.10	-269.51
9	392.89	150.90	12.17	24.95	77.54	126.87	70.17	22.13	-620.51
10	230.30	72.48	12.76	6.67	24.73	47.49	2.74	30.12	-225.24
12	190.72	67.30	24.46	71.01	27.27	63.99	22.97	30.97	-274.06
13	248.66	135.35	14.04	23.31	48.52	50.08	34.96	7.89	-182.94
14	239.37	87.35	17.08	39.28	12.00	132.28	46.07	3.05	-693.91
17	55.84	13.06	17.16	3.05	22.34	162.43	67.42	37.26	-494.98
20	0.00	0.00	0.00	0.00	0.00	0.00	0.00	0.00	0.00
MAD	246.53	89.38	12.85	53.31	38.85	115.42	52.08	14.69	

Run Times

Table S-XXVIII: Run Times of the fragmented calculations of 1WN8.

Conf.	Run Time / h							
	MFHC-[Nei2$_{4.0}$]			MIM-[$\eta = 9$]		PAIR$^{HB}_{2.2}$-[Nei2$_{4.0}$]		
	[Hier2]	[Super]	[ee]	[Super]	[ee]	[Hier2]	[Super]	[ee]
1	15.64	16.68	15.73	28.50	27.07	57.57	57.43	54.88
2	12.67	13.73	12.59	32.90	31.67	50.10	49.72	47.67
3	15.18	15.54	14.62	32.88	31.41	58.38	57.58	56.22
4	15.41	16.05	15.28	31.45	30.37	55.76	55.86	53.28
5	12.40	13.36	12.29	30.67	29.71	59.86	59.45	58.54
6	12.52	13.19	12.43	33.53	32.12	54.23	53.33	52.23
7	14.15	14.94	13.94	31.61	27.49	64.09	63.35	62.23
8	15.15	16.25	15.17	30.19	26.63	56.69	57.25	55.07
9	15.61	16.37	15.40	33.85	32.99	55.99	55.57	54.21
10	15.25	15.82	14.90	32.14	30.94	47.80	47.26	46.77
11	11.72	12.51	11.55	30.14	28.60	58.22	57.66	56.03
12	16.30	17.14	16.31	25.97	25.07	60.10	60.06	57.34
13	12.05	13.04	12.18	30.99	29.80	46.20	45.53	44.74
14	12.44	13.23	12.25	34.15	32.62	51.38	50.97	49.31
15	12.04	12.78	11.93	30.47	26.98	47.57	47.15	45.17
16	14.36	15.24	14.16	33.76	29.65	57.52	57.40	56.19
17	16.91	18.03	16.85	30.47	29.59	50.71	49.73	47.69
18	14.47	15.24	14.09	30.01	28.67	56.39	56.02	54.54
19	17.11	17.79	17.08	28.96	28.26	57.21	56.88	54.28
20	12.74	14.86	12.74	30.36	29.29	58.31	58.68	56.82
Avrg.	14.21	15.09	14.08	31.15	29.45	55.13	54.84	53.16

Table S-XXIX: Run Times of the fragmented calculations of 2RT4.

Conf.	Run Time / h							
	MFHC-[Nei2$_{4.0}$]			MIM-[$\eta = 9$]		PAIR$^{\mathrm{HB}}_{2.2}$-[Nei2$_{4.0}$]		
	[Hier2]	[Super]	[ee]	[Super]	[ee]	[Hier2]	[Super]	[ee]
1	15.12	15.76	14.28	20.02	19.45	45.56	47.34	44.99
2	14.95	15.47	14.32	18.43	18.50	47.54	49.65	47.39
3	14.52	15.18	13.79	20.72	19.97	46.65	48.13	45.58
4	14.81	16.01	14.37	23.11	22.44	42.34	43.04	42.14
5	14.70	15.82	14.22	17.73	17.29	50.85	52.41	50.48
6	14.24	15.17	13.77	21.44	20.97	41.68	43.53	41.80
7	14.68	15.56	14.27	23.78	22.91	44.15	44.73	43.70
8	15.88	17.00	15.37	21.55	20.53	50.48	51.46	50.20
9	15.04	16.29	14.46	20.94	20.50	43.39	45.01	43.57
10	14.26	14.83	13.79	18.07	18.32	44.65	45.45	44.15
11	14.77	15.10	14.30	22.26	22.69	42.68	42.84	41.47
12	15.02	15.40	14.59	23.10	23.06	45.58	46.09	44.21
13	13.98	14.49	13.84	16.88	17.13	38.67	39.32	38.18
14	15.80	16.36	15.05	20.73	20.29	43.09	43.93	42.67
15	14.91	15.14	14.45	19.37	19.18	50.26	50.94	49.67
16	15.07	15.86	14.72	21.18	20.59	52.67	53.56	52.43
17	14.99	15.60	14.58	21.33	21.00	54.36	55.22	53.70
18	13.99	14.50	13.91	17.63	17.72	48.37	48.50	47.16
19	14.57	15.13	14.18	19.38	19.94	44.55	45.90	45.01
20	13.20	13.78	12.79	19.61	20.05	38.77	39.53	37.93
Avrg.	14.72	15.42	14.25	20.36	20.13	45.81	46.83	45.32

Table S-XXX: Run Times of the fragmented calculations of 1EWS.

Conf.	Run Time / h							
	MFHC-[Nei2$_{4.0}$]			MIM-[$\eta = 9$]		PAIR$^{\mathrm{HB}}_{2.2}$-[Nei2$_{4.0}$]		
	[Hier2]	[Super]	[ee]	[Super]	[ee]	[Hier2]	[Super]	[ee]
1	24.08	28.72	23.55	35.66	31.27	163.90	167.05	157.48
2	28.40	33.23	27.37	33.63	29.71	160.39	166.35	155.32
3	23.99	28.59	23.67	35.96	32.16	140.22	147.12	138.38
4	31.19	35.83	30.18	35.84	31.60	135.26	143.41	136.14
5	25.64	30.05	25.48	33.87	30.60	147.46	152.33	145.15
6	33.27	37.70	32.11	31.40	27.63	160.15	164.88	157.66
7	25.02	29.05	24.51	32.83	30.37	147.12	151.69	147.28
8	26.07	30.29	25.65	34.32	31.29	164.49	169.80	157.92
9	31.96	35.20	31.18	33.88	31.34	158.52	162.19	154.85
10	32.83	37.18	32.11	36.14	31.80	139.43	145.40	137.14
11	26.00	30.04	25.87	32.81	29.14	156.63	163.41	153.52
12	26.73	30.46	26.46	35.09	31.60	139.97	145.30	139.69
13	32.47	36.50	32.15	32.63	29.43	152.68	158.72	148.84
14	29.40	32.94	29.25	34.88	32.13	131.52	136.30	131.32
15	33.42	38.43	33.74	35.39	31.34	146.84	152.11	146.46
16	28.00	31.57	26.45	36.53	31.73	148.04	153.36	144.53
17	32.28	35.21	29.76	33.82	30.90	152.49	157.64	146.07
18	29.08	33.49	29.16	35.69	31.70	156.47	160.70	150.78
19	28.10	31.84	26.99	33.64	30.26	162.69	168.86	162.91
20	29.10	32.05	28.22	31.31	28.24	137.96	144.31	137.79
Avrg.	28.85	32.92	28.19	34.27	30.71	150.11	155.55	147.46

Table S-XXXI: Run Times of the fragmented calculations of 2LEW.

Conf.	Run Time / h							
	MFHC-[Nei$2_{4.0}$]			MIM-[$\eta = 9$]		PAIR$^{HB}_{2.2}$-[Nei$2_{4.0}$]		
	[Hier2]	[Super]	[ee]	[Super]	[ee]	[Hier2]	[Super]	[ee]
1	27.69	28.55	27.24	31.66	31.24	131.88	132.96	131.54
2	26.03	29.44	25.78	31.14	28.76	134.96	140.06	134.05
3	26.54	29.46	26.22	27.72	25.83	139.32	143.86	139.52
4	25.69	28.85	24.99	29.62	27.26	133.63	138.71	130.42
5	26.07	29.61	25.30	30.38	27.47	131.96	139.52	132.38
6	27.46	30.09	27.11	31.13	28.71	139.26	142.74	136.96
7	25.78	29.07	25.57	31.29	29.35	127.52	132.36	126.91
8	26.43	29.72	26.23	32.34	29.43	127.54	129.70	123.81
9	27.69	32.09	27.53	34.89	31.62	134.03	139.49	131.10
10	25.54	27.88	25.37	27.56	26.32	117.58	121.86	118.86
11	26.27	28.56	25.95	29.55	28.64	133.82	138.00	132.71
12	27.05	28.91	26.49	31.00	30.01	130.63	134.69	129.78
13	27.27	29.76	26.97	32.46	30.34	134.78	141.35	135.58
14	26.25	29.90	25.92	28.01	26.13	130.91	135.87	130.36
15	26.28	29.23	26.07	28.32	26.39	130.22	133.72	128.46
16	27.10	27.88	27.23	28.49	28.34	138.09	137.33	133.87
17	26.96	27.90	26.58	26.84	26.93	125.56	126.09	124.91
18	25.71	26.30	25.51	29.05	29.44	138.62	143.17	137.30
19	27.44	27.63	26.42	28.03	28.68	120.15	121.82	119.14
20	27.56	27.77	27.15	29.57	29.35	136.64	139.23	136.38
Avrg.	26.64	28.93	26.28	29.95	28.51	131.85	135.63	130.70

Table S-XXXII: Run Times of the fragmented calculations of 2GW9.

Conf.	Run Time / h							
	MFHC-[Nei$2_{4.0}$]			MIM-[$\eta = 9$]		PAIR$^{HB}_{2.2}$-[Nei$2_{4.0}$]		
	[Hier2]	[Super]	[ee]	[Super]	[ee]	[Hier2]	[Super]	[ee]
1	27.42	30.38	26.03	31.04	28.64	144.74	148.85	141.21
2	27.89	30.39	26.63	31.72	29.62	149.45	155.93	148.66
3	27.78	30.04	26.54	33.16	30.40	154.71	158.77	152.32
4	26.79	27.82	25.59	29.26	28.11	153.35	155.20	151.38
5	27.84	30.24	27.21	34.05	31.93	157.58	163.59	157.58
6	26.67	29.22	26.11	29.12	27.22	150.26	155.60	148.90
7	28.60	29.88	27.56	30.82	29.38	149.37	149.81	145.05
8	29.38	31.55	28.00	30.99	28.52	155.77	159.83	153.14
9	27.13	29.01	25.58	30.85	28.97	170.11	174.17	169.11
10	27.71	28.90	26.30	31.34	30.27	148.41	154.16	148.06
11	28.84	31.17	27.73	32.73	29.95	154.30	160.38	151.93
12	28.18	30.24	27.10	30.14	28.49	156.02	158.26	152.79
13	27.17	31.42	25.91	33.29	28.96	145.41	149.90	143.23
14	26.61	29.24	25.17	34.43	31.47	151.64	158.33	151.77
15	26.96	31.84	26.33	35.73	31.90	152.19	158.00	150.51
16	27.41	30.85	26.30	33.52	30.36	152.14	156.89	149.32
17	27.85	30.86	27.23	33.49	30.50	151.51	157.93	152.74
18	26.90	29.75	25.84	33.44	30.86	142.81	146.63	139.93
19	28.02	32.29	27.06	33.95	30.02	147.81	152.28	144.13
20	26.72	28.76	25.75	31.30	29.68	145.23	150.41	143.75
Avrg.	27.59	30.19	26.50	32.22	29.76	151.64	156.24	149.78

Table S-XXXIII: Run Times of the fragmented calculations of 2LG5.

Conf.	Run Time / h							
	MFHC-[Nei2$_{4.0}$]			MIM-[$\eta = 9$]		PAIR$^{\mathrm{HB}}_{2.2}$-[Nei2$_{4.0}$]		
	[Hier2]	[Super]	[ee]	[Super]	[ee]	[Hier2]	[Super]	[ee]
1	28.02	30.02	27.25	36.26	34.69	158.76	162.57	159.17
2	27.98	29.85	27.46	39.47	38.04	157.42	160.59	158.42
3	25.46	27.47	25.51	34.91	33.92	144.54	148.80	142.59
4	27.61	30.48	27.32	39.98	38.20	147.66	151.46	146.32
5	26.74	29.05	26.56	35.17	34.22	151.79	158.04	150.72
6	26.61	29.17	26.28	30.10	28.57	133.16	135.99	129.25
7	26.53	28.65	25.75	30.42	29.23	127.68	128.07	124.95
8	28.60	30.39	27.91	34.35	33.07	145.08	147.12	145.06
9	29.11	31.97	28.28	33.26	30.87	145.22	150.81	145.46
10	25.99	27.90	26.03	37.17	35.69	114.81	116.95	114.52
Avrg.	27.27	29.50	26.84	35.11	33.65	142.61	146.04	141.65

Table S-XXXIV: Run Times of the fragmented calculations of 2M9E.

Conf.	Run Time / h							
	MFHC-[Nei2$_{4.0}$]			MIM-[$\eta = 9$]		PAIR$^{\mathrm{HB}}_{2.2}$-[Nei2$_{4.0}$]		
	[Hier2]	[Super]	[ee]	[Super]	[ee]	[Hier2]	[Super]	[ee]
1	27.40	29.24	26.09	32.06	30.08	100.20	101.70	97.86
2	23.98	26.41	22.88	31.73	30.18	98.40	100.43	96.20
3	29.89	31.66	28.56	32.35	31.48	96.59	98.13	93.37
4	27.06	29.28	25.68	33.70	31.34	97.50	100.26	97.23
5	26.81	27.96	25.31	29.36	27.97	101.03	100.96	98.28
6	25.30	27.11	24.01	32.22	30.58	114.51	116.68	112.52
7	29.90	31.00	28.40	30.08	28.69	97.77	96.23	94.34
8	28.67	29.97	27.36	31.01	29.81	99.59	98.28	96.74
9	27.63	28.84	26.20	30.58	28.75	114.93	115.22	110.86
10	28.39	29.63	26.90	34.37	33.11	110.40	113.15	107.89
11	26.77	30.39	25.17	34.11	31.08	109.50	112.24	104.84
12	26.62	29.18	25.52	33.78	31.31	88.58	90.71	86.82
13	27.71	30.74	26.68	36.16	33.01	104.77	106.90	101.99
14	27.66	31.23	26.53	34.08	30.48	105.99	108.35	103.97
15	29.72	31.93	28.30	31.64	29.69	106.51	105.81	102.74
16	30.81	33.37	29.56	29.79	27.75	98.04	100.59	95.54
17	27.06	31.10	25.59	36.81	32.47	93.47	97.45	90.15
18	26.98	30.48	25.78	35.73	32.68	104.75	106.85	101.61
19	27.28	30.39	25.49	32.39	29.52	98.37	102.14	95.54
20	25.64	29.33	24.47	32.79	29.55	110.34	111.70	105.51
Avrg.	27.56	29.96	26.22	32.74	30.48	102.56	104.19	99.70

Table S-XXXV: Run Times of the fragmented calculations of 2NC3.

Conf.	Run Time / h							
	MFHC-[Nei2$_{4.0}$]			MIM-[$\eta = 9$]		PAIR$^{HB}_{2.2}$-[Nei2$_{4.0}$]		
	[Hier2]	[Super]	[ee]	[Super]	[ee]	[Hier2]	[Super]	[ee]
1	23.15	29.95	22.76	37.30	31.57	90.05	93.88	88.45
2	23.45	26.77	23.30	35.19	32.80	94.42	97.50	93.10
3	24.46	27.57	24.29	34.26	32.39	90.27	93.42	88.30
4	24.03	27.58	23.45	36.86	33.82	97.68	101.03	97.30
5	23.67	26.38	23.49	35.36	33.48	88.12	92.40	87.52
6	23.71	26.09	23.15	33.80	32.33	89.60	90.57	87.02
7	23.97	26.93	23.14	37.31	35.01	102.76	103.68	99.74
8	23.71	26.83	22.84	34.03	31.43	95.13	96.67	92.82
9	23.71	27.22	23.38	34.96	32.32	94.88	96.13	91.02
10	23.84	26.79	23.85	35.56	33.48	95.85	98.87	94.70
11	23.40	26.48	22.63	35.83	33.83	101.73	105.27	101.60
12	23.68	27.18	23.19	34.47	32.01	103.78	107.40	104.25
13	23.37	26.23	23.28	32.20	30.56	91.18	94.65	89.96
14	23.30	26.06	23.04	32.59	30.61	88.25	89.80	85.66
15	24.57	27.34	23.77	37.16	34.68	107.28	111.03	104.43
16	23.66	26.42	22.90	34.47	32.69	86.93	88.32	84.06
17	23.55	27.76	23.38	34.18	31.38	110.18	111.98	105.76
18	23.24	27.14	23.08	37.30	33.88	100.42	104.15	99.30
19	23.44	26.01	22.78	32.82	30.80	85.80	88.26	85.31
20	23.22	26.22	22.42	34.38	32.31	90.33	92.44	89.06
Avrg.	23.66	26.95	23.21	35.00	32.57	95.23	97.87	93.47

Table S-XXXVI: Run Times of the fragmented calculations of 2KCF.

Conf.	Run Time / h							
	MFHC-[Nei2$_{4.0}$]			MIM-[$\eta = 9$]		PAIR$^{HB}_{2.2}$-[Nei2$_{4.0}$]		
	[Hier2]	[Super]	[ee]	[Super]	[ee]	[Hier2]	[Super]	[ee]
1	26.69	27.44	25.79	31.47	28.75	111.10	109.59	104.74
2	24.77	25.08	23.58	34.24	36.82	94.26	92.67	90.26
3	24.43	25.13	23.60	29.56	27.21	92.89	91.46	87.15
4	26.33	27.16	25.30	30.11	27.87	96.44	93.40	91.15
5	33.01	27.21	26.20	35.04	46.38	110.96	107.84	104.80
6	27.13	27.66	26.13	30.15	26.84	104.67	102.86	98.97
7	28.30	28.66	27.55	31.84	29.45	101.18	101.02	97.69
8	27.62	28.00	26.44	34.36	31.48	93.79	92.57	87.67
9	26.71	27.24	25.63	34.63	31.87	96.58	95.30	90.73
10	25.73	25.67	24.31	35.29	32.18	94.19	91.18	88.01
11	26.01	26.49	24.90	31.57	29.03	103.63	101.67	99.40
12	25.64	26.48	24.92	35.49	32.29	101.59	101.19	95.50
13	25.53	25.77	24.66	31.77	29.06	91.36	89.09	86.44
14	25.79	26.55	25.33	32.93	30.07	104.41	99.45	97.56
15	28.65	29.01	27.58	31.05	39.99	93.64	92.91	89.75
16	26.82	27.14	25.83	36.67	33.52	88.19	85.99	82.28
17	26.50	26.86	25.67	36.69	34.14	124.47	122.77	117.69
18	25.35	26.12	24.63	34.80	32.00	109.46	107.70	103.37
19	25.32	25.41	23.89	33.42	30.36	83.46	81.55	79.64
20	26.14	26.61	25.29	31.79	28.21	99.57	99.33	93.95
Avrg.	26.62	26.79	25.36	33.14	31.88	99.79	97.98	94.34

Table S-XXXVII: Run Times of the fragmented calculations of 2KYJ.

Conf.	Run Time / h							
	MFHC-[Nei2$_{4.0}$]			MIM-[$\eta = 9$]		PAIR$^{HB}_{2.2}$-[Nei2$_{4.0}$]		
	[Hier2]	[Super]	[ee]	[Super]	[ee]	[Hier2]	[Super]	[ee]
1	31.22	35.15	30.51	37.15	34.21	152.68	155.43	149.40
2	29.43	33.10	28.82	36.76	34.58	177.34	179.51	173.05
3	27.22	30.65	26.20	35.88	33.10	156.18	156.81	152.21
4	29.89	33.24	28.85	35.55	33.07	151.72	154.70	150.90
5	32.38	35.45	31.19	35.93	33.07	144.92	152.08	142.66
6	29.30	32.45	28.49	33.39	31.50	167.80	168.57	165.13
7	32.04	35.55	31.10	36.40	33.39	150.20	155.99	149.93
8	32.96	35.91	31.58	37.97	35.31	174.65	179.96	171.56
9	29.00	31.89	28.52	35.87	33.55	175.08	177.13	171.23
10	29.17	32.72	28.26	38.23	35.93	167.17	171.00	159.59
11	29.99	31.27	28.63	31.73	31.59	166.13	167.04	163.80
12	30.12	42.50	29.23	46.99	34.99	170.13	180.66	166.76
13	27.62	39.66	27.02	47.24	35.68	153.92	167.39	155.41
14	31.90	33.32	30.53	34.58	33.46	157.03	161.63	154.06
15	29.81	32.26	29.19	39.09	36.74	164.76	164.71	161.42
16	30.99	32.94	29.89	34.24	32.34	155.18	159.51	152.41
17	27.21	29.95	26.73	37.89	36.72	160.73	159.83	154.69
18	32.11	35.04	31.50	36.11	34.71	141.24	144.34	138.37
19	32.53	34.08	31.13	32.50	32.02	150.02	150.65	149.40
20	33.51	35.54	32.83	35.27	34.39	176.86	180.64	173.83
Avrg.	30.42	34.13	29.51	36.94	34.02	160.69	164.38	157.79

Table S-XXXVIII: Run Times of the fragmented calculations of 1AML.

Conf.	Run Time / h							
	MFHC-[Nei2$_{4.0}$]			MIM-[$\eta = 9$]		PAIR$^{HB}_{2.2}$-[Nei2$_{4.0}$]		
	[Hier2]	[Super]	[ee]	[Super]	[ee]	[Hier2]	[Super]	[ee]
1	21.58	20.98	19.71	44.06	50.73	73.39	72.87	67.18
2	20.71	20.02	19.42	46.27	53.79	60.77	58.99	56.67
3	18.19	18.14	17.17	41.68	39.37	69.60	65.56	64.38
4	18.98	18.39	17.68	43.62	41.09	62.76	60.18	57.63
5	20.74	20.27	19.48	44.09	40.73	56.48	55.18	52.60
6	19.49	19.07	18.23	42.95	55.01	54.94	53.08	49.92
7	21.30	20.36	20.21	44.07	56.85	70.36	68.81	63.71
8	19.70	19.58	18.56	48.45	45.04	63.73	61.01	58.89
9	18.16	17.79	16.78	45.79	42.98	58.98	56.31	54.65
10	18.03	17.93	16.85	46.46	43.00	52.91	51.28	48.22
11	18.10	17.82	17.10	42.64	39.81	50.58	47.03	46.56
12	19.98	19.91	18.88	42.62	61.23	64.44	61.99	59.21
13	19.34	19.29	18.05	45.31	42.30	73.98	47.67	46.23
14	19.71	19.04	18.21	48.15	70.15	58.53	56.79	53.81
15	19.60	19.17	18.16	46.32	64.89	61.39	58.64	57.05
16	19.26	19.37	17.77	46.56	64.77	61.10	58.50	57.26
17	20.21	19.69	18.72	45.71	42.28	77.10	75.49	70.48
18	19.98	19.19	18.89	43.43	40.63	62.52	59.87	57.45
19	20.24	19.95	19.00	45.06	65.02	52.46	49.58	48.28
20	20.26	20.29	18.84	45.42	41.99	61.36	58.66	56.97
Avrg.	19.68	19.31	18.39	44.93	50.08	62.37	58.88	56.36

Table S-XXXIX: Run Times of the fragmented calculations of 5KPH.

	Run Time / h				
Conf.	MFHC-[Nei$2_{4.0}$]		MIM-[$\eta = 9$]	PAIR$^{HB}_{2.2}$-[Nei$2_{4.0}$]	
	[Hier2]	[ee]	[ee]	[Hier2]	[ee]
2	73.80	65.17	101.57	334.82	306.78
4	68.09	60.92	88.10	304.12	278.22
5	67.60	61.67	97.63	297.43	273.47
6	73.05	66.33	96.19	286.52	266.10
8	71.20	64.19	98.50	313.94	285.11
9	72.00	64.21	90.10	292.27	267.46
10	70.51	64.49	97.12	302.46	277.36
12	72.93	65.52	92.86	323.73	292.36
13	68.52	62.06	96.08	288.21	260.50
14	73.68	65.13	94.74	303.48	277.17
17	76.22	69.14	95.28	323.12	298.22
20	71.42	63.15	102.90	281.10	256.52
Avrg.	71.59	64.33	95.92	304.27	278.27

Deviations of Relative Energies (B3LYP)

Table S-XL: Deviations of the relative energies of 1WN8.

Conf.	Energy Deviations / $kJmol^{-1}$								Rel. Energy / $kJmol^{-1}$
	MFHC-[Nei$2_{4.0}$]			MIM-[$\eta = 9$]		PAIR$^{HB}_{2.2}$-[Nei$2_{4.0}$]			Super
	[Hier2]	[Super]	[ee]	[Super]	[ee]	[Hier2]	[Super]	[ee]	B3LYP
1	56.11	44.36	40.28	61.02	35.79	42.89	63.93	42.58	-310.51
2	61.73	39.29	24.14	42.14	25.24	14.51	33.10	13.75	-205.65
3	51.19	42.11	54.51	14.56	77.90	77.70	69.29	48.00	-327.50
4	21.55	49.99	50.28	55.08	32.88	68.21	95.87	57.58	-205.00
5	59.11	18.88	8.23	31.81	6.89	17.16	32.41	30.81	-288.48
6	72.00	0.56	16.49	44.79	21.77	16.20	22.83	9.51	-342.23
7	99.84	83.38	68.45	49.99	93.50	64.96	63.05	67.72	-386.54
8	100.13	8.77	57.04	63.77	0.38	18.91	10.04	32.59	-177.93
9	6.97	68.74	49.90	61.38	91.64	70.40	96.38	58.11	-65.84
10	4.84	50.23	64.50	46.55	21.31	61.01	74.55	48.10	-149.43
11	0.71	15.57	20.36	89.12	35.47	1.05	10.24	27.69	-280.16
12	90.13	15.17	18.28	65.52	9.58	1.79	46.18	28.06	-283.40
13	10.69	31.29	5.35	15.08	53.58	9.92	33.24	5.89	-115.28
14	25.13	23.43	32.55	31.88	37.74	20.16	41.39	43.41	-160.28
15	66.08	60.72	33.05	91.21	16.87	29.99	49.68	23.54	-166.90
16	6.78	4.06	9.44	69.31	40.32	12.34	0.94	3.04	-147.56
17	27.31	16.06	26.79	42.58	9.12	39.26	61.03	14.22	-160.10
18	0.00	0.00	0.00	0.00	0.00	0.00	0.00	0.00	0.00
19	7.60	77.99	72.83	37.52	79.62	62.19	97.79	59.49	-33.97
20	11.28	9.49	3.82	48.65	53.23	28.71	21.89	23.56	-26.15
MAD	38.96	33.01	32.81	48.10	37.14	32.87	46.19	31.88	

Table S-XLI: Deviations of the relative energies of 1AML.

Conf.	Energy Deviations / $kJmol^{-1}$								Rel. Energy / $kJmol^{-1}$
	MFHC-[$Nei2_{4.0}$]			MIM-[$\eta = 9$]		$PAIR_{2.2}$-[$Nei2_{4.0}$]			Super
	[Hier2]	[Super]	[ee]	[Super]	[ee]	[Hier2]	[Super]	[ee]	B3LYP
1	137.69	17.41	68.11	102.90	71.84	33.14	31.59	21.60	-906.22
2	19.10	17.30	4.92	67.94	46.74	30.83	24.39	44.87	-419.33
3	53.07	14.94	5.66	70.99	46.40	41.66	20.82	30.07	-351.41
4	22.77	15.42	12.81	26.45	9.16	3.81	4.60	20.81	-406.13
5	0.00	0.00	0.00	0.00	0.00	0.00	0.00	0.00	0.00
6	63.30	0.08	10.42	56.12	23.58	4.24	11.01	4.42	-324.51
7	142.66	9.91	12.64	108.36	56.19	48.50	28.00	26.52	-445.28
8	8.96	11.68	47.12	20.27	5.79	28.41	7.68	86.40	-119.65
9	100.48	2.03	34.90	66.94	23.41	8.18	10.68	45.05	-350.22
10	54.29	14.92	21.04	4.42	9.94	21.44	15.72	7.34	-246.17
11	91.75	16.55	34.56	43.04	1.32	3.20	14.80	27.72	-124.39
12	99.21	7.65	47.35	30.29	1.36	14.00	4.04	46.43	-354.42
13	24.78	69.12	123.35	43.96	47.98	57.32	53.79	96.93	-152.00
14	67.31	54.89	78.11	24.43	22.67	43.26	33.40	38.68	-520.14
15	88.96	7.55	4.68	42.78	29.32	24.89	27.08	19.65	-348.15
16	8.57	76.44	82.39	56.90	60.85	56.83	70.35	103.50	-227.83
17	157.95	26.87	5.66	40.77	51.37	31.53	41.74	75.81	-673.08
18	94.37	23.92	50.71	30.69	5.72	2.35	21.08	16.48	-499.40
19	16.46	21.60	31.82	15.25	1.45	6.86	6.39	4.92	-514.48
20	6.36	80.91	105.92	15.76	45.43	56.91	71.14	89.05	-446.04
MAD	62.90	24.46	39.11	43.41	28.03	25.87	24.92	40.31	

Table S-XLII: Deviations of the relative energies of 2KCF.

Conf.	Energy Deviations / $kJmol^{-1}$								Rel. Energy / $kJmol^{-1}$
	MFHC-[$Nei2_{4.0}$]			MIM-[$\eta = 9$]		$PAIR_{2.2}$-[$Nei2_{4.0}$]			Super
	[Hier2]	[Super]	[ee]	[Super]	[ee]	[Hier2]	[Super]	[ee]	B3LYP
1	119.57	9.90	54.77	79.77	16.80	13.26	10.00	35.15	-582.89
2	66.03	4.09	19.32	25.11	1.83	10.72	13.64	55.30	-307.33
3	21.60	3.43	22.13	79.63	2.00	15.19	16.86	3.68	-345.72
4	75.51	2.28	57.28	93.89	4.88	11.83	4.08	48.62	-199.52
5	83.17	8.49	33.16	82.78	20.01	4.04	10.69	7.89	-501.25
6	79.58	3.06	31.29	102.67	3.91	12.77	5.24	11.36	-487.87
7	55.98	4.32	39.82	59.71	15.31	14.02	12.99	74.07	-604.30
8	42.13	10.66	3.04	161.80	12.52	28.46	6.86	11.47	-449.30
9	33.51	8.69	24.87	74.55	11.09	10.57	1.08	14.60	-358.35
10	2.56	5.66	14.64	17.13	21.23	18.12	3.46	14.30	-275.31
11	46.37	14.19	62.12	109.22	1.45	2.77	4.20	17.48	-335.03
12	40.05	10.69	37.98	66.75	4.72	9.12	8.60	52.38	-230.10
13	47.93	0.54	10.24	100.78	37.32	9.63	1.54	21.78	-305.64
14	183.16	9.09	17.41	82.88	17.58	3.16	10.28	43.99	-607.89
15	0.00	0.00	0.00	0.00	0.00	0.00	0.00	0.00	0.00
16	34.46	10.24	17.23	98.47	3.06	7.57	3.76	24.52	-382.90
17	99.18	11.25	44.11	56.91	6.25	0.28	2.89	7.97	-483.80
18	16.78	0.52	38.33	83.30	16.23	4.50	1.72	5.66	-435.66
19	57.11	7.24	33.73	32.73	6.55	5.54	1.90	4.69	-265.69
20	100.06	7.82	59.56	102.04	9.20	8.68	7.73	13.50	-702.13
MAD	60.24	6.61	31.05	75.51	10.60	9.51	6.37	23.42	
